# Multifunctional microelectronic fibers enable wireless modulation of gut and brain neural circuits

**DOI:** 10.1038/s41587-023-01833-5

**Published:** 2023-06-22

**Authors:** Atharva Sahasrabudhe, Laura E. Rupprecht, Sirma Orguc, Tural Khudiyev, Tomo Tanaka, Joanna Sands, Weikun Zhu, Anthony Tabet, Marie Manthey, Harrison Allen, Gabriel Loke, Marc-Joseph Antonini, Dekel Rosenfeld, Jimin Park, Indie C. Garwood, Wei Yan, Farnaz Niroui, Yoel Fink, Anantha Chandrakasan, Diego V. Bohórquez, Polina Anikeeva

**Affiliations:** 1 Department of Chemistry, Massachusetts Institute of Technology, Cambridge, MA USA; 2 Research Laboratory of Electronics, Massachusetts Institute of Technology, Cambridge, MA USA; 3 McGovern Institute for Brain Research, Massachusetts Institute of Technology, Cambridge, MA USA; 4 Laboratory of Gut Brain Neurobiology, Duke University, Durham, NC USA; 5 Department of Medicine, Duke University, Durham, NC USA; 6 Department of Electrical Engineering and Computer Science, Massachusetts Institute of Technology, Cambridge, MA USA; 7 Institute for Medical Engineering and Science, Massachusetts Institute of Technology, Cambridge, MA USA; 8 Secure System Platform Research Laboratories, NEC Corporation, Kawasaki, Japan; 9 Department of Chemical Engineering, Massachusetts Institute of Technology, Cambridge, MA USA; 10 Department of Materials Science and Engineering, Massachusetts Institute of Technology, Cambridge, MA USA; 11 Harvard/MIT Health Sciences and Technology Graduate Program, Cambridge, MA USA; 12 Institute for Soldier Nanotechnologies, Massachusetts Institute of Technology, Cambridge, MA USA; 13 Department of Neurobiology, Duke University, Durham, NC USA; 14 Duke Institute for Brain Sciences, Duke University, Durham, NC USA; 15 Department of Brain and Cognitive Sciences, Massachusetts Institute of Technology, Cambridge, MA USA

**Keywords:** Autonomic nervous system, Biomedical engineering, Sensors and biosensors

## Abstract

Progress in understanding brain–viscera interoceptive signaling is hindered by a dearth of implantable devices suitable for probing both brain and peripheral organ neurophysiology during behavior. Here we describe multifunctional neural interfaces that combine the scalability and mechanical versatility of thermally drawn polymer-based fibers with the sophistication of microelectronic chips for organs as diverse as the brain and the gut. Our approach uses meters-long continuous fibers that can integrate light sources, electrodes, thermal sensors and microfluidic channels in a miniature footprint. Paired with custom-fabricated control modules, the fibers wirelessly deliver light for optogenetics and transfer data for physiological recording. We validate this technology by modulating the mesolimbic reward pathway in the mouse brain. We then apply the fibers in the anatomically challenging intestinal lumen and demonstrate wireless control of sensory epithelial cells that guide feeding behaviors. Finally, we show that optogenetic stimulation of vagal afferents from the intestinal lumen is sufficient to evoke a reward phenotype in untethered mice.

## Abstract (web summary)

Crosstalk between the brain and gut is modulated and recorded with microelectronics fibers.

## Main

The extensive lines of bidirectional communication between the brain and visceral organs facilitate integration of internally arising interoceptive cues that are critical for survival. The gut–brain communication exemplifies one such important pathway wherein humoral and neural signals emerging from the abdominal viscera relay metabolic information to the brain for maintaining energy balance. Besides the well-known homeostatic functions, recent evidence suggests that the consciously imperceptible gut-to-brain signals can also modulate higher-level cognitive processes such as motivation, affect, learning and memory^1–5^. These findings create opportunities for co-opting such brain–organ neural circuits to develop minimally invasive autonomic neuromodulation therapies for otherwise intractable metabolic and neurological disorders such as treatment-resistant depression, obesity or diabetes^6,7^. However, identification of mechanisms underlying brain–viscera communication that influence neurocognitive states has remained challenging, which is at least in part due to the dearth of implantable bio-integrated multifunctional devices that allow safe, long-term deployment in anatomically and physiologically disparate organs of behaving animals. Traditionally, the fabrication of bio-integrated devices has relied on the use of resource-intensive lithographic techniques adapted from the semiconductor industry that require specialized cleanroom environments^8–15^. The thin film processing nature of lithography necessitates independent fabrication of individual modalities of the device stack, followed by careful manual assembly, thereby making this approach unsuitable for rapid customization^16–22^. Consequently, there remains a need for monolithic and scalable fabrication approaches that do not compromise the design flexibility, multimodality, functional sophistication and long-term biocompatibility of bioelectronic interfaces. Here we introduce a strategy that bridges this technological gap and demonstrate its potential in experiments spanning neural circuits in the brain and the gut.

**Fig. 1:**
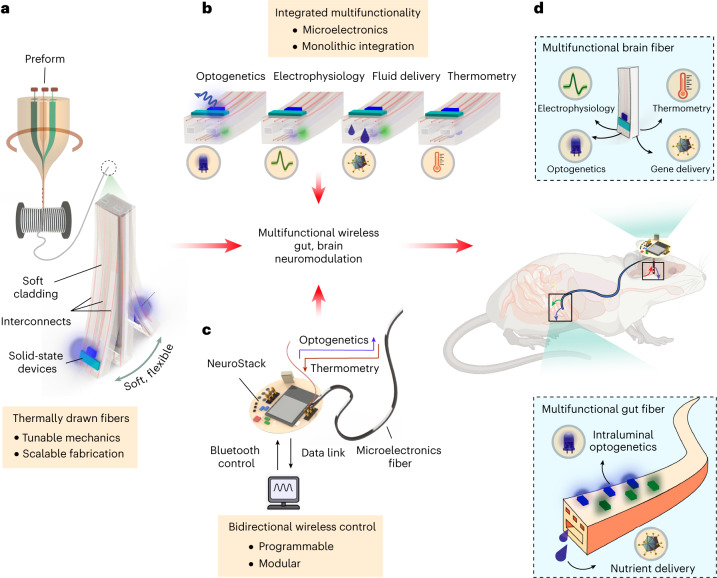
Schematic illustration of microelectronics-integrated multifunctional fibers that enable wireless modulation of brain and gut neural circuits. **a**, High-throughput, monolithic fabrication of multifunctional polymer fibers using thermal drawing yields several tens of meters of continuous fibers (~1,000 rodent-scale probes) with tunable mechanics and solid-state microelectronic components. **b**, Such fibers can host multiple independently addressable µLEDs for optogenetics, microelectrodes for extracellular electrophysiology, microfluidics for gene/chemical payload delivery and thermal sensors for tissue thermometry—all in a miniature footprint. **c**, NeuroStack, a custom designed modular wireless control circuit, enables real-time programmable optical stimulation and data transfer for recording of tissue temperature. **d**, The multifunctional microelectronic fibers together with NeuroStack allow for wireless modulation of neural circuits in the deep-brain and in the small intestine of awake behaving mice.

We develop multifunctional bioelectronic interfaces based on polymer fibers embedded with solid-state microelectronic components using thermal drawing^23^ (Fig. 1a). We leverage the top-down nature of thermal drawing to produce, in a single step, several tens of meters of microscale fibers (~1,000 rodent-scale probes) that can host: (1) surface localized microscale light emitting diodes (µLEDs) for optogenetics; (2) microscale thermal sensors for precision thermometry; (3) microelectrodes for electrophysiology; and (4) microfluidic channels for drug and gene delivery (Fig. 1b). We demonstrate that mechanical properties of such fibers can be engineered to produce architectures compatible with implantation in the deep-brain and the tortuous, mobile gastrointestinal tract. Furthermore, we also develop a modular wireless control circuit, NeuroStack, to interface with the microelectronic fibers (Fig. 1c)^24^ that permits real-time, programmable light delivery across multiple independent channels and wireless data transfer for recording of local tissue temperature in untethered behaving mice.

We demonstrate that microelectronic fibers can be chronically implanted into the brain and the intestine of mice (Fig. 1d). The stiff, yet flexible, fibers designed for the brain can accurately target deep-brain nuclei such as the ventral tegmental area (VTA), where we deliver a viral vector carrying channelrhodopsin-2 (ChR2) to dopaminergic (DA) neurons through the integrated microfluidic channel. The colocalized electrodes and μLEDs on the same fiber permit longitudinal recording of spontaneous and optically evoked neural activity following delivery of genetic payload, while thermal sensors enable concomitant deep-brain thermometry. On the other hand, the soft and compliant gut fibers capable of delivering light and nutrients in the intestinal lumen allow direct modulation of gastrointestinal neural circuitry, including epithelial sensory cells in the proximal and distal small intestine and the upper-gut innervating vagal afferents. Moreover, multisite devices permit simultaneous implantation of multifunctional fibers in the gut and the brain, allowing us to probe central neural representation of postingestive nutrient sensing. By coupling the VTA implanted fibers to NeuroStack, we show that wireless programmable optogenetic stimulation of DA neurons elicits a reward behavior. Similarly, the soft gut fibers enable wireless intraluminal gut optogenetics targeting the sparsely distributed enteroendocrine/neuropod cells in the duodenum and ileum that modulate feeding behavior. Finally, we uncover that optogenetic stimulation of vagal afferents from the gut lumen produces a rewarding phenotype, thereby demonstrating direct modulation of central nervous system function from the intestine in behaving mice. We anticipate that these illustrative applications will foreshadow widespread use of wireless multifunctional microelectronic fibers to study brain–viscera and multi-organ neural communication pathways.

## Results

### Multifunctional microelectronic fibers for the brain

**Fig. 2:**
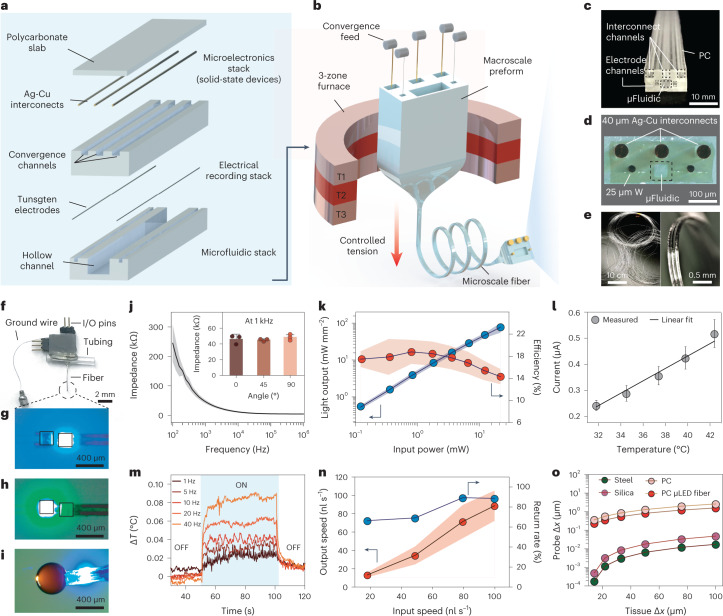
Fabrication and characterization of multifunctional brain fibers. **a**, Preform layout and assembly of a multifunctional brain fiber that comprises interconnect channels in the central PC layer and a precursor to microfluidic channel and recording electrodes in the bottom PC layer. **b**, Schematic of the thermal drawing process with simultaneous feeding of interconnect microwires (40 µm Ag-Cu) and recording electrodes (25 µm tungsten). **c**, Photograph of the preform highlighting channels for interconnects, electrodes and microfluidics. **d**, An optical micrograph of a fiber cross-section showing conserved features. **e**, Several meters of as-drawn fiber depicting the scalability of fabrication (left) and a close-up view of an acutely bent flexible fiber (right). **f**, A fully assembled multifunctional fiber device with I/O pins for microelectrodes and µLEDs, access tubing for microfluidic channel and a ground wire. **g**,**h**, Independently addressable blue (**g**) and green (**h**) μLEDs at the distal end of the device. **i**, Simultaneous microfluidic fluid delivery and blue µLED under operation at the device tip. **j**, Electrochemical impedance spectrum of the tungsten microelectrodes in 1 × PBS (*n* = 3 independent samples). Inset shows variation of electrode impedance with bending deformation. **k**, Optical intensity output (blue trace) and efficiency (red trace) of μLED (*λ* = 470 nm) integrated within a fiber with varying input electrical power (*n* = 6 independent samples), where arrows indicate the *y* axes for respective plots. **l**, Steady-state calibration curve of the in-fiber thermal sensor at 32–42 °C (*n* = 4 independent samples). **m**, Variation of local temperature as recorded by the thermal sensor during operation of an adjacently placed blue μLED on the same fiber at different stimulation frequencies (35.2 mW mm^−2^, 10-ms pulse). **n**, Characterization of the fiber microfluidic channel showing output speed (red trace) and return rate (blue trace) at varying input injection speeds (*n* = 4 independent samples), where arrows indicate the *y* axes for respective plots. **o**, Mechanical finite element model compares the displacement of the probe tip for steel, silica, bare PC fiber and microelectronic PC fiber at displacements of the brain tissue between 10 and 100 µm. All shaded areas and error bars represent s.d.; data are presented as mean ± s.d. µFluidic, microfluidic. Source data

To produce multifunctional microelectronic fibers for interrogation of brain neural circuits, we designed a multilayer polycarbonate (PC) preform (glass transition temperature *T*_g_ = 150 °C, Young’s modulus *E* = 1.8–3.2 GPa) (Fig. 2a) that was thermally drawn into a functional fiber (Fig. 2b, Supplementary Fig. 1a–d and Supplementary Video 1), while simultaneously feeding spools of interconnect (silver-copper, Ag-Cu, 40-µm diameter) and recording electrode microwires (tungsten, 25-µm diameter) (Supplementary Note 1). The overall cross-sectional geometry of the preform (Fig. 2c) was conserved during the draw (Fig. 2d), yielding ~50 m of functional fiber (Fig. 2e) with dimensions of 370.7 ± 2.8 µm × 190.4 ± 3.4 µm (mean ± s.d., *n* = 5 sections; Supplementary Fig. 1e). The fibers were assembled into implantable probes (Supplementary Fig. 2a–f and Fig. 2f) by mounting blue (peak emission wavelength *λ* = 470 nm) and green (*λ* = 527 nm) µLEDs (In_*x*_Ga_1−*x*_N, 270 × 210 × 50 µm^3^) along the fiber surface followed by deposition of a thin layer of parylene-C as a biofluid barrier coating. Optical micrographs of independently addressable µLEDs and microfluidic infusion in the final fiber device are shown in Fig. 2g–i. While the above approach shows integration of microelectronic components within the fibers postdraw, we also demonstrate embedding of semiconductor devices inside multifunctional fibers during fiber drawing, indicating further scalability of this platform (Supplementary Note 2 and Supplementary Fig. 3a–k)^25^.

### Characterization of brain fibers

Incorporation of tungsten microwires in fibers afforded low-impedance microelectrodes (|Z| = 46.3 ± 6 kΩ at 1 kHz) for electrophysiology without compromising device flexibility (Fig. 2j and inset). The electrode impedance exhibited a negligible increase upon immersion in phosphate-buffered saline (PBS) over 7 weeks, and no leakage current was observed through the polymer cladding (Supplementary Fig. 4a,b). The light intensity from the integrated blue µLEDs was tunable over a range between 0.6 mW mm^−2^ and 70 mW mm^−2^ (Fig. 2k), which is sufficient for optogenetic modulation of behavior mediated by microbial rhodopsins such as ChR2 (refs. ^26,27^). The robust bonding of µLEDs was corroborated by the stable light output even at large bending angles up to 90°, while long-term immersion tests in PBS demonstrated functionality for at least 7 weeks (Supplementary Fig. 4c,d). We applied finite element modeling (FEM) to investigate how illumination intensity and volume varied with distance from the μLED at different input intensities (Supplementary Fig. 5a–d). We find that even a moderate intensity of 30 mW mm^−2^ covers a tissue volume of ~0.75 mm^3^ near the fiber surface, sufficient for optogenetic modulation of most brain nuclei in mice.

We leveraged the temperature-dependent current-voltage (*I*–*V*) characteristics of the diodes (In_*x*_Ga_1−*x*_N µLED, *λ* = 470 nm) to record heat dissipation in the tissue during operation of the neighboring µLED^28^. A linear dependence of diode current on temperature defined the sensor calibration curve (Fig. 2l). Consistent with the thermal FEM (Supplementary Fig. 6a–d), the sensor detected a negligible temperature rise of 0.085 °C from a colocated µLED (*λ* = 470 nm, ~30 mW mm^−2^) operating at 40 Hz, which is well below the ~2 °C heating that occurs during clinical electrical deep-brain stimulation (Fig. 2m and Supplementary Fig. 7)^29^. To assess the microfluidic functionality, we measured the return rate of fluid infusion through the fiber (Fig. 2n) which was found to be in the range of 80–100% at physiologically relevant infusion speeds of 20–100 nl s^−1^ for intracranial injections. Since packaging of multiple functions in a neural probe can be at odds with achieving flexible device mechanics, we measured the bending stiffness of the fibers in the single-cantilever mode to mimic their anchoring to the skull. The fiber stiffness ranged between 25 and 33 N m^−1^, which is substantially lower than that of silica (132 N m^−1^, 0.4-mm diameter, 1-cm length) and stainless-steel (792 N m^−1^, 0.4-mm diameter, 1-cm length) probes of similar dimensions (Supplementary Fig. 8a). This was further corroborated through the mechanical FEM, which estimated a relative micromotion between the fiber tip and brain tissue that was ~2–4 orders of magnitude lower than silica and steel implants of similar dimensions (Fig. 2o and Supplementary Fig. 8b–f)^30^.

### Microelectronic fibers for the gut

**Fig. 3:**
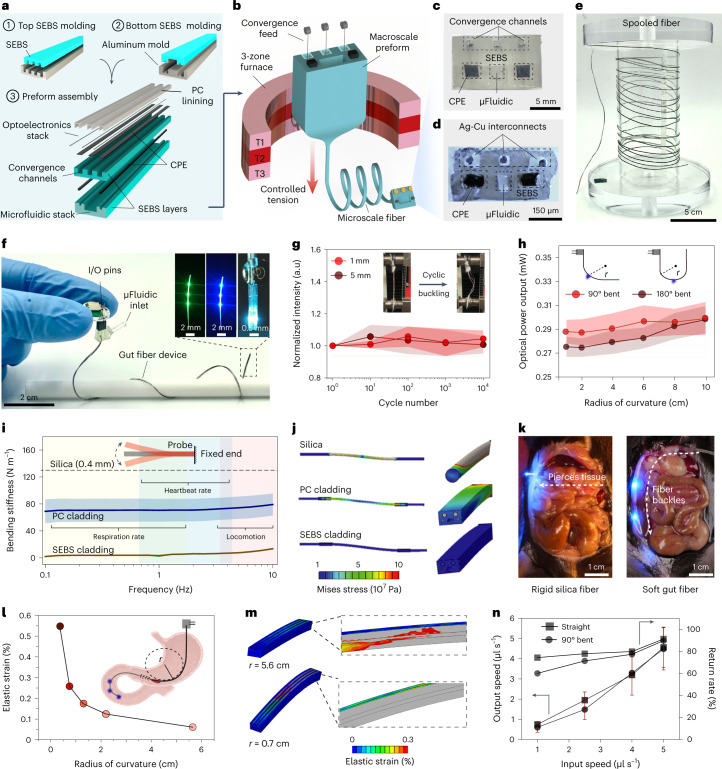
Fabrication and characterization of soft, multifunctional gut fiber. **a**, Layout of the multilayered gut fiber preform, where SEBS layers for convergence and microfluidic channel are molded from an inverse aluminum mold followed by preform assembly. **b**, The resultant preform is drawn into several meters of soft fiber using convergence-based thermal drawing. **c**, Digital image showing the cross-section of an assembled preform. **d**, Cross-sectional micrograph of the gut fiber highlighting conserved features. **e**, Several meters of as-drawn fiber wrapped around a spooler demonstrating scalable fabrication. **f**, Digital image of a multifunctional gut fiber device. Inset shows three green (left) and blue μLEDs (middle) on the fiber and dual optofluidic modality (right), where infusion of a PBS bolus is accompanied by operation of a blue μLED. **g**, Cyclic buckling of the gut fiber over 10^4^ cycles at 1-mm and 5-mm displacements and corresponding normalized light output from the fiber μLED (*n* = 3 independent samples). **h**, Light output from fiber μLEDs subjected to deformations at radii of curvature of 90° (*n* = 3 independent samples) and 180° angles (*n* = 3 independent samples). **i**, Bending stiffness of the fiber with SEBS cladding (*n* = 3 independent samples) and PC cladding (*n* = 3 independent samples) with identical cross-sections in comparison with a 400-µm silica waveguide (dashed). **j**, Mechanical FEM depicting stress distribution profiles in rigid silica (top), stiff PC (middle) and soft SEBS (bottom) fibers. **k**, Intraluminal implantation of a rigid silica fiber results in rupture of intestinal tissue (left), while soft gut fiber permits intestinal implantation that can negotiate the luminal curvature without tissue damage. **l**, FEM simulated elastic strain in Ag-Cu interconnects at varying radii of curvature. **m**, Corresponding spatial strain distribution in a gut fiber bent at radii of curvature of 5.6 cm (top) and 0.7 cm (bottom). **n**, Microfluidic return rate through the gut fiber at injection speeds between 1 and 5 µl s^−1^ relevant to intraluminal nutrient infusion in a straight and bent geometry (*n* = 3 independent samples). All shaded areas and error bars represent s.d.; data are presented as mean ± s.d. CPE, carbon doped polyethylene; *r*, radius. Source data

Unlike the brain, the gastrointestinal tract precludes implantation of rigid devices owing to a tortuous anatomy of the lumen which is encased in a delicate tissue, through which ingested food and fluids must pass^31^. Hence, we created multifunctional microelectronic fibers that are 10–15 times more compliant than the brain interfaces described above. These fibers permitted site-specific delivery of light and nutrients in the intestinal lumen of behaving mice. As their cladding (Fig. 3a), these fibers leveraged thermoplastic triblock elastomer poly(styrene-*b*-ethylene-*co*-butylene-*b*-styrene) (SEBS) (*T*_g_ = 140 °C, *E* = 3–5 MPa)^32^ which is ~10^3^ times softer than the PC cladding used in microelectronic brain fibers. A flexible conductive polyethylene composite was employed for integration of metallic interconnects within the elastomer cladding, and a thin layer of PC was applied to maintain structural integrity at the drawing conditions. The preform was drawn (Fig. 3b) into ~50 m of continuous microscale fiber (535 × 315 µm^2^) with a largely conserved cross-sectional geometry (Fig. 3c–e), while simultaneously incorporating interconnect microwires through convergence drawing. The fully assembled gut fiber devices (Fig. 3f) were ~8.5 cm long and hosted six µLEDs at the distal end that could be operated as two independently addressable sets of blue and green µLEDs (three each, Fig. 3f inset) and a microfluidic outlet situated ~0.8–1 mm posterior to the first µLED pair.

### Characterization of gut fibers

In the gut fibers, the surface-mounted µLEDs enable a laterally directed illumination profile which allows spatial targeting of epithelial cells and vagal afferents from within the lumen. This illumination profile is in contrast to an anatomically mismatched dorsal–ventrally oriented light cone of a typical silica fiber (Supplementary Fig. 9a). The cumulative light output from the three axially distributed µLEDs remained stable in PBS over at least 4 weeks (Supplementary Fig. 9b,c), while the optical output on the outer surface of the intestinal wall was only modestly attenuated by the presence of the intestinal tissue (Supplementary Fig. 9d). Using FEM, the optical penetration depth in the gut wall was found to be in the range 0.15–1 mm, and the illumination volume was estimated to be between 0.9 and 8.8 mm^3^ for input intensities ranging from 20 to 100 mW mm^−2^ (Supplementary Fig. 10a–f). This is sufficient to broadly illuminate the subepithelial mucosa layer 50–100 µm beneath the mucosal membrane that receives dense vagal innervation^33^. The corresponding temperature change in the gut wall was found to be negligible for input optical intensities between 20 and 100 mW mm^−2^, while the µLED separation (~1 cm) was sufficient to prevent any co-operative heat buildup (Supplementary Fig. 11a–e).

As the gut continuously undergoes peristaltic distortion, we performed cyclic buckling tests to assess the mechanical integrity of the fibers at deformations of 1 mm and 5 mm for up to 10^4^ cycles, which had no impact on device performance (Fig. 3g). The fiber robustness was further supported by negligible changes in light output during 90° and 180° bending deformation at radii of 2–10 cm, which corresponds to strains exceeding those experienced by devices during surgical implantation process (Fig. 3h). We hypothesized that the mechanical compliance of the gut fiber would minimize the force exerted on the intestinal lumen, which is critical for chronic bio-integration in behaving animals. To mimic a surgically implanted gut fiber affixed to the abdominal wall at one end, their stiffness was evaluated in a single-cantilever bending mode. The gut fibers exhibited stiffness of 2–5 N m^−1^ across frequency ranges of heartbeat, respiration, locomotion and peristalsis (Fig. 3i), which is considerably lower than the stiffness of identical fibers composed entirely of stiffer plastic such as PC (70–75 N m^−1^) and similarly sized commercial silica fibers (400-µm diameter, 132 N m^−1^). FEM of stress distribution profiles also qualitatively captured these experimentally observed stiffness trends (Fig. 3j). Unsurprisingly, during an intraluminal implantation in a mouse small intestine, the rigid silica fiber punctured the mucosal membrane, and thus was unsuitable for in vivo use, whereas the soft gut fiber readily negotiated the lumen curvature without damaging the epithelial tissue (Fig. 3k). Since the surgical procedure requires bending of the gut fiber at acute radii, we simulated strain distribution in the copper interconnects which have the lowest yield strain among the fiber constituents and confirmed the strain to be below the elastic limit of 0.3% for radii >0.5 cm (Fig. 3l,m). Finally, fluid infusion through the microfluidic channel of gut fiber at injection speeds relevant to intestinal nutrient delivery (1–5 µl s^−1^)^34^ yielded a high return rate in the range of 60–90% under both straight and bent conditions (Fig. 3n).

### Wireless operation of microelectronic fibers

**Fig. 4:**
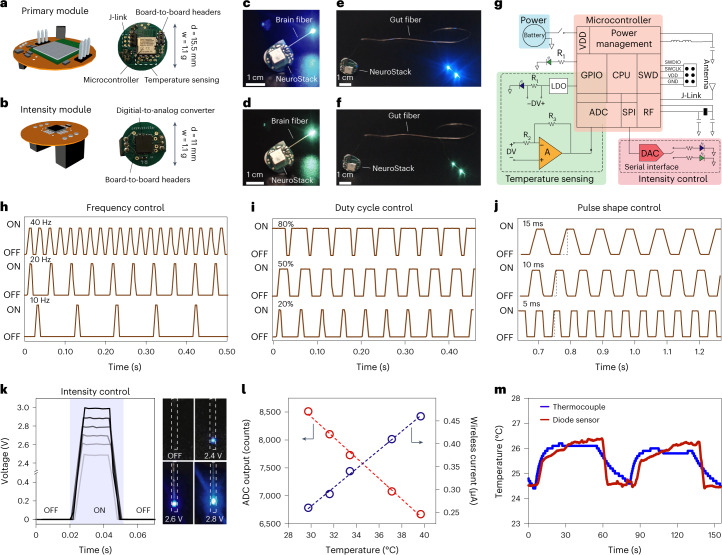
NeuroStack, a custom designed modular wireless circuit for microelectronic fibers, allows programmable light delivery and physiological recording. **a**,**b**, Schematic illustration (left) and digital images (right) highlighting important circuit components of a primary module (**a**) and intensity module (**b**). **c**–**f**, Images showing independent wireless control of blue (**c**) and green (**d**) µLEDs within a brain fiber and blue (**e**) and green (**f**) µLEDs in the gut fiber. **g**, Circuit layout of NeuroStack highlighting the power management block, temperature sensing block and intensity control block. **h**, Real-time control of optical stimulation frequency between 10 and 40 Hz. **i**, Real-time control of optical stimulation duty cycle between 20% and 80%. **j**, Optical pulse shaping through control of pulse rise and fall times between 5 and 15 ms. **k**, Intensity module permits real-time control of µLED brightness with corresponding photographs at 2.4-, 2.6- and 2.8-V bias. **l**, Calibration of the fiber thermal sensor using NeuroStack under steady-state conditions of 29–40 °C. **m**, Wireless recording of temperature transients with the thermal sensor in the microelectronic fiber and its comparison with a commercial thermocouple in wired mode. d, diameter; w, weight; CPU, central processing unit; DAC, digital-to-analog converter; GND, ground; GPIO, general-purpose input/output; LDO, low-dropout regulator; RF, radio frequency; SPI, serial peripheral interface; SWCLK, serial wire clock; SWD, serial-wire debug; SWDIO, serial-wire debug data input/output ; VDD, voltage drain-to-drain; ADC, analog-to-digital converter. Source data

Incorporation of microelectronics in polymer fibers provides an opportunity for wireless bidirectional operation that can facilitate behavioral assays in untethered subjects. To realize this, we engineered a miniature (15.5 mm), light-weight (1.1 g) modular platform^24^, NeuroStack, that enabled programmable wireless optical stimulation across two independent channels and data transfer for real-time temperature recording (Fig. 4a). The circuit features a Bluetooth Low Energy (BLE) communication protocol via a 2.4-GHz wireless link and an on-board miniature rechargeable battery for stable operation. This not only allows easy deployment in animal behavior studies with minimal user intervention, but also permits real-time programming of up to four devices across independent channels without any specialized equipment from a computer connected to an nRF52840 Development Kit that acts as a base station. The modular design of the circuit enables straightforward customization of experiments and allows integration of additional functions (Fig. 4b) within the same area footprint which is limited in small rodents. Similarly, the detachable architecture of the module obviates the need for the animals to carry subdermal electronics which are prone to misalignment and malfunction (see Supplementary Note 3 for additional discussion).

The primary module of NeuroStack carries an MDBT42V wireless microcontroller (with Nordic nRF52832 chip) and a rechargeable battery for orientation-independent power supply (Fig. 4a, Supplementary Fig. 12a–d and Supplementary Videos 2–4). During operation, the primary module relies on the microcontroller’s general-purpose input/output (GPIO) pins to drive current through the fiber μLEDs (Fig. 4c–f), while the header pins at the top support attachment of an intensity module (Fig. 4b). The intensity module can control the intensity of optical stimulation through a digital-to-analog converter which is programmed from the serial interface and allows transient shaping of optical stimulation pulses, a feature that is important for minimizing coupled electromagnetic artifacts in electrophysiological recordings during optical stimulation. The temperature sensing circuit uses one of the in-fiber μLEDs as a temperature sensor, whereas the other channel is assigned for optical stimulation via the second μLED. A constant voltage below the turn-on voltage is applied to the sensor diode using a low-dropout regulator to measure current variations. The sensed current is then amplified using a single-stage inverting differential amplifier. The internal analog-to-digital converter of the microcontroller then digitizes the amplified analog signal and transmits it wirelessly to the computer. The default sampling rate for temperature recording is set to 200 Hz and the temperature sensing function can also be turned off through the system software to save power when not needed. In addition to the main components, the module also hosts a 32-kHz crystal oscillator and inductors for power management. The device is programmed/debugged with a 6-pin J-link interface and controlled remotely through an intuitive software platform (see the Methods section for software details). The NeuroStack power breakdown (Supplementary Fig. 12e,f) scales with the µLED duty cycle and other functions including temperature sensing and intensity control. Figure 4c–f shows wireless operation of the independently addressable µLEDs within the brain and gut fibers connected to NeuroStack. The complete electrical layout of the primary module, together with temperature sensing and intensity control circuits, appears in Fig. 4g. We characterized the capability of NeuroStack to control the frequency, duty cycle, pulse shape and intensity of optical stimulation in real-time from the user interface. The creation of the stimulation pulse consists of two phases controlled by software timers. One of the timers manages the change in state between pulsing and resting period, thereby controlling frequency of optical stimulation (Fig. 4h), while the other timer sets the duty cycle during the pulsing period (Fig. 4i). The same principle is used for the intensity control to set the maximum intensity and rise/fall times of the pulses (Fig. 4j,k). The wireless temperature recording function was evaluated via steady-state measurements on a hotplate which showed linear dependence of the measured current on the surrounding temperature (Fig. 4l), while the dynamic response upon successive immersions in a temperature-controlled water bath matched closely to that of wired recordings from a commercial thermocouple (Fig. 4m).

### Multimodal interrogation of midbrain DA neurons

**Fig. 5:**
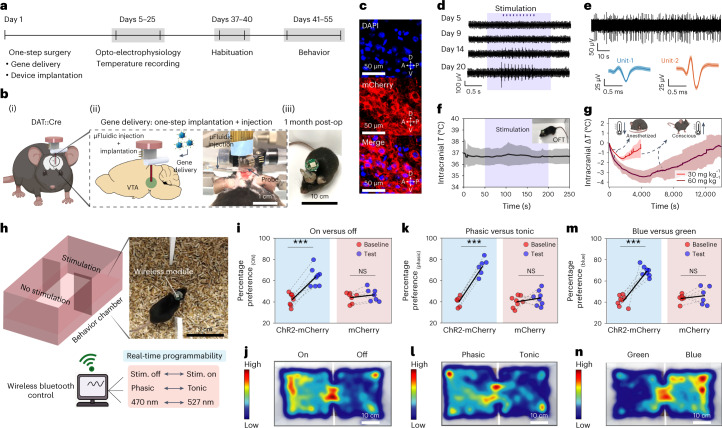
Multimodal interrogation of deep-brain neural circuit and wireless programmable optogenetics during behavior. **a**, Experimental timeline for in vivo validation of various fiber functionalities. **b**, Gene delivery through the microfluidic channel and fiber implantation in the same surgical procedure (i); photograph of one-step surgery highlights the fluid injection setup and the implanted fiber probe (ii); fully recovered animal ~1 month postsurgery carrying a wireless module (iii). **c**, Expression of Cre-dependent ChR2-mCherry construct 4 weeks following microfluidic AAV5 delivery into the VTA of DAT::Cre mice; (top) blue, DAPI; (middle) red, mCherry; (bottom) merge. **d**, Longitudinal electrophysiological recording of optically evoked neural activity in the VTA following AAV5 delivery. **e**, Spontaneous neural activity recorded from VTA at week 4 postimplantation (top); corresponding average action potential waveforms of two isolated neurons (bottom). **f**, Wireless intracranial temperature recording in mice (*n* = 6) exploring an open-field chamber during simultaneous wireless photostimulation (shaded region). **g**, Fiber thermal sensors detect brain hypothermia in the VTA induced by intraperitoneal injection of ketamine-xylazine mixture at 30-mg kg^−1^ (*n* = 3 mice) and 60-mg kg^−1^ (*n* = 3 mice) doses. **h**, Place preference assay during real-time wirelessly programmable optical stimulation in DAT-Cre mice. **i**,**k**,**m**, Percentage preference to the chamber coupled to the rewarding optical stimulation at baseline and on test day for mice transduced with ChR2-mCherry or mCherry in the VTA at three different wireless photostimulation conditions (top). **i**, On versus off. ChR2-mCherry: *P* = 4.16 × 10^–5^, *t* = –9.03, d.f. = 7; mCherry: *P* = 0.183, *t* = –1.50, d.f. = 6. **k**, Phasic versus tonic. ChR2-mCherry: *P* = 2.02 × 10^–4^, *t* = –9.65, d.f. = 5; mCherry: *P* = 0.403, *t* = –0.88, d.f. = 7. **m**, Blue-light versus green-light stimulation. ChR2-mCherry: *P* = 3.58 × 10^–5^, *t* = –9.24, d.f. = 7; mCherry: *P* = 0.55; *t* = –0.64, d.f. = 5 (**P < 0.01; ***P < 0.001; NS, *P* > 0.05; two-tailed paired samples *t*-test). **j**,**l**,**n**, Representative heat-maps tracing the animal position corresponding to the assays summarized in panels **i**, **k** and **m**, respectively. All shaded areas and error bars represent s.d., data are presented as mean ± s.d. A, anterior; D, dorsal; NS, not significant; OFT, open-field test; P, posterior; Stim., stimulation; V, ventral. Source data

The combined optical, electrical, fluidic and thermometry functions in the brain fibers enabled multiple experiments in mice for at least 2 months following implantation (Fig. 5a). As a validation study, we first targeted the DA neurons in the VTA, a key node of the reward and motivation pathways^35^. The fibers were stereotactically implanted in the VTA of DAT::Cre transgenic mice which express Cre-recombinase under the dopamine transporter (DAT) promoter. The integrated microfluidic channel within the fiber permitted delivery of an adeno-associated virus (adeno-associated virus serotype 5 (AAV5)) carrying ChR2 gene in a Cre-dependent construct (Ef1α::DIO-ChR2-mCherry) or a control construct (Ef1α::DIO-mCherry) to the VTA during a single-step surgery (Fig. 5b(i–iii))^36^, and robust expression of ChR2 was observed in the VTA sagittal sections (Fig. 5c). Emergence of electrophysiological potentials recorded through integrated microelectrodes in response to optical stimulation via fiber µLEDs revealed the time course of Cre-dependent opsin expression in DA neurons (Fig. 5d and Supplementary Fig. 13). As it is known that recording electrodes may exhibit optical stimulation artifacts (for example, Becquerel effect)^37^, we confirmed the physiological origins of optically evoked signals and devised an artifact mitigation strategy by transient pulse shaping (Supplementary Figs. 14a–i and 15a–h and Supplementary Note 4). Optically evoked multiunit neural activity was reliably recorded for at least 2 months in awake moving mice (Supplementary Fig. 16a–o). We hypothesized that the flexible fibers could enable stable recording of spontaneous single neuron activity over extended periods due to their reduced micromotion relative to brain tissue. After confirming the functional stability of implanted electrodes up to 6 months (Supplementary Fig. 17a,b), we recorded the spontaneous single-unit activity from putative VTA neurons in chronically implanted mice for 4 weeks (Fig. 5e and Supplementary Fig. 18). Additional examples of single neuron electrophysiology at weeks 2 (*n* = 3 mice) and 4 (*n* = 3 mice) appear in Supplementary Figs. 19a–i and 20a–i, respectively.

To confirm device safety, we wirelessly recorded intracranial temperature with the embedded thermal sensors in freely moving animals in an open-field chamber and found no significant changes in tissue temperature during simultaneous wireless optical stimulation (Fig. 5f). The long-term biocompatibility of the fibers was assessed via immunohistochemical analysis (Supplementary Fig. 21a–l) of markers characteristic of glial scarring (activated macrophage marker ionized calcium-binding adaptor molecule 1 (Iba1), astrocytic marker glial fibrillary acidic protein (GFAP)). As expected from FEM and experimentally measured lower bending stiffness, the immune response to microelectronic fibers was lower than that of silica fibers of comparable size (300 µm) across both inflammatory markers at week 2 (ref. ^30^).

We next evaluated the ability of the thermal sensors to detect physiologically evoked changes in intracranial temperature induced by an anesthetic drug mixture (ketamine-xylazine) which is known to trigger hypothermia by inhibiting thermoregulatory responses in a dose-dependent manner^38^. In mice that were given intraperitoneal injections of ketamine-xylazine (9:1 ratio) at two concentrations (30 mg kg^−1^ and 60 mg kg^−1^ of ketamine), the fibers reliably recorded a drop in the intracranial temperature (Fig. 5g and Supplementary Fig. 22a,b). The temperature recovery to preanesthesia level was commensurate with the animals gaining consciousness and ambulating in the homecage. The cointegration of thermal sensors and recording electrodes within the same fiber may facilitate understanding of how changes in brain temperature alter neural dynamics under anesthesia^39^.

To illustrate the wireless programmable photostimulation capability during behavioral studies, mice expressing ChR2 in the DA neurons in the VTA were subjected to a real-time place preference (RTPP) task (Fig. 5h)^35^. The use of the BLE communication protocol allowed programming of optical parameters without any line-of-sight or angular orientation handicap. To illustrate this, we performed RTPP tasks at three different stimulation conditions that were updated in real-time: (1) stimulation ON (*λ* = 470 nm, 25 Hz, 10-ms pulse, 1 s ON, 2 s OFF) versus OFF; (2) phasic bursting (*λ* = 470 nm, 40 Hz, 5-ms pulse, 0.5 s ON, 4 s OFF) versus tonic stimulation (*λ* = 470 nm, 5 Hz, 5-ms pulse); (3) blue-light (*λ* = 470 nm, 25 Hz, 10-ms pulse, 1 s ON, 2 s OFF) versus green-light stimulation (*λ* = 527 nm, 25 Hz, 10-ms pulse, 1 s ON, 2 s OFF). The mice expressing ChR2 in DA neurons in the VTA exhibited a significant preference (Fig. 5i,k,m and Supplementary Fig. 23a–f) for the chamber paired with the stimulation condition as compared with their baseline values (pretest day), which was not observed in the control mice expressing mCherry alone. Figure 5j,l,n shows the representative heat-maps of the animal positions in the RTPP arena in each experimental condition. Furthermore, we did not observe any significant differences in the average velocity and distance traveled by device-carrying mice as compared with naïve counterparts, and no significant changes in locomotor activity were observed in response to photostimulation of the VTA ChR2-expressing DA neurons (Supplementary Fig. 24a–e and Supplementary Video 5). The µLEDs embedded in the fiber also remained functional for at least 9 months following implantation (Supplementary Fig. 24f).

### Multimodal interrogation of gut–brain communication

Optogenetics and pharmacology have revolutionized studies of brain circuits. However, the extension of these methodologies to neural circuits in the gut has remained challenging. We hypothesized that our soft multifunctional, microelectronic gut fibers capable of targeted light and chemical delivery along the gastrointestinal tract can address this challenge.

**Fig. 6:**
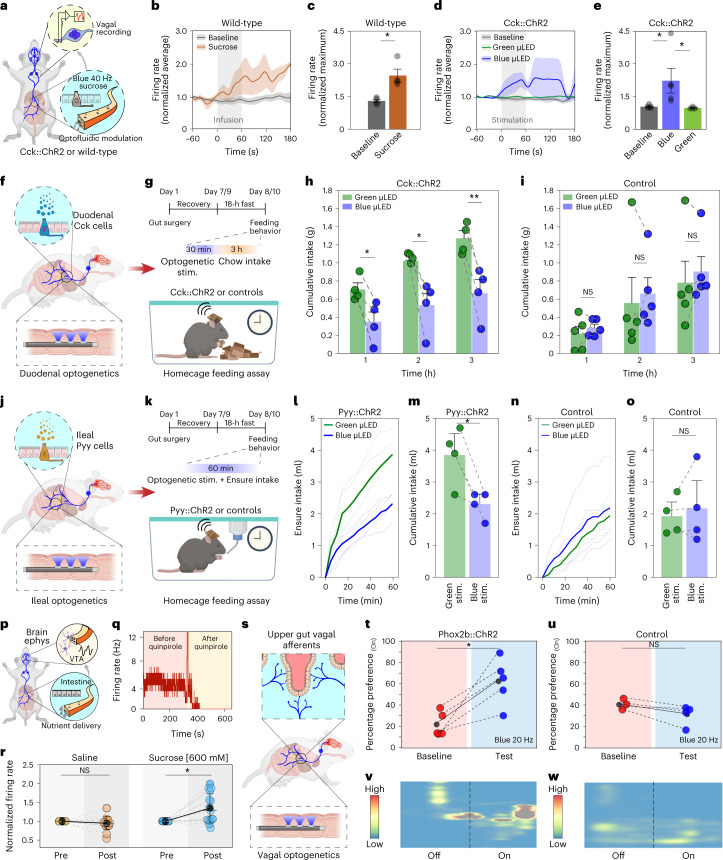
Multimodal interrogation of gut neural circuits and wireless programmable optogenetics during behavior. **a**, Vagal electrophysiology during intestinal optofluidic modulation. **b**, Duodenal sucrose (300 mM) increases vagal firing rate. **c**, Quantification of peak responses (*n* = 4; *P = 0.0304 by Kruskal–Wallis test with nonparametric comparisons using Wilcoxon method). **d**, Optogenetic stimulation of Cck^+^ cells increases vagal firing rate. **e**, Quantification of peak responses; *P < 0.0367 by Kruskal–Wallis test with nonparametric comparisons using Wilcoxon method, baseline (*n* = 5) versus blue µLED (*n* = 5): *P* = 0.0367; baseline versus green µLED (*n* = 4): *P* = 0.1113; blue µLED versus green µLED: *P* = 0.0200. **f**,**g**, Schematic of wireless intraduodenal optogenetic control of Cck^+^ cells (**f**) while evaluating feeding behavior (**g**). **h**,**i**, Chow intake in (**h**) Cck::ChR2 mice (*n* = 4, significant effect of time (*P* = 0.0003), stimulation (*P* < 0.0001) and time × stimulation interaction (*P* = 0.0086); post hoc two-sided paired *t*-tests: 1 h, *P* = 0.0161; 2 h, *P* = 0.0376; 3 h, *P* = 0.0044) and for (**i**) control mice lacking ChR2 (*n* = 4, significant effect of time (*P* = 0.0020), but not stimulation (*P* = 0.4975) or time × stimulation interaction (*P* = 0.8906)). **j**,**k**, Illustration of ileal optogenetic control of Pyy^+^ cells (**j**) while evaluating food intake (**k**). **l**, Ensure intake for Pyy::ChR2 mice during wireless optical stimulation (*n* = 4, significant effect of time (*P* < 0.0001), stimulation (*P* < 0.0001) and time × stimulation interaction (*P* < 0.0001)). **m**, Cumulative intake of **l** (two-sided paired *t*-test, *P* = 0.0381). **n**, Ensure intake for control mice (*n* = 4 mice, significant effect of time (*P* < 0.0001) and stimulation (*P* = 0.0160), but no significant time × stimulation interaction (*P* = 0.5796)). **o**, Cumulative intake for **n** (two-sided paired *t*-test, *P* = 0.4639). **p**, Schematic of brain VTA electrophysiology during duodenal microfluidic infusion. **q**, Firing rate of a putative DA neuron is sensitive to quinpirole. **r**, Intraduodenal sucrose increases firing rate of putative DA neurons compared with saline (saline: *n* = 20; sucrose: *n* = 18 neurons; two-sided Wilcoxon signed-rank test, *P* = 0.8595, *P* = 6.71387 × 10^−4^, respectively). **s**, Schematic of duodenal optogenetic stimulation in Phox2b::ChR2 mice. **t**,**u**, Percentage preference at baseline and on test day for Phox2b::ChR2 (**t**) and control mice lacking ChR2 (**u**) (two-sided paired *t*-test, Phox2b::ChR2: *P* = 0.014, *t* = 4.19, d.f. = 4; control mice: *P* = 0.110, *t* = –2.25, d.f. = 3). **v**,**w**, Representative heat-maps of animal position corresponding to **t** (**v**) and **u** (**w**). All data are represented as mean ± s.e.m, except in **r** which shows mean ± s.d. Ephys, electrophysiology. Source data

The sensory cell of the gut epithelium is the enteroendocrine cell, also known as the neuropod cell^40,41^. Although enteroendocrine cells have been historically studied in the context of hormone release in response to nutrient stimuli, it was recently shown that these cells synapse with the vagus nerve and transduce signals to the brain within milliseconds^34,40,42^. To acknowledge their neurotransmission function, we refer to these cells as neuropod cells. In spite of the importance of these cells in providing critical inputs for driving behavior directly from the gut, there are only a few studies subjecting them to targeted optogenetic and pharmacological manipulations to unravel their role in feeding behavior^34,43^. We first used these epithelial sensory cells as a testbed to evaluate the dual optofluidic modality of fibers using cervical vagus nerve electrophysiology as a readout (Fig. 6a and Supplementary Fig. 25a). In wild-type mice, we found that intestinal infusion of sucrose solution (300 mM, 0.2 ml, 3.3 µl s^−1^) via the microfluidic channels of the implanted fibers resulted in a significant increase in the vagal firing rate as compared with baseline activity (Fig. 6b,c). This effect of chemical stimulation was recapitulated in transgenic mice expressing ChR2 in cholecystokinin (Cck) cells (Cck::ChR2), where optogenetic excitation via the blue µLEDs (40 Hz, 10-ms pulse width) within gut fibers resulted in an increased vagal firing rate. Vagal responses remained unchanged in Cck::ChR2 mice upon illumination with the green µLEDs integrated within the same devices as well as in control littermates that lacked ChR2 in Cck cells (Fig. 6d,e and Supplementary Fig. 25b). Since the gut is also innervated by nociceptive vagal afferents that harbor temperature-sensitive ion channels (for example, transient receptor potential vanilloid family member 1)^44^, it raises the concern that tissue heating during µLED operation in the gut lumen may lead to off-target activation of nociceptive terminals, thereby compromising the cell-type specificity afforded by intraluminal gut optogenetics. To address this concern, we performed wireless in vivo temperature recordings during the optical stimulation epoch in awake, chronically implanted mice and found no substantial changes in local temperature as predicted by our FEM studies (Supplementary Fig. 25c).

Neuropod cells are present along the entire alimentary tract, where they release hormones and neurotransmitters to regulate food intake^41,45^. To evaluate the impact of neuropod cells on feeding behavior, we implanted the soft gut fibers in two distinct gastrointestinal regions: the duodenum and the ileum. Since the proper function of the intestinal lumen is critical for survival, we first evaluated whether chronic implantation of the multifunctional fibers in the gut affected animals’ food and water intake as well as locomotor activity. We found no significant changes in these measures before and after fiber implantation (Supplementary Fig. 26a–c), thereby confirming that the implanted fibers do not obstruct the passage of ingested food or fluids and do not interfere with physiological activity (Supplementary Videos 6 and 7). The biocompatibility of chronically implanted gut fibers was also evaluated through histological analysis of the gut tissue, which did not show significant changes in the anatomical characteristics of the epithelial layer (Supplementary Fig. 27a–d). This indicates that, commensurate with their soft mechanics, the chronically implanted gut fibers do not compromise the integrity of the fragile epithelial barrier.

It is well known that exogenous administration of the satiety hormone cholecystokinin (CCK) produces robust anorexia in rodents and humans^46,47^. CCK is also released from the neuropod cells in the small intestine in response to nutrients entering the gut^48^. Thus, we hypothesized that optogenetic stimulation of duodenal Cck-expressing neuropod cells will produce an enduring anorectic effect which can be measured by alterations in food intake. To test this hypothesis, the soft gut fibers were implanted in the duodenum of Cck::ChR2 mice (Fig. 6f,g). The multiple independently controlled stimulation channels on the gut fiber allowed a within-subject experimental design for these studies (Methods). After an overnight fast, stimulation of duodenal Cck cells (Supplementary Fig. 28a,b) with blue µLEDs (20 Hz, 10-ms pulse width, 0.5 s ON, 1 s OFF) significantly suppressed chow intake for 3 h, compared with control green-light stimulation (Fig. 6h). Blue-light stimulation had no impact on food intake in mice lacking ChR2 expression compared with within-subject green illumination control (Fig. 6i).

In the ileum, the presence of fat and carbohydrates induces the ileal brake, which is a neuro-hormonally mediated feedback loop that regulates gastrointestinal emptying rates by decreasing food intake^49^. Previous studies employing nutrient infusion into the ileum or exogenous administration of neuropeptides indicated that peptide YY (PYY) and glucagon-like-peptide 1 (GLP-1) mediate the effects of the ileal brake^50,51^. However, these findings were correlational as limited approaches were available to establish a causal link between specific cell types in the ileum to the physiological feedback loop^52,53^. We reasoned that direct optogenetic activation of Pyy^+^ cells in the ileum, which release PYY and GLP-1, through a chronically implanted gut fiber may further implicate these cells as mediators of the ileal brake. To ensure a stable optical power output from gut fibers during hour-long ileal feeding assays, the wireless circuit was modified to include a low-dropout voltage regulator which supports higher voltage, and higher capacity, light-weight rechargeable batteries (Supplementary Fig. 29a–d). The soft gut fibers were implanted in the ileum of Pyy::ChR2 mice which were allowed to recover for 1 week postsurgery and fasted for 18 h before the experiment (Fig. 6j,k, within-subject experimental design). Wireless stimulation of ileal Pyy neuropod cells (Supplementary Fig. 30a,b) with blue µLEDs (20 Hz, 10-ms pulse width, 0.5 s ON, 1 s OFF) caused a significant suppression of consumption of the high-fat and -carbohydrate solution (Fig. 6l,m) compared with control green light, while blue-light stimulation in mice lacking ChR2 in Pyy ileal cells had no impact on intake compared with within-subject controls (Fig. 6n,o). We note that baseline intake is lower in mice lacking ChR2 (Methods); however, there was no effect of blue light on total intake in the within-subject designs. Together, the control of vagal activity as well as the feeding behavior upon intraluminal optofluidic stimulation in the proximal and distal small intestine highlight the efficacy of multifunctional gut fibers in modulating the sparsely distributed intestinal cells (~1% of epithelial cells)^45,54^.

Food intake is regulated by bidirectional and coordinated gut–brain signaling^55^. While the effects of gut-derived signals on satiety are extensively studied, their influence on neural populations driving food intake is not fully understood^56^. We reasoned that recording single-unit neural activity through the brain fibers during intestinal microfluidic delivery of nutrients via the gut fibers may open future possibilities in probing how nutrient detection in the gut is encoded in the brain. For instance, it was previously shown that feeding-induced DA release in the ventral striatum can be driven by postingestive feedback in a nutrient-specific manner, independent of oro-sensory cues^57,58^. Since the ventral striatum receives dense innervation from the VTA DA neurons, we hypothesized that intraintestinal delivery of sucrose can positively modulate firing rate of DA neurons in the VTA. To test this hypothesis, wild-type mice were implanted with brain fibers in the VTA and electrophysiological recordings were performed 1 week following surgery (Fig. 6p). The identity of putative DA neurons was corroborated by their sensitivity to the DA D2 autoreceptor agonist quinpirole (200 μg kg^−1^, intraperitoneally) (Fig. 6q and Supplementary Fig. 31a). After overnight fasting (18 h), animals were implanted with a gut fiber in the proximal small intestine and received intraintestinal microfluidic delivery of either sucrose solution (600 mM, 0.5 ml over 10 min) or saline (0.5 ml over 10 min) while concomitantly recording spiking activity in the VTA. Intestinal sucrose delivery produced a significant increase in the firing rate of DA neurons compared with the preinfusion period, whereas no change in firing rate was observed following saline infusion (Fig. 6r and Supplementary Fig. 31b–e). Since multi-organ implantation of wireless devices can be particularly appealing for functional studies of interoceptive neural circuits, we further established the efficacy of survival surgeries with multisite and multifunctional microelectronic fibers (Supplementary Fig. 32a–h) through assays of feeding and locomotion (Supplementary Note 5 and Supplementary Fig. 33a–h). These experiments highlight the utility of wireless fiber neurotechnology for probing dynamics of circuits spanning multiple organs such as the gut and the brain in awake behaving mice.

Beyond satiety and food intake, gut signals reaching the brain also modulate motivation and reward. Optogenetic stimulation of upper-gut projecting vagal afferents at the level of the brainstem was shown to drive DA-dependent reward behaviors^5^. Motivated by these findings, we applied the gut fibers to test whether the central nervous system functions could be directly controlled from the intestine in behaving mice (Fig. 6s). Using Cre-loxP recombination we bred Phox2b::ChR2 mice, in which ChR2 fused to a fluorescent protein tdTomato was expressed under the Phox2b promoter broadly found in the viscerosensory nodose neurons (Supplementary Fig. 34a)^59^. Subsequently, the Phox2b::ChR2 mice chronically implanted with gut fibers in the duodenum (Supplementary Fig. 34b,c) were subjected to an RTPP behavioral task during which intraluminal vagal stimulation with blue µLEDs (20 Hz, 10-ms pulse width, 0.5 s ON, 1 s OFF) caused a significant preference to the light-paired chamber, compared with their pretest values (Fig. 6t,u). No significant differences in preference for either chamber were observed in control mice lacking ChR2 expression that received identical optical stimulation (Fig. 6v,w). Similarly, intraluminal stimulation in Phox2b::ChR2 mice with control green µLEDs (20 Hz, 10-ms pulse width, 0.5 s ON, 1 s OFF) also did not elicit significant preference for the light-paired chamber as compared with their pretest exploration (Supplementary Fig. 34d,e). These findings illustrate the potential of wireless multifunctional microelectronic fibers to empower studies aimed at unraveling contributions of gut–brain signaling underlying complex motivated behaviors.

## Discussion

The multifunctional and wirelessly capable microelectronic fibers enabled stable bioelectronic interfaces with the brain and gastrointestinal tract in untethered behaving mice. We leveraged the scalability of fiber drawing to produce tens of meters of microscale polymer filaments that can integrate solid-state devices along their surfaces. Coupled with deterministic tunability of fiber mechanics, this approach offers unprecedented design flexibility which is demonstrated by producing stiff yet flexible multifunctional fibers to interface with the brain, and soft compliant fibers to interface with the gut. In doing so, we also overcame outstanding challenges associated with fiber drawing. Thermally drawn fiber-based neural interfaces were limited to tip-localized functionality, used passive features and were incompatible with untethered operation^36,60^. Embedded microelectronics within fiber-based neural probes break their axial redundancy and unlock new stimulation and sensing modes empowered by solid-state devices. By coupling these fibers with NeuroStack modules, we demonstrate their wirelessly programmed control in real-time. Although, unlike a number of elegant fully implantable wireless platforms, the NeuroStack is mounted externally, it enables straightforward deployment using a Bluetooth protocol without specialized antennas. It additionally invites mixing and matching of sensing and modulation capabilities by stacking light-weight customized backend circuits. Thus, the complete platform overcomes several limitations of current wireless bioelectronics technologies by delivering lithography-free scalable fabrication, multifunctionality in a single-step process, stable wireless control without line-of-sight handicap or angular dependency and intuitive operation commensurate with complex behavioral paradigms (Supplementary Note 6 and Supplementary Table 1). Future extensions of multifunctional microelectronic fibers and the NeuroStack module will leverage continued progress in low-power application-specific integrated circuits, miniature batteries and wireless communication protocols to enable closed-loop, fully implantable operation and on-the-fly recharging.

We apply our platform to sense and modulate neural activity in the brain and the gut of freely behaving animals. The brain fibers enable gene delivery, dynamic and chronic opto-electrophysiological monitoring of opsin expression in specific neurons, single neuron recording, sensing of anesthesia-induced brain hypothermia and wireless programmable optical control of reward behavior. The soft gut fibers permit light and nutrient delivery to targeted sites spanning several centimeters in the mouse intestine, enabling modulation of sensory enteroendocrine cells and vagal afferents—capabilities that remained out of reach with previous elegant tools that demonstrated optical modulation in small areas of the stomach fundus or outer colonic wall^18,34,61^. The ability of microelectronic fibers to deliver or sense multiple stimuli in the brain and the gut, and to interface with both organs simultaneously, sets the stage for their application in studies of gut-to-brain signaling. Future innovations in materials and fiber architectures mimicking tissue-level mechanics may extend the application scope of microelectronic fibers to peripheral organs beyond the gut.

We anticipate that with the ever-increasing repertoire of transgenic animals, multifunctional wireless fiber-based tools will provide key insights into the roles of specific cells in bidirectional communication between the peripheral organs and the brain. These tools will empower the study of the enigmatic interoceptive networks in health and disease.

## Methods

### Multifunctional brain fiber draw

The multifunctional brain fibers were produced by thermal drawing from a macroscopic preform which was produced through computer numerical control machining of PC slabs (McMaster, 8574K43). The layer for convergence channels was obtained by milling three square channels of 1.6 × 1.6 mm^2^ with a pitch of 4 mm in a slab of 14.8 mm × 3 mm × 30 cm. A top cover with dimensions of 14.8 × 0.8 mm^2^ fully defined the convergence channels. The subsequent layer had a central channel (3.2 mm × 2 mm × 30 cm) that defined the microfluidic functionality and was flanked by two additional channels (1 mm × 1 mm × 30 cm) on either side for recording electrodes. The tri-layered preform was thermally consolidated at 185 °C for 1 h and drawn into a functional fiber at a size reduction ratio of ~40–50 while simultaneously feeding spools of Ag-Cu and tungsten microwires which serve as interconnects and recording electrodes, respectively.

### Multifunctional gut fiber draw

The preform assembly for soft, multifunctional gut fibers began with molding SEBS pellets (Kraton, G1657) into desired geometrical patterns in a computer numerical control-machined inverse aluminum mold at 200 °C for 12 h under vacuum. The top layer defined the convergence channels (3.6 mm × 3.6 mm × 30 cm) with a pitch size of 4 mm for hosting interconnect microwires. The precursor to the microfluidic channel (2.8 mm × 2 mm × 30 cm) and soft conducting electrodes (2 mm × 2 mm × 30 cm) were incorporated in the bottom layer. The SEBS convergence channels were lined with a U-shaped PC layer that had a wall thickness of 1 mm and channel size of 1.6 × 1.6 mm^2^. Finally, two slabs of carbon-loaded polyethylene (2 mm × 2 mm × 30 cm) were inserted in the bottom layer. The multilayered preform was consolidated at 130 °C for 45 min and subsequently drawn into microscale fibers at a size reduction ratio of ~40–45 on a custom-built draw tower (LabView, v.18.0), while simultaneously feeding three spools of 40 µm Ag-Cu microwires that serve as interconnects.

### Fiber device fabrication and characterization

Fabrication of the implantable brain fiber device began with dissolving away the PC layer on a distal ~1-cm length of the as-drawn fiber in dichloromethane for 2–3 min, which exposed the interconnect and electrode microwires. The microwires were subsequently soldered onto male header pins which were assembled inside a custom three-dimensionally printed casing (5 × 7 × 0.5 mm^3^) and secured using ultraviolet-curable epoxy (NOA 61, Norland Products). Approximately 0.5 cm of the Ag-Cu interconnect was exposed by low-end machining with a razor blade at the distal end of the fiber under an optical microscope, followed by mounting of blue and green μLEDs chips (Cree, TR2227 or SR2130) using reflow soldering (Chip Quik, TS391LT10) or thermally curable silver paste (Epo-Tek, H20E). An insulated stainless-steel ground wire connected to the header pin was soldered onto a ground screw. Connection to the microfluidic channel was established through a T-junction using a polytetrafluoroethylene access tubing. For this purpose, the microfluidic channel on the fiber was first exposed with a razor blade and subsequently the fiber was threaded into the access tubing through a metallic needle. The T-junction was made water-tight by flowing ultraviolet-epoxy at the tubing–polymer junction. The patency of the microfluidic channel was confirmed by flowing a bolus of de-ionized water. Finally, a 12–14-μm layer of vapor-deposited parylene-C (SCS Labcoter2, Parylene deposition system) defined the biofluid barrier layer. The final device assembled in this way had an overall length of ~6–6.5 mm for targeting the VTA. The fabrication of soft gut fiber involved identical steps of exposing interconnects, soldering to input/output (I/O) pins, mounting of μLEDs on the fiber and connecting the microfluidic channel through a T-junction. The final gut fiber had an overall length of ~8.5 cm, which hosted three green and three blue μLEDs on the distal 2-cm length of the fiber at a separation of ~1 cm each. The device was encapsulated in a ~4–6-µm thin layer of parylene-C, followed by encapsulation in a ~100-µm layer of medical grade silicone (MED-6215, Avantor) by inserting the fiber in a polytetrafluoroethylene sacrificial mold. The silicone mixture was filled and thermally cured in the mold along with the fiber at 90–100 °C for 3 h, and subsequently the tubing was cut open to yield the final coated devices for implantation. Details about fiber device characterization and FEM studies appear in the Supplementary methods.

### NeuroStack hardware

The NeuroStack module is composed of a custom printed circuit board (PCB) that carries an MDBT42V wireless microcontroller (with Nordic nRF52832 chip and on-chip PCB antenna) for BLE communication with the central system (nRF52840 DK development kit) which is connected to a base station computer. A male header pin near the edge of the circular board allows the device to connect and disconnect from the implanted probe. Two vertical header pins on the base of the board allow for the attachment and removal of the optional modules. For this study, optional modules offer precise control of optical intensity. To prepare the individual devices and optional modules, components were mounted onto the custom PCBs using reflow soldering and software was loaded using J-link programmers with an Arduino library. The µLEDs integrated with the fibers were driven by either a constant 3.3-V source or a programmable digital-to-analog converter (MAX5510), with a suitable current-limiting series resistor in place to keep the brightness within the desired level. Validation of the device waveforms was done using an oscilloscope to compare measured frequencies and shapes with those specified in the interface. The current consumption patterns were characterized using a Keithley 100B source meter. A further test of battery life was conducted by leaving the system running with the desired stimulation parameters until the output voltage decreased below the optogenetic stimulation threshold. With an 11-mAh-capacity rechargeable lithium battery (MS920SE-FL27E, Seiko Instruments, 9.5-mm diameter coin cell, 3-mm thick, 0.47 g), the brain devices can be operated for up to 1 h on a single charge, while the first version of the gut devices can be operated for up to 30 min which is sufficient for most neuroscience behavioral studies. The modified wireless circuit developed in Supplementary Fig. 28 for performing longer-term feeding studies with gut optogenetics provides up to 2 h of continuous operation on a single charge (45-mAh-capacity rechargeable battery, 4 × 12 × 15 mm^3^, 1.1 g, GM041215, PowerStream). The continuous operational duration of both brain and gut devices can be easily extended to several hours by adopting light-weight, high-capacity batteries (for example, 62-mAh capacity, 3 × 10 × 30 mm^3^, ~1.2 g, GM 01030, PowerStream). For testing the temperature recording function, the probe was placed on a hotplate with a commercial thermocouple and was left to settle for 30 s between each temperature reading. Recordings of the amplifier output voltage and the temperature from the commercial sensor were used to generate the calibration curves. For wireless temperature recording, the transmitted data were collected using a 12-bit analog-to-digital converter of the NRF52 chip and sent over by BLE to a central collection point. To improve the bandwidth and reduce packet overhead, the data were batched at the cost of latency in the recordings. The data received were transferred over a serial interface to the MATLAB (R2019b) program or saved for later analysis.

### NeuroStack user interface

The control software supports the modularity that the hardware presents, provides flexibility to the user in terms of the system preferences, as well as plots and saves the recorded data on the computer. It allows enabling/disabling functionalities as desired and sends real-time stimulation updates to the neuromodulation platform. The system relies on two pieces of software for communication and control of the neuromodulation platform. First is the firmware that is loaded on the neuromodulation board to send the stimulation updates, enable desired functionalities and send data back to the computer if enabled. The peripheral portion of the code was developed in Arduino IDE. The system code was then loaded on the neuromodulation device using Adalink Tool Kit. The central portion of the code was developed using Nordic Software Development Kit and Segger Embedded Studio Software. This portion of the code is responsible for communicating and controlling the peripheral board. The second piece of code essential to the system operation is the graphical user interface, developed in MATLAB (R2019b), which allows the user to select functionalities and send updates to the neuromodulation device in real-time.

### Experiments involving animal subjects

All animal procedures were approved by the MIT Committee on Animal Care and Duke University Institutional Animal Care and Use Committee and carried out in accordance with the National Institutes of Health Guide for the Care and Use of Laboratory Animals. Approximately equal numbers of male and female mice were used. Mice were group housed before surgery and single housed after surgery in cages maintained at 22 °C, 12-h light/dark cycle and 50% humidity with ad libitum access to food and water unless otherwise noted.

### Surgical implantation of microelectronics fibers in the brain

Wild-type mice (C57BL/6) aged 6–8 weeks (Jackson Laboratory, Strain no. 000664) and transgenic DAT::Cre mice (breeding pairs obtained from Jackson Laboratory, Strain no. 006660) aged 8–10 weeks were used for the study, and all surgeries were conducted under aseptic conditions. Mice were anaesthetized with isoflurane gas (0.5–2.5% in O_2_, VET EQUIP) and subsequently positioned in a stereotaxic frame (David Kopf Instruments). After application of ophthalmic ointment to the eyes, a skin incision was made to expose the skull. Lambda and bregma points were used to align the skull with respect to the Mouse Brain Atlas (Franklin and Paxinos). All implantation and injection coordinates were established according to the brain atlas. A single-step injection/implantation was performed in the VTA (coordinates relative to bregma; −3.2 mm anteroposterior; 0.5 mm mediolateral; −4.4 mm dorsoventral). AAV5 viruses carrying *Ef1α::DIO-hChR2-mCherry* and *Ef1α::DIO-mCherry* plasmids were purchased from the University of North Carolina Vector Core at titers of 2 × 10^12^ particles per ml and 3 × 10^12^ particles per ml, respectively. Using a microinjection apparatus (NanoFil Syringe and UMP-3 Syringe Pump, Word Precision Instruments), 1.2 μl of AAV virus was front-loaded into the fiber microfluidic channel. The fiber was lowered into the brain and 600 nl of viral payload was injected at −4.6 mm and −4.2 mm dorsoventral sites at an infusion rate of ~150–300 nl min^−1^. After each injection, the fiber was left undisturbed for further 10 min. The stainless-steel ground screw was affixed to the skull on the cerebellum of the contralateral hemisphere. Finally, the fiber backend connector was fixed to the skull with layers of adhesive (C&B Metabond, Parkell) and dental cement (Jet-Set 4, Lang Dental). Following the surgery, mice were individually housed at 22 °C, 12-h light/dark cycle, with food and water ad libitum. Inclusion criteria for different experiments were as follows: (1) spontaneous electrophysiology—low background noise (<400 µV_pp_); (2) opto-electrophysiology, thermometry—intact µLED as assessed from in vivo and ex vivo *I*–*V* curve; (3) behavior—intact µLED as assessed from in vivo and ex vivo *I*–*V* curve, expression of viral construct as assessed from optically evoked neural activity and/or histology.

### Surgical implantation of fiber probes in the gut

Adult wild-type (Jackson Laboratory, Strain no. 000664), Phox2b::ChR2 (breeding pairs obtained from the Jackson Laboratory, Strain nos. 016233, 012567), Pyy::ChR2 (Pyy::Cre mouse is courtesy of Andrew Leiter, the Jackson Laboratory, Strain no. 012567) or Cck::ChR2 mice (breeding pairs obtained from the Jackson Laboratory, Strain nos. 012706, 012567) (C57BL/6J background) or littermates were anesthetized with isoflurane (1–3% in oxygen). A 1-cm incision was made from the xiphoid process diagonally to the left-mid clavicular line. The peritoneal cavity was accessed, and the stomach extra-corporealized for implantation in wild-type, Phox2b::ChR2 and Cck::ChR2 mice. In these mice, the distal end of the gut fiber was introduced into the duodenum through the pylorus. To access the pylorus, a purse string suture was made in the gastric antrum, between which a small incision was made in the stomach wall. The distal end of the device was threaded into the proximal duodenum. The purse string stitch was then tied to secure the device in the intestine. In Pyy::ChR2 mice, a 1-cm incision was made 3 cm below the xiphoid process to access the cecum. The cecum was extra-corporealized and the distal end of the gut fiber was introduced into the distal ileum through a purse string suture, as in the duodenal surgeries. The purse string suture was tied to secure the device in the ileum. In all surgeries, the remaining length of the device was tunneled to the base of the skull by creating a subdermal pocket. The peritoneum and overlying skin were sutured closed at the abdominal site. The fiber exited the subdermal tunnel at the base of the skull. The skull was etched with a scalpel blade and a thin layer of Metabond cement (Clear L-powder S399 + catalyst, Metabond) was applied. Then, the Metabond layer was similarly etched with a blade, and the backend connector of the fiber was affixed to the skull using dental cement (Stoelting, no. 51458). Mice recovered for at least 5 d, during which they were fed wet mash and received appropriate postoperative care. Inclusion criteria at the end of each study were that the fibers were appropriately secured in the proximal small intestine and that the µLEDs were operational.

### In vivo electrophysiology and opto-electrophysiology

Implanted fibers were connected to the RZ5D electrophysiology system through a PZ2-32 head stage (Tucker Davis Technologies). Following data acquisition, electrophysiological signal was digitized with 50-kHz sampling frequency and filtered in the frequency range 0.3–5 kHz. Subsequent signal processing and analysis were done in MATLAB (R2019b). Spiking activity was detected using threshold detection, with a threshold of 5 s.d. from the mean of the signals. A downtime of 2 ms was employed to reject double detections. Principle component analysis and Gaussian mixture model clustering were used to perform spike classification and clustering (full and independent covariance matrices). L-ratio and the isolation distance of the classified clusters were used to assess the quality of the clustered data. For opto-electrophysiology experiments, optogenetic stimulation pulses were delivered via integrated μLEDs on the fiber with rise/fall time between 1 and 15 ms to minimize capacitively coupled artifacts. Stimulation was delivered in 1-s stimulation epochs separated by 4-s rest epochs at a frequency of 10 Hz from the RZ5D acquisition system with a custom designed connector.

### In vivo impedance spectroscopy

To assess the in vivo stability of the recording electrodes, we performed impedance spectroscopy on these electrodes versus the ground screw in the VTA of wild-type implanted mice for up to 6 months (*n* = 3 mice) with a portable BioLogic VMP3 potentiostat.

### In vivo brain temperature measurements

Wireless intracranial temperature measurements were performed in an open-field arena. Wild-type mice (*n* = 6) implanted in the VTA with the precalibrated microelectronic fibers were coupled to the NeuroStack module and allowed to explore an open-field arena (30 × 30 cm^2^) as they received wireless photostimulation (20 Hz, 10-ms pulse width) with blue μLED for 200 s. Anesthesia-induced brain hypothermia was quantified following an intraperitoneal injection of an anesthetic drug mixture of ketamine-xylazine (9:1 dilution) at 30-mg kg^−1^ and 60-mg kg^−1^ dosages in wild-type mice implanted with precalibrated microelectronic fibers in the VTA (*n* = 3 for each dosage). The current response from the fiber thermal sensor was measured with a potentiostat (Solartron, 1280C) in a two-electrode configuration until the animal gained consciousness and began freely ambulating in the homecage.

### In vivo gut temperature measurements

Wireless gut temperature measurements were performed in a clean homecage with food and water removed. A wild-type mouse was chronically implanted with a gut fiber in the duodenum as described above. Temperature was continually recorded while the animal received wireless photostimulation (20 Hz, 10-ms pulse width) with the implanted blue μLED for 10 min in the homecage.

### In vivo gut optofluidic modulation with simultaneous vagal cuff electrophysiology

Whole nerve recordings were performed in Cck::ChR2 mice as described previously^40^. A gut fiber with two connected tubes for PBS perfusion and stimulant delivery was surgically inserted through the stomach wall into the duodenum. A perfusion exit incision was made at the ligament of Treitz for the small intestine. To control for volume pressure and to act as a within-subject baseline, PBS was constantly perfused through the isolated intestinal region at ~400 μl min^−1^. Stimulation conditions were applied after recording 2 min of baseline activity. During nutrient stimulation conditions, PBS perfusion was continuous and 200 μl of stimulant was perfused over 1 min using a syringe pump (Fusion 200, Chemyx). The 1-min infusions of each compound were separated by at least 6 min, or the return to baseline firing rate, whichever came first. Sucrose (300 mM) was used as the nutrient, as it is known to stimulate vagal firing rate. Blue (*λ* = 470 nm, 20 Hz, 30.3 mW mm^−2^, 10-ms pulse width) or green light (*λ* = 527 nm, 20 Hz, 45.6 mW mm^−2^, 10-ms pulse width) was delivered via fiber µLEDs concomitant with the sucrose infusion through the microfluidic channel. Extracellular voltage was recorded as previously described^40^. The raw data were analyzed using SpikeTailor, a custom MATLAB (R2019b) software script^40^. Spikes were detected using a threshold of 2 s.d. above the noise floor, determined by the root mean squared noise. The firing rate was calculated using a Gaussian kernel smoothing algorithm in 200-ms bins^40^.

### In vivo intestinal nutrient delivery with simultaneous brain electrophysiology

C57BL/6J mice were chronically implanted with a brain fiber in the VTA and allowed to recover for 1 week. Subsequently, electrophysiological recordings were performed and analyzed as detailed in the ‘In vivo electrophysiology’ section. The identity of putative DA neurons was assessed by their characteristic inhibitory response to dopamine D2 receptor agonist quinpirole (200–300 µg kg^−1^, intraperitoneally). Firing rate was computed for each recorded unit based on the peristimulus time histogram with a window size of 1 s (moving average Gaussian with a standard deviation of 200 ms). Animals that did not yield successful recordings from DA neurons (background noise > 400 µV_pp_ or insensitive to quinpirole administration) were excluded from the experiment. Subsequently, the animals were fasted overnight for 18 h and underwent a second surgery for intraduodenal implantation of a gut fiber. Sucrose (0.6 M, 0.5 ml over 10 min) or saline infusions (0.5 ml over 10 min) were performed with a programmable infusion pump (New Era Pump Systems) while concomitantly recording VTA activity, which also extended up to 10 min after the end of the infusion session. Average firing rate was computed for each putative DA unit over a 120-s recording window pre- and postinfusion, based on the mean interstimulus interval at a 40-ms bin size.

### RTPP assays

Behavioral tests were performed by an investigator with knowledge of the identity of the experimental groups versus control groups.

#### Brain

DAT::Cre mice implanted with multifunctional microelectronics fibers and injected with viral vectors in the VTA were handled and acclimated to the investigator for 2 d before the behavioral test for 10 min each (*n* = 8–10 per group). On the third day, animals were acclimated to the connection of the NeuroStack module and allowed to explore their homecage for 15 min. On the following day (pretest day), the NeuroStack module-carrying mice were allowed to freely explore an unbiased two-compartment chamber (60 × 30 × 30 cm^3^) for 30 min while being video recorded. The time spent by an animal in each chamber was calculated using a behavioral software (Ethovision XT, Noldus). Mice that showed >70% preference to a chamber during pretest were eliminated from the subsequent analyses. On the day of the test, the less-preferred chamber for each animal was coupled to the wireless photostimulation condition by controlling the NeuroStack from a base computer positioned ~5 m away. The live video feed from a recording camera provided input on the animal location and the investigator controlled the status of the stimulation condition in real-time. Three different photostimulation conditions were tested using the above procedure: (1) stimulation ON (470 nm, 25 Hz, 10-ms pulse, 1 s ON, 2 s OFF) versus OFF (no stimulation); (2) phasic bursting (470 nm, 40 Hz, 5-ms pulse, 0.5 s ON, 4 s OFF) versus tonic stimulation (5 Hz, 5-ms pulse, ON); (3) blue (470 nm, 25 Hz, 10-ms pulse, 1 s ON, 2 s OFF) versus green stimulation (527 nm, 25 Hz, 10-ms pulse, 1 s ON, 2 s OFF).

#### Gut

Phox2b::ChR2 mice and their negative genotype littermates were implanted with the gut fiber in the duodenum. Mice were acclimated to investigator handling and connection to the NeuroStack module akin to the brain experiments. On the pretest day, the NeuroStack module was attached the I/O pins of the implanted fiber and mice were allowed to freely explore an unbiased chamber (Techniplast Greenline IVC cage for mice) for 20 min. Animal activity was determined by beams crossed in the *x* and *y* planes and was collected with a 100-Hz scan rate using the TSE PhenoMaster (software v.7.3.3). On test day, the less-preferred chamber for each animal was coupled to wireless photostimulation by controlling the wireless module from a base computer. The live activity feed provided input on mouse location and the investigator wirelessly controlled the stimulation status in real-time. There were three different conditions: (1) the experimental group was Phox2b::ChR2 mice receiving blue-light stimulation (470 nm, 20 Hz, 10-ms pulse, ON) versus no stimulation (OFF); (2) the µLED control group was Phox2b::ChR2 mice receiving green-light stimulation (527 nm, 20 Hz, 10-ms pulse, ON) versus no stimulation (OFF); (3) the genetic control group was negative genotype littermates receiving blue-light stimulation (20 Hz, 10-ms pulse, ON) versus no stimulation (OFF).

### Modulation of feeding behavior

For duodenal studies, Cck::ChR2 mice or negative controls were implanted with the soft gut fiber in the proximal duodenum. Mice were acclimated to the investigator handling and connection to the NeuroStack module. Mice were food-deprived overnight (18 h) before connection to the NeuroStack module and receiving 30 min of wireless optical stimulation (20 Hz, 10-ms pulse). After 30 min of stimulation, mice were disconnected from the NeuroStack and given access to standard chow pellets (Purina 5001). Chow intake was measured each hour for 3 h. Mice had ad libitum access to water for the duration of the food restriction and testing. Each mouse received blue-light and green-light stimulation, randomized per condition. At least 48 h separated each test day. For ileal studies Pyy::ChR2 mice or negative controls were implanted with the soft gut fiber in the ileum. Mice were acclimated to experimenter handling and connection to the NeuroStack module. Mice were acclimated to Ensure (30%) solution for 6 h following surgical recovery and at least 48 h before the first experimental session. Mice were food-deprived overnight (18 h) before connection to the NeuroStack module. Mice received 1 h of wireless optical stimulation (20 Hz, 10-ms pulse), which began 10 min before getting access to the Ensure solution. Ensure (30%) was loaded into 5-ml serological pipettes that were fashioned as sippers. Mice had access to the solution for 1 h and intake was measured every 5 min. Mice did not have access to food or water during the test sessions. Each mouse received blue-light and green-light simulation, randomized per condition. At least 48 h separated each test day. The feeding studies utilized the multiple independently addressable stimulation channels on the gut fiber. As such, these experiments were designed with within-subject controls. While the baseline food intake was lower in the control cohort of mice lacking ChR2, we attribute this to differences in individual body weight^62^ and time of year of testing^63^, which were unaccounted for in the within-subject design^64^ of these studies.

### Locomotor assays of brain and gut implanted mice

To test whether brain, gut or gut–brain dual implants coupled to the NeuroStack impacted locomotion, we evaluated locomotor behavior over 20 min. Open-field tests on naïve mice (un-operated) and implanted mice carrying NeuroStack were conducted in an open chamber (60 × 30 × 30 cm^3^) for brain implantations and in the homecage for gut implantations and gut–brain dual implantations over 20 min. Locomotor activity was recorded as described in the RTPP assay. The mouse position, distance traveled and speed were calculated.

### Food and water intake of gut and gut–brain implanted mice

Animals were individually housed in a custom-built PhenoMaster behavioral phenotyping system (TSE Systems). The PhenoMaster was programmed (software v.7.3.3) to automatically maintain a light cycle (3:00 lights on; 15:00 lights off), temperature (22 °C) and humidity (50%). Animals were provided with standard mouse chow (Purina, 5001) and reverse osmosis water ad libitum. Food hoppers and water bottles were attached to weight sensors (TSE), which were automatically sampled every 5 s to the nearest 0.01 g. For drinking measurements, a 10-s smoothing interval was used. For food and water weight measurements, a 15-s smoothing interval with a 15-g threshold was used. Intake was measured every 5 s. Data were corrected for minor fluctuations by only permitting a monotonically increasing function for both food and water intake: values that represented negative food intake were replaced by the most recent value. For stability of intake measurements, the mean food intake and water intake were calculated for 2 consecutive days for each individual mouse.

### Immunohistochemical evaluation of foreign body response

Wild-type mice (*n* = 5 per group) bilaterally implanted in the VTA with microelectronics brain fibers or commercial silica waveguides (300 µm, FT300UMT Thorlabs) were anesthetized with isoflurane, injected intraperitoneally with Fatal-Plus (100 mg kg^−1^), and transcardially perfused with 50 ml of ice-cold PBS followed by 50 ml of ice-cold 4% paraformaldehyde (PFA) in PBS. The devices were carefully explanted and the brains were removed and additionally fixed in 4% PFA in PBS for 24 h at 4 °C, then stored in PBS afterwards. Coronal slices (50-µm thickness) were prepared using a vibratome (Leica, VT1000S) and a razor blade (Electron Microscopy Sciences, 72002) in ice-cold PBS. The slices were then stored in PBS at 4 °C in the dark until staining. Slices were permeabilized with 0.3% v/v Triton X-100 and blocked with 2.5% donkey serum in PBS for 30 min. Slices were incubated overnight at 4 °C in a solution of 2.5% donkey serum in PBS and a primary antibody (Iba1: Goat anti-Iba1, ab107159 Abcam, 1:500 dilution; GFAP: Goat anti-GFAP, ab53554 Abcam, 1:1,000 dilution). Following incubation, slices were washed three times with PBS. The slices were then incubated with a secondary antibody (Donkey anti-Goat Alexa Fluor 488, A11055, 1:1,000, Thermofisher) for 2 h at room temperature on a shaker followed by an additional three washes with PBS. Slices were then incubated with DAPI (4′6-diamidino-2-phenylindole) (1:50,000) for another 20 min, and washed three times with PBS. Fluoromount-G (SouthernBiotech) was used for mounting slices onto glass microscope slides. A laser scanning confocal microscope (Fluoview FV1000, Olympus) was used for imaging with ×20 objectives, with z-stack images across the slice thickness. Region of interest was chosen based on the implant location, imaging the immune response around the implant trace. FIJI (ImageJ 1.53g) was used to quantify the immune response as total integrated fluorescence intensity normalized to the image acquisition area.

### Hematoxylin and eosin stain evaluation of foreign body response

Duodenal tissue from wild-type mice naïve to (*n* = 4) and implanted with (*n* = 4) the duodenal implant was sliced and evaluated for hematoxylin and eosin by the Duke Pathology Histology Lab. The villus height and crypt depth were measured using ImageJ 2 (1.5.3). For each mouse, the villus height and crypt depth were calculated as the average of ten villi or crypts.

### Statistics and reproducibility

OriginPro 2021b, JMP Pro 15 or JMP Pro 16 software was used to assess the statistical significance of all comparison studies in this work. Power analyses for determining sample sizes for immunohistochemistry and behavior tests were not performed; instead, the group sizes were chosen based on previous research conducted in the same brain circuit or intestinal region. This enabled direct comparison of our results with the earlier work. In the statistical analysis of fiber characterization, one-way analysis of variation (ANOVA) followed by Tukey’s post hoc comparison test was used, with thresholds of *P < 0.05, **P < 0.01, ***P < 0.001. For the comparisons between two groups in immunohistochemistry analyses, behavior assays and hypothermia effect quantification, *t*-test was used, and significance threshold was placed at *P < 0.05, **P < 0.01, ***P < 0.001. For all parametric tests, normality was determined using the Shapiro–Wilk method. Homogeneity of variances was determined using Levene’s test wherever appropriate. All shaded areas and error bars represent standard deviation; data are presented as mean ± s.d. Confocal micrographs of brain tissue were collected on at least three slices per brain to confirm reproducibility of results. Appropriate fluorophores were evaluated in each opsin-expressing mouse included in the studies. Micrographs of gut tissue were collected on at least three tissue samples per animal to confirm reproducibility of results. Micrographs of fiber cross-section were collected from five randomly selected regions of the draw to confirm reproducibility. Other digital photographs such as those of the preform, fiber bundles, final devices and implanted animals were collected once to serve as representative examples.

### Reporting summary

Further information on research design is available in the Nature Portfolio Reporting Summary linked to this article.

## Online content

Any methods, additional references, Nature Portfolio reporting summaries, source data, extended data, supplementary information, acknowledgements, peer review information; details of author contributions and competing interests; and statements of data and code availability are available at 10.1038/s41587-023-01833-5.

### Supplementary information

Supplementary Information Supplementary Methods, Notes 1–6, Figs. 1–34, Table 1, captions to Videos 1–7 and References.

Reporting Summary

Supplementary Video 1 A representative video of the scalable fiber fabrication process using thermal drawing showing convergence of metal interconnects into a preform.

Supplementary Video 2 Independently addressable blue and green µLEDs and programmable control of stimulation frequency in a brain fiber.

Supplementary Video 3 Independently addressable blue and green µLEDs and programmable control of stimulation frequency in a gut fiber.

Supplementary Video 4 Independently addressable multicolor µLEDs in a brain and gut fiber highlighting the capability of simultaneously controlling multiple devices implanted in distinct anatomical regions.

Supplementary Video 5 A representative video of a mouse implanted with a microelectronic brain fiber in the ventral tegmental area and carrying NeuroStack module in an open-field chamber.

Supplementary Video 6 A representative video of a pair of Phox2b::ChR2 mice implanted with a gut fiber carrying NeuroStack module ambulating in the homecage.

Supplementary Video 7 A representative video of a Pyy::ChR2 mouse chronically implanted with a microelectronic gut fiber in the ileum and connected to NeuroStack drinking high-fat solution in the homecage.

Supplementary Data 1 Statistical source data.

Supplementary Data 3 Statistical source data.

Supplementary Data 4 Statistical source data.

Supplementary Data 5 Statistical source data.

Supplementary Data 6 Statistical source data.

Supplementary Data 7 Statistical source data.

Supplementary Data 8 Statistical source data.

Supplementary Data 9 Statistical source data.

Supplementary Data 10 Statistical source data.

Supplementary Data 11 Statistical source data.

Supplementary Data 14 Statistical source data.

Supplementary Data 15 Statistical source data.

Supplementary Data 16 Statistical source data.

Supplementary Data 17 Statistical source data.

Supplementary Data 21 Statistical source data.

Supplementary Data 22 Statistical source data.

Supplementary Data 23 Statistical source data.

Supplementary Data 24 Statistical source data.

Supplementary Data 25 Statistical source data.

Supplementary Data 26 Statistical source data.

Supplementary Data 30 Statistical source data.

Supplementary Data 32 Statistical source data.

Supplementary Data 33 Statistical source data.

Supplementary Data 34 Statistical source data.

### Source data

Source Data Fig. 2 Statistical source data.

Source Data Fig. 3 Statistical source data.

Source Data Fig. 4 Statistical source data.

Source Data Fig. 5 Statistical source data.

Source Data Fig. 6 Statistical source data.

## Data Availability

Source data are provided with this paper.

## Code availability

The custom software script, microcontroller firmware code and NRF52840 Development Kit firmware code used in this work are available at: https://github.com/HarrisonAllen/Wireless-Controllers-for-Microelectronic-Fibers.
