# Supplementary material for: Multifunctional microelectronic fibers enable wireless modulation of gut and brain neural circuits
Source: Nat Biotechnol. 2023 Jun 22;42(6):892–904. doi: 10.1038/s41587-023-01833-5 (PMC11180606; doi:10.1038/s41587-023-01833-5)
Supplement: Supplementary file 1 — Supplementary Methods, Notes 1–6, Figs. 1–34, Table 1, captions to Videos 1–7 and References. [file 41587_2023_1833_MOESM1_ESM.pdf]

nature biotechnology

Article

https://doi.org/10.1038/s41587-023-01833-5

Multifunctional microelectronic fibers enable wireless modulation of gut and brain neural circuits

In the format provided by the authors and unedited

Multifunctional microelectronic fibers enable wireless modulation of gut and brain neural circuits

Atharva Sahasrabudhe et al.

Supplementary Information

Table of Contents

Supplementary Methods ... **4**

Supplemental Note 1: Interconnects to support microelectronics in thermally drawn fibers ... **7**

Supplemental Note 2: Incorporation of semiconductor devices during fiber drawing ... **7**

Supplemental Note 3: Comparison of NeuroStack module with battery-free technologies ... **8**

Supplemental Note 4: Transient voltage pulse shaping removes capacitive artifacts ... **9**

Supplemental Note 5: Multisite, multifunctional fiber device enables simultaneous gut-brain implantation ... **10**

Supplemental Note 6: State-of-the-art in multifunctional bioelectronic interfaces ... **12**

Figure S1. Thermal drawing data for the brain fibers ... **13**

Figure S2. Process flow for fiber connectorization ... **14**

Figure S3. Integration of microelectronic devices at the preform stage permits *in-situ* electrical bonding and packaging in a multifunctional fiber ... **15**

Figure S4. Electrical and optical characterization of the brain fibers ... **17**

Figure S5. Optical FEM simulations of brain fibers in the brain tissue ... **18**

Figure S6. Thermal FEM simulation of brain fibers in the brain tissue ... **19**

Figure S7. Characterization of thermal sensing modality of the brain fiber ... **20**

Figure S8. Mechanical FEM simulation of the brain fiber in the brain tissue ... **21**

Figure S9. Optical characterization of soft gut fiber ... **22**

Figure S10. Optical FEM simulations of gut fiber in the intestinal lumen ... **23**

Figure S11. Thermal FEM simulation of the gut fiber in the small intestine lumen ... **24**

Figure S12. Circuit layout of NeuroStack primary module, graphical user interface (GUI), and power consumption profile across different modalities ... **26**

Figure S13. Extended data for figure 5d ... **27**

Figure S14. *In-vitro* quantification of capacitively coupled artifacts in brain fiber ... **28**

Figure S15. *In-vivo* quantification of capacitively coupled artifacts in brain fiber ... **30**

Figure S16. Chronic recordings of optically evoked electrical activity in mice transduced with ChR2 in dopaminergic (DA) neurons ... **32**

Figure S17. *In-vivo* stability of recording electrodes over 6 months 33

Figure S18. Extended data for figure 5e 34

Figure S19. Chronic recording of single unit activity in the VTA at week-2 following implantation 35

Figure S20. Chronic recording of single unit activity in the VTA at week-4 following implantation 36

Figure S21. Immunohistochemical evaluation of biocompatibility of chronically implanted brain fiber 37

Figure S22. Temperature sensing modality in the fiber detects anesthesia-induced brain hypothermia in a dose dependent manner 39

Figure S23. Wireless programmable optical stimulation of DA neurons in the VTA progressively imparts place preference 40

Figure S24. Control behavior experiments and characterization of explanted devices 42

Figure S25. Optofluidic modality of gut fiber, control vagal recordings, and intraluminal gut temperature recording 43

Figure S26. Feeding and locomotor behavior of mice chronically implanted with soft gut fiber in the proximal duodenum 44

Figure S27. Histological assessment of biocompatibility of chronically implanted gut fibers 45

Figure S28. Visualization of the sparsely distributed cholecystokinin (Cck+) neuropod cells and the gut fiber during operation in the duodenum 46

Figure S29. Modified wireless module that enables prolonged operation during feeding behavior 47

Figure S30. Visualization of the sparsely distributed Peptide YY (Pyy+) neuropod cells in gut epithelium and gut fiber in operation in the ileum 48

Figure S31. Electrophysiological recordings in the VTA via an implanted brain fiber pre- and post- sucrose infusion into the gut lumen via an implanted gut fiber 49

Figure S32. Thermally drawn stretchable interconnects enable multisite devices 50

Figure S33. Compatibility of multisite devices with long-term implantation 51

Figure S34. Visualization of Phox2b+ vagal afferents in the duodenum and place-preference behavior controls 53

Table S1: Comparison of state-of-the-art wireless multifunctional bioelectronic interfaces. 54

Video V1: A representative video of the scalable fiber fabrication process using thermal drawing showing convergence of metal interconnects into a preform. 55

Video V2: Independently addressable blue and green μLEDs and programmable control of stimulation frequency in a brain fiber.......... 55

Video V3: Independently addressable blue and green μLEDs and programmable control of stimulation frequency in a gut fiber. 55

Video V4: Independently addressable multicolor μLEDs in a brain and gut fiber highlighting the capability of simultaneously controlling multiple devices implanted in distinct anatomical regions. 55

Video V5: A representative video of a mouse implanted with a microelectronic brain fiber in the ventral tegmental area and carrying NeuroStack module in an open field chamber. 55

Video V6: A representative video of a pair of Phox2b::ChR2 mice implanted with a gut fiber carrying NeuroStack module ambulating in the home cage. 55

Video V7: A representative video of a Pyy::ChR2 mouse chronically implanted with a microelectronic gut fiber in the ileum and connected to NeuroStack drinking high fat solution in the home cage. 55

References 56

Supplementary Methods

Fiber characterization. For cross-sectional imaging of the fibers, samples from different sections of the draw (n=5 randomly selected sections) were cold mounted into epoxy resin and polished on an automated grinding machine with series of sand papers of decreasing grain sizes (25N force, 300 rpm, 2 min for each sand paper with washing step in between). The polished epoxy blocks were imaged on a Nikon MA200 microscope. The electrode impedance for brain fiber (n=3 devices) was measured with a precision LCR meter (HP4284A, Agilent Technologies) with a sinusoidal input (10 mV bias, 20 Hz–10 kHz). The optical characterization of fiber integrated μLEDs (n=6 devices) was performed by powering them at different bias voltages and recording the light output with a photodetector (S121C, 400–1100 nm, 500 mW, Thorlabs) attached to a power meter (PM100D, Thorlabs). The I-V response of μLEDs was recorded with a potentiostat in a two-electrode configuration (Solartron 1280C and CorrWare 3.5i). The bending stiffness was measured with a dynamic mechanical analyzer (Q800, TA Instruments and Advantage v5.5). Different samples (n=3 fibers) of 1.2 cm lengths were mounted in a single cantilever clamp and tested with a frequency sweep (0.1–10 Hz) under controlled displacement of 20 μm at 37 °C. The cyclic bending tests (n=3 fibers) for soft gut fibers was performed with mechanical testing machine (Z2.5 with testXpert Ill Vl.11, Zwick/Roell) over 10^4 cycles, while recording the light output from μLEDs after every decade. The diode based thermal sensors were calibrated against a commercial thermocouple (NeuLog, NUL-203) by equilibrating the fiber and thermocouple in a hot water-bath or a hot plate at different temperatures and recording the current response from the diode at 2.2 V forward bias (n=4 devices). A fitted linear regression between the diode current and equilibrium thermocouple temperature yielded the standard curve for the sensor. The microfluidic capability of brain fibers (n=4) was evaluated by connecting the probes to an injection syringe (NanoFil) that was driven by an infusion pump (UMP-3, World Precision Instruments) and flowing a DI water bolus at different injection speeds. The injection output was measured by weight and the injection rate was obtained upon dividing the injected volume by the time required to complete the injection. Identical procedure was employed for microfluidic characterization of gut fibers, by injecting a 0.2 ml bolus with a programmable pump (New Era Pump Systems) at rates commensurate with intraintestinal infusions.

Optical simulations. Finite-elements simulation implemented in COMSOL Multiphysics (Version 5.6) were used to model the optical field of the tissues. For absorbing-scattering media like biological tissues, the light transport equation was employed, where the light fluence rate at a given location in steady state $\Phi(r)$ obeys $D\nabla^2\Phi(r) - \mu_a\Phi(r) = 0$, where $D = \frac{1}{3}(\mu_a + \mu_s{}')$ is the diffusion constant, μ_a is the absorption coefficient of the medium, and $\mu_s{}'$ is the reduced scattering coefficient. The brain is modeled as a homogeneous medium [μ_a: 3.67 cm^{-1} (at 475 nm), 3.84 cm^{-1} (at 532 nm), $\mu_s{}'$: 51.15 cm-1 (at 475 nm), 46.3 cm-1 (at 532 nm)] with dimensions set such that the radiative power decays to zero before reaching to the boundary of the brain. To model the power of a µLED, an omnidirectional plane source is inserted at the center of the brain. The power of the source is defined to be two times the actual power to account for the emission directivity of the µLED. The intestine tissue [μ_a: 26.3 cm^{-1} (at 475 nm), $\mu_s{}'$: 33.25 cm^{-1} (at 475 nm)] is modeled as two coaxial cylinders filled with chyme, with the outer one corresponding to the serosal membrane (r = 1.8 mm) and the inner one corresponding to the mucosal membrane (r = 1.5 mm). The gut fiber is placed at the center of the coaxial structure and the region of the gut wall is employed for the light transport equation. The µLED emission profile is approximated by a power cosine function. Light emitted from the center of the µLED is propagated to the inner surface of the intestine to determine the fluence rate at the inner wall, which is used as a boundary source to calculate the power distribution in the intestine region. An optogenetic threshold of 0.1 mW/mm^2 is used to estimate the optical penetration depth and volume in all simulations[1–3].

Thermal simulations. Finite-element simulations via COMSOL Multiphysics (Version 5.6) were used to determine the temperature profile of tissues during µLED operation. The transient heat transport equation in a biological tissue can be expressed as $\rho C_p \frac{\partial T}{\partial t} + \rho C_p \boldsymbol{u} \cdot \nabla T - k\nabla^2 T = Q + Q_{bio}$, where ρ, k C_p, and $\boldsymbol{u}$ are the density, thermal conductivity, heat capacity, and fluid velocity, respectively. Q_{bio} represents a volumetric bioheat source term that can be further expressed as $Q_{bio} = \rho_b C_{p,b}\omega_b(T_b - T_2) + Q_{met}$. T_b (310.15 K), $C_{p,b}$ (36000 J/kg.K), ω_b(0.008 s^{-1}), ρ_b (1057 kg/m^3), Q_{met} (9132 W/m^3) are the arterial blood temperature, specific heat of blood, blood perfusion rate, blood density, and the metabolic heat source, respectively. In addition to the metabolic heat source, there are two other sources of heat that originate from the µLED. The first heating source is due to the absorbance of optical power in the tissue, and the

second is direct heat dissipation associated with μLED inefficiency. For modeling heat transport in the brain, the first term was introduced through coupling of the heat transfer module with the radiation in absorbing-scattering medium module in COMSOL, and the second term was introduced by defining a surface heat source at the μLED. The temperature profile in the gut was obtained without considering the optical absorption term due to the high light transmission through the chyme region. In addition, heat generated by blood perfusion was not considered in the intestinal tissue.

Mechanical simulations. The finite element modelling software, ABAQUS 2019 (Dassault systems), was used for simulating the stress distribution profiles in different fibers and the fiber displacement during brain tissue micromotion. The components that make up the fiber, such as the polymer cladding, and metal microwires, are represented with an 8-node 3D brick element in a HEX shape (C3D8H) with the corresponding mechanical properties. The biological tissue is represented as a 3D brick element with a TET shape (C3D10H). To simulate the relative motion of the brain fiber in the brain tissue, the tissue at the fiber tip was displaced by a fixed amplitude (0.01-0.1 mm) in a direction orthogonal to the fiber axis, while the other fiber end was held fixed to mimic skull-fixation in an implanted animal. A friction coefficient of 0.3 established contact interaction between the tissue and the fiber. The displacement of the fiber tip was quantified as a function of tissue micromotion. To simulate the stress distribution profiles in the gut fiber during surgical implantation process, the fiber was curved at different radii of curvature and the % strain in the copper interconnects was evaluated. To simulate the stress distribution profile as a function of polymer cladding stiffness, the gut fiber was held fixed at one while the other end was set free to deflect which mimics the implanted probe that is sutured to the stomach wall but is free to deflect in the gut lumen. Upon application of a small point load *F* at the free end, a deflection in the fiber was produced and the corresponding misses stress profile in various fiber components was captured.

Supplemental Note 1: Interconnects to support microelectronics in thermally drawn fibers

Incorporation of microelectronic chips in polymer fibers requires introduction of conducting interconnects that can robustly bond with the solid-state devices and power them without substantial resistive heating. However, achieving this with thermal drawing process is a challenge, as the technique imposes restrictions on the thermomechanical properties of constituent materials in a fiber that should have similar viscosity at the drawing temperature[4]. As such, possible material candidates that are compatible with fiber drawing and can function as interconnects are: 1) conducting composites, and 2) low melting point (T_m) metals. However, common conducting composites have conductivity that is 7-8 orders of magnitude lower than that of metals, which makes them unsuitable for use as interconnects[5,6]. The low T_m metals have sufficient conductivity but are more likely to exhibit fluid instabilities and undergo capillary breakup during fiber drawing[7,8]. To overcome these challenges, we leverage recently developed approach of convergence drawing[9,10]. During convergence drawing microwires of any material, irrespective of its thermomechanical properties, are fed into shrinking channels of a polymer preform. As the diameter of the channel approaches that of the microwire, the former is incorporated into the polymer cladding and is drawn by a combined effect of shear forces exerted by the viscous polymer and the pulling force of the capstan. We adapt this method to introduce microscale interconnects of Ag-Cu (40 μm) into multifunctional fibers (Video V1). To facilitate exposure of metal interconnects, the convergence process was carefully optimized such that the Ag-Cu microwires were positioned at a distance ≥15 μm from the top fiber surface.

Supplemental Note 2: Incorporation of semiconductor devices during fiber drawing

We demonstrate a self-assembly mechanism that enables robust *in-situ* electrical bonding of μLEDs as well as packages them in a layer of insulating polymer cladding at scale[10]. For this purpose, we assembled a multilayer poly(carbonate) (PC) preform (Fig. S3a) that consisted of two convergence layers (7 mm × 2.4 mm × 25 cm), a thin spacer layer (7 mm × 0.2 mm × 25 cm) and a central chip-containing layer (7 mm × 1.2 mm × 25 cm). Multiple microscale pockets (20 pockets, 300 μm × 300 μm × 50 μm) were machined within the central chip layer that accommodated μLEDs (UT170, Cree). The μLEDs have metal bonding pads on either side of the $In_xGa_{1-x}N$ emissive layer, which facilitates *in-situ* interconnect bonding during the draw. The preform was consolidated along with the μLED chips at 185 ºC for 1 hr. We utilized a 2-step

drawing process to obtain the resultant multifunctional fibers. In Step-1 (Fig. S3b) the preform was drawn along with the convergence of 40 µm Ag-Cu microwires that served as interconnects. To engineer *in-situ* bonding between chips and interconnects, we used a draw-down ratio of 25-30 that was higher than the predetermined value of 20, resulting in an "over-convergence" state. At this condition the microwires cut through the thin polymer spacer layer (~10 µm) and bond to the metal contact pads of µLEDs (Fig. S3b, Inset). The µLEDs themselves flow along with the viscous polymer due to shear forces during the draw. In Step-2 of the drawing process, we incorporated the microscale fiber produced in Step-1 through convergence inside an elastomeric cyclic olefin copolymer (ECOC) preform that also hosted a channel for microfluidic delivery and tungsten recording microelectrodes. The use of low T_g (90 °C) ECOC preform for Step-2 of the drawing process ensured that the converging chip-embedded PC fibers did not thermally deform during the draw (T_g of PC = 150 °C). Moreover, ECOC also provided moisture barrier properties serving as a packaging layer. Figure S3c shows a digital micrograph of a chip-loaded PC preform prior to thermal consolidation. Figure S3d shows the set-up for step-2 of the drawing process. The cross-sectional geometries of fibers from Steps 1 and 2 are conserved (Fig. S3 e-f). All integrated µLEDs across several meters of fibers from Steps 1 and 2 were functional and electrically connected (Fig. 3 g-i). Finally, the current-voltage (I-V, Fig. S3j) and light-output characterization (Fig. S3k) of several sections of as-drawn fibers confirmed the robustness of *in-situ* electrical bonding. Thus, like any other fiber functionality, microelectronic components are also amenable to scalable monolithic integration inside a multifunctional polymer fiber.

Supplemental Note 3: Comparison of NeuroStack module with battery-free technologies

Battery-free approaches for powering bioelectronic devices can be classified into radiative far-field and non-radiative near-field power transmission. The far-field approach leverages radio waves in the range of 400 MHz to 3 GHz that are straightforward to generate and provide relatively long transmission distances (~1 m). However, these frequencies also interfere with other telecommunication technologies such as WiFi (~2-5 GHz) and GPS (~1-2 GHz) which makes far-field radio waves susceptible to electromagnetic interference with these background signals. Moreover, the high frequency radio waves can also interfere with themselves upon reflection or scattering from metal objects. On the other hand, the non-radiative near-field power delivery relies on induction of electromotive force in an electrical conductor due to a changing

external magnetic field. In this approach the receiver coil is magnetically coupled to the transmitter coil at a target resonance frequency (100 kHz-100 MHz). While the near-field coupling is less susceptible to background interferences and reflections, its power harvesting efficiency varies as a cosine of the angle between the transmitting and receiving coil making this approach susceptible to angular variations during animal behavior. Moreover, both these approaches require design and tuning of transfer coils or resonant cavities for power transmission around behavioral arenas and large radiofrequency sources, which need optimization depending on the geometry and complexity of behavioral assays. This restricts the size of the behavior arena and limits long range operation in large complex spaces. The coupling of electrical power in battery-free devices is also restricted by volumetric and areal capacity of the implantation site[11,12] and limitations imposed by the maximum allowable specific absorption rate in small animals. For example, the size of an implantable receiving antenna mounted on the skull cannot exceed a diameter of 10-12 mm in mice[13]. Thus, it is challenging to support increasingly complex and power-hungry multifunctional devices with these approaches. Pioneering approaches that explore antennae implantation on the animal back have been demonstrated, however they require specialized surgical procedures and may experience misalignment induced by relative motion over extended experimental timescales[13,14].

Supplemental Note 4: Transient voltage pulse shaping removes capacitive artifacts

We hypothesized two possible sources of optical stimulation induced artifacts in our devices: 1) photoelectrochemical (PEC); 2) electromagnetic (EM). We quantified the PEC artifact at the electrode-electrolyte interface in a control experiment involving pulsed illumination of the tungsten electrodes with a 473 nm laser source in phosphate buffered saline (PBS) while simultaneously recording the electrode polarization versus a ground wire. No PEC artifacts were observed at a range of illumination intensities between 5-30 mW/mm^2. This is consistent with the large bandgap of native tungsten oxide layer (WO_3, 2.85 eV) which can function as a photoanode in presence of higher energy photons ($\lambda < 435$ nm)[15]. EM or capacitive artifacts are routinely observed in a device configuration that places high-voltage and fast-charging interconnects in close proximity with signal carrying electrode lines connected to a high impedance load. In our fibers the recording tungsten microelectrodes and the Ag-Cu interconnects separated by a polymer dielectric form a pair of capacitors (Fig. S14a). The

charging of these capacitors upon passage of μLED voltage signal produced robust artifacts that were time-locked to the start and end of the pulse with inverted polarities corresponding to charging and discharging currents. To minimize these artifacts, we utilized transient pulse shaping strategy that modified the rise/fall (r/f) times of voltage pulses. We observed a dramatic reduction of EM artifacts as the r/f times varied from 0.01 ms to 20 ms (Fig. S14 b-c). Notably this was observed for a range of optical intensities between 3-30 mW/mm^2 and pulse widths between 5-25 ms common in optogenetics studies. For pulses with r/f times >10 ms the generated artifact (10-20 μVpp) was found to be within the background noise level (~40 μVpp) and hence undetectable in the raw traces (Fig. S14 d-i). These findings were further corroborated with *in-vivo* opto-electrophysiology in the ventral tegmental area (VTA) of wild type (C57BL/6J) mice, wherein artifact-free traces were obtained for the trapezoidal shaped stimulation pulses (r/f time 10 ms) (Fig. S15 a-h). Thus, transient pulse shaping enabled artifact-free opto-electrophysiology with the microelectronics fibers.

Supplemental Note 5: Multisite, multifunctional fiber device enables simultaneous gut-brain implantation

We reasoned that developing and validating survival surgeries that allow multisite implantation of multifunctional fibers will motivate future studies of interoceptive neural circuits with these or similar multifunctional wireless devices. Given the miniature dimensions of research rodents, a multisite device should be controlled by a single wireless module connected to the common input/output (I/O) interface. However, this requirement prohibits mounting of the stiff brain fiber on a stereotaxic arm and restricts its motion in three dimensions. This, in turn, prevents accurate positioning of the fiber within a brain region of interest. To overcome these challenges, we developed a stretchable interconnect in the form of a helically coiled fiber with embedded metal microwires using thermal drawing. To produce such a fiber with broken axial symmetry we leveraged *in-situ* strain engineering achieved through the appropriate selection of the core and cladding polymers[16]. Strain engineering in thermally drawn fibers relies on accumulation of residual strain (pre-stretch) in an elastomeric core when it is drawn within a non-elastomeric cladding material, originating from differences in viscosities at the draw temperature. This stored strain energy can produce out-of-plane buckling of the elastomeric core if it is composed of materials with differing Young's moduli and has a non-centrosymmetric cross-sectional

geometry. Using these insights, we produced a conductive fiber-based stretchable interconnect composed of SEBS core with converged tungsten microwires inside a mechanically peelable poly(methyl methacrylate) (PMMA) cladding (Fig. S32 a-d). The dual-site gut-brain device that incorporates such a stretchable interconnect is shown in Fig. S32e. Stress-strain measurements on these interconnects showed that the constituent materials remain in the elastic response regime for uniaxial stretching exceeding 500%, which is over two orders of magnitude higher than the non-helical analogues (Fig. S32f). Current-voltage measurements of a μLED bonded to the interconnect at different levels of stretch (Fig. S32g) defined the functional threshold at which the interconnect transitions from an elastic to a plastic deformation, causing an open circuit (>200% strain). Electrical performance under continuous uniaxial cyclic stretching tests (10,000 cycles, 1 Hz, 50 and 100%) further highlighted their robust electromechanics, wherein a microscale surface-mount resistor bonded to the fiber was used as a signal read-out (Fig. S32h). As we demonstrate below, the typical mechanical deformation experienced by the interconnect during the surgical procedure remains well below their functional threshold value characterized here, thereby allowing us to freely extend the implantable fibers further out in space for precise stereotaxic implantation. Next, we developed a survival surgery with these multisite, multifunctional fiber devices by targeting implantation of the gut fiber in the proximal small intestine with concomitant implantation of the brain fiber in the VTA (Fig. S33a). The duodenal surgery was performed as previously described (see Methods section for details). Following the duodenal surgery, the brain fiber was implanted targeting the VTA. The free extension of the brain probe in three-dimensional space enabled by the stretchable helical fiber interconnect facilitated precise brain implantation (Fig. S33 b-d). Following one-week post-operative recovery, food intake, water intake, and locomotor behaviors were evaluated and compared to pre-operative values using a within-subject design. While food intake and water intake somewhat re-aligned between the dark and light cycles post-surgery, the surgery had no impact on overall food or water intake (Fig. S33 e-f). Moreover, there were no changes in locomotor activity in the implanted subjects (Fig. S33 g-h). These representative demonstrations exemplify use of wireless microelectronics fiber technology in combined gut-brain or multi-organ studies in awake behaving mice.

Supplemental Note 6: State-of-the-art in multifunctional bioelectronic interfaces

As summarized in Table S1, existing approaches to produce multifunctional bio-integrated devices for brain or peripheral organs exclusively rely on cleanroom-based lithography techniques[17–19]. Such resource intensive approaches are unsuitable for rapid design customization or modifications to device layouts as they require design of new photomasks for each pattern and optimization of the accompanying processing steps[17]. The thin film nature of lithography also precludes single-step, monolithic integration of multiple functionalities in a bioelectronic device. Thus, all previously reported schemes require independent fabrication of individual device modalities (multi-step process by itself) followed by careful manual assembly of layers to yield the final device[18,20,21]. Moreover, limitations on device scalability and lengths due to the use of silicon wafers as supporting substrates pose additional challenges for deployment in anatomically hard-to-reach sites in peripheral organs. The field of bioelectronics lacked alternative routes that overcame these limitations to produce multifunctional implants at scale for brain and peripheral organs without compromising biocompatibility and functional sophistication. Through this work we introduce an entirely new class of multifunctional and wirelessly capable polymer-based microelectronic fibers that enable organ-specific interrogation of neuronal and non-neuronal cell-types that govern animal behavior. To accomplish this, we combine the scalability and design customization afforded by multimaterial thermal drawing[4] with the sophistication of semiconductor microelectronics. This approach allows us to deterministically tune the device mechanics to produce microscale wireless interfaces for the anatomically and functionally disparate organs such as the brain and the GI tract. In contrast to thin-film lithographic approaches, our method uniquely allows one-step, monolithic device fabrication in a manner that produces sub-km lengths of microstructured polymer filaments starting from a macroscale preform. This preform-to-device route also lends itself favorably for integrating solid-state microelectronic chips as demonstrated in Fig. S3 wherein we engineer electrical bonding of metal interconnects as well as *in-situ* packaging during the drawing process. This enables us to produce fully packaged and electrically pre-bonded multifunctional microelectronic fibers at scale.

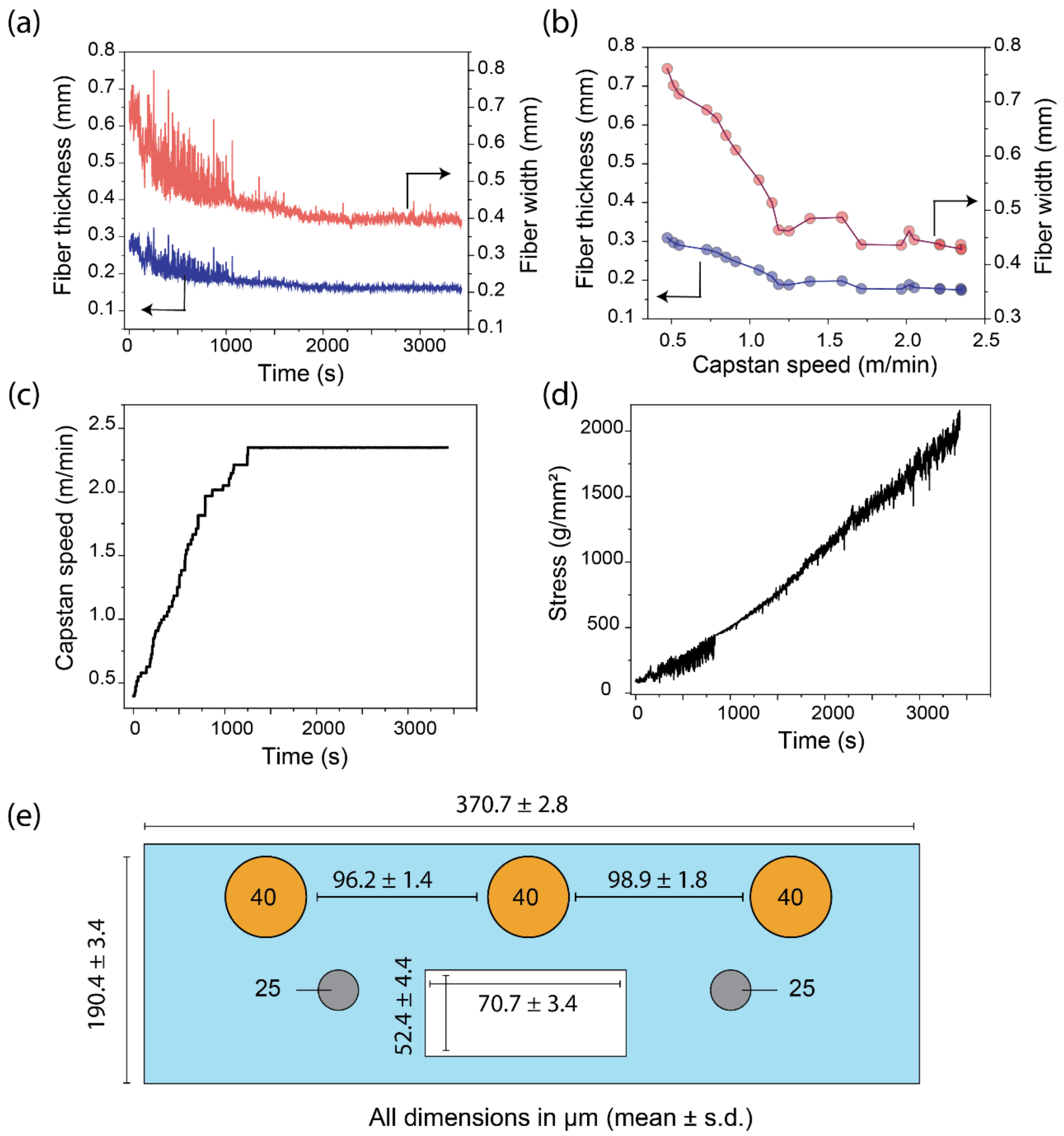

Figure S1. Thermal drawing data for the brain fibers

(a) Variation of fiber width (red trace) and thickness (blue trace) during the draw, where arrows indicate the corresponding y-axes for respective plots; **(b)** Variation of fiber width (red trace) and thickness (blue trace) with capstan speed at fixed preform feed-rate of 1 mm/min, where arrows indicate the corresponding y-axes for respective plots; **(c)** Variation of capstan speed during the draw; **(d)** Variation of fiber stress during the draw; **(e)** Average dimensions of the fiber features over 5 randomly selected cross-sections.

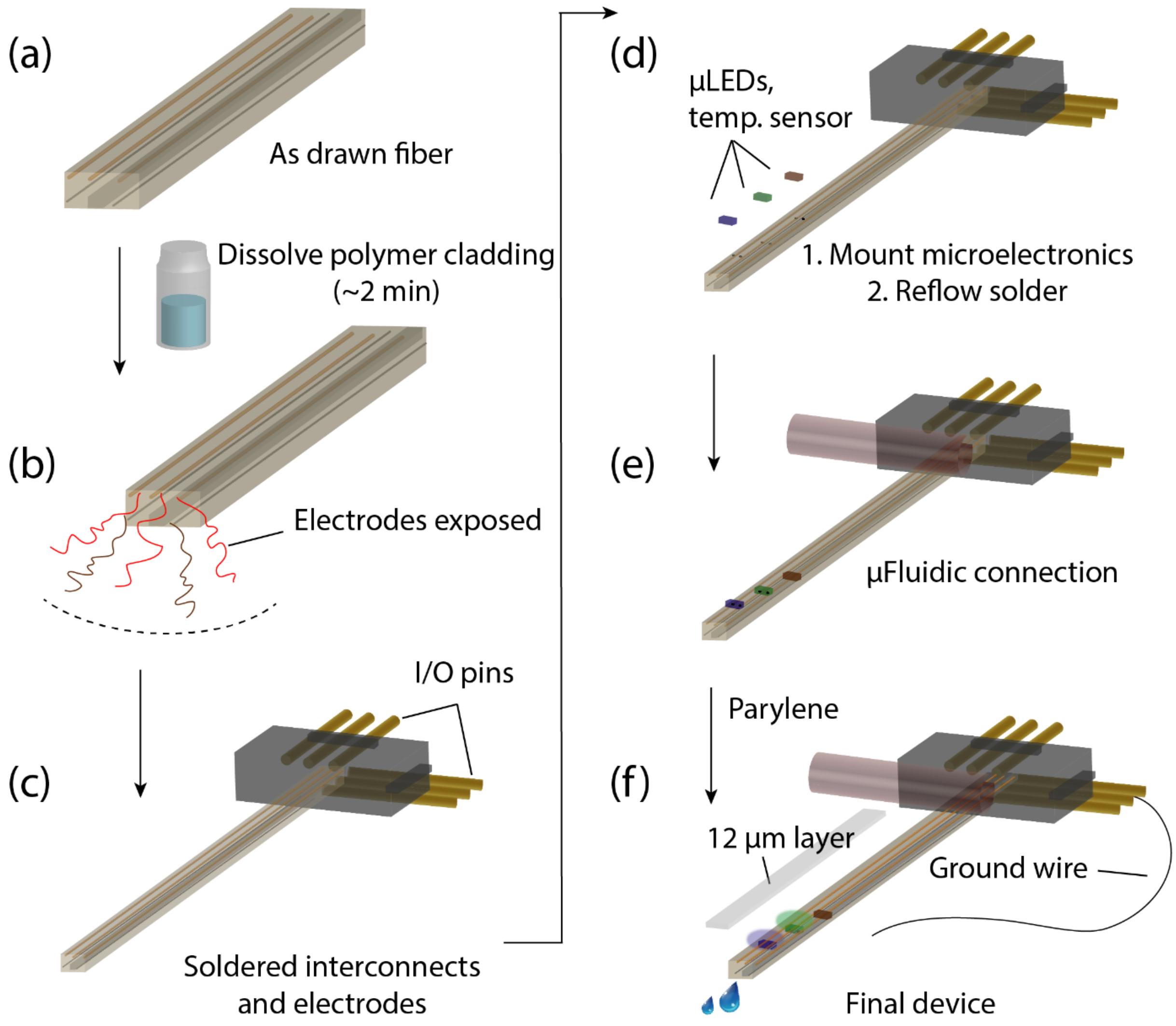

Figure S2. Process flow for fiber connectorization

(a) The PC or SEBS cladding is etched away in an organic solvent such as dichloromethane; **(b)** The embedded metal microwires are exposed and isolated; **(c)** The microwires are soldered to header pins; **(d)** The µLEDs and thermal sensors are mounted on the fiber surface with reflow soldering or thermally curable conductive silver epoxy; **(e)** The microfluidic channel is connected to external access tubing with a T-junction; **(f)** Finally, the ground wire is soldered onto a header pin and parylene-C is vapor deposited onto the fiber.

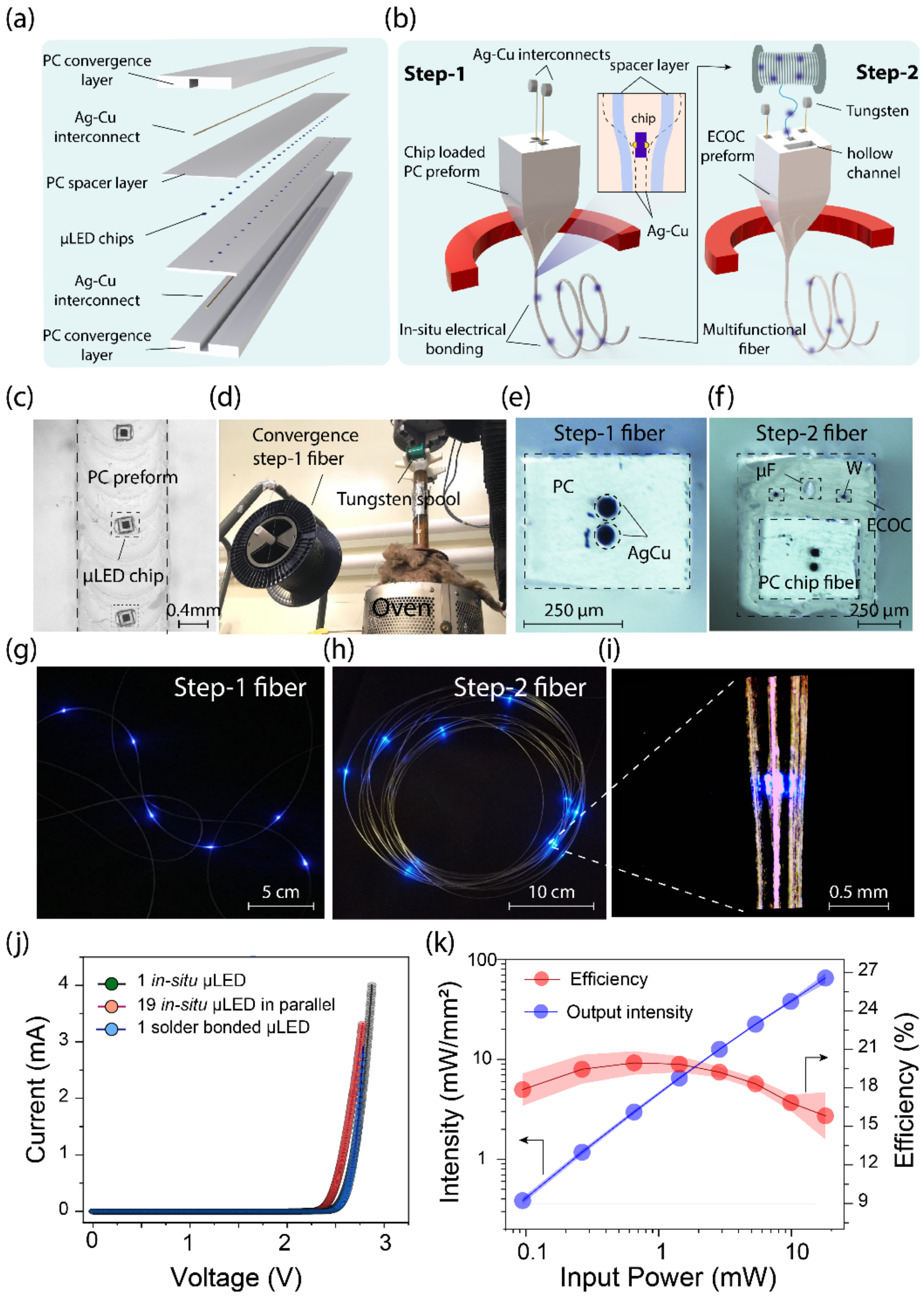

Figure S3. Integration of microelectronic devices at the preform stage permits *in-situ* electrical bonding and packaging in a multifunctional fiber

(a) Multilayered sandwiched configuration of the preform consisting of two convergence layers, two spacer layers, and a central chip layer; **(b)** (left) Step-1 involves drawing of the chip loaded

preform with simultaneous feeding of Ag-Cu interconnects, inset shows the *in-situ* electrical bonding process during the draw, (right) Step-2 involves feeding the fiber obtained from Step-1 into another ECOC preform that can host tungsten microelectrodes and a microfluidic channel; **(c)** Optical micrograph of a μLED loaded central chip layer; **(d)** A photograph showing feeding of Step-1 fiber along with tungsten microwire spools in the ECOC preform; **(e)** Cross-sectional micrograph of Step-1 fiber; **(f)** Cross sectional micrograph of Step-2 fiber; representative photographs showing successful operation of several *in-situ* electrically bonded μLEDs from **(g)** Step-1 fiber and; **(h)** Step-2 fiber; **(i)** Optical micrograph of a fiber-integrated μLED fully packaged in a double layer insulating cladding of PC and ECOC; **(j)** I-V characteristics of *in-situ* fiber integrated blue μLEDs (green and red traces) after Step-2 showing robust electrical connection that is comparable to a reflow-soldered μLED (blue trace); **(k)** Light output and efficiency of *in-situ* integrated μLED as a function of input electrical power (n=4 random sections) where arrows indicate the corresponding y-axes for respective plots. Data are presented as mean values +/- s.d. All shaded areas and error bars represent s.d.

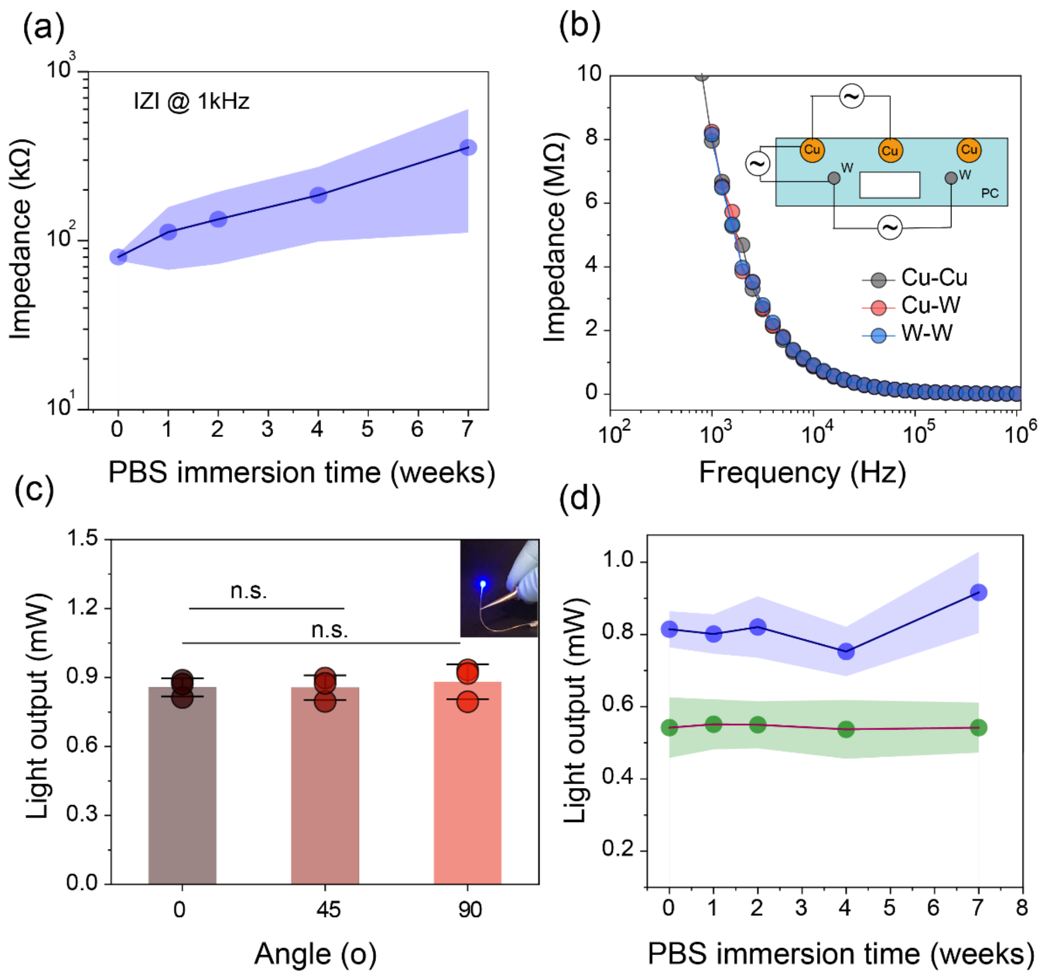

Figure S4. Electrical and optical characterization of the brain fibers

(a) Electrochemical impedance of tungsten microelectrodes at 1 kHz upon immersion in phosphate buffered saline (PBS) bath at 37 °C over 7-weeks (n=6 devices); **(b)** Measurement of interwire impedance through the polymer cladding in a PBS electrolyte; **(c)** Light output from the fiber device bent at different angles; 45°: p = 0.97396, *F-value*: 0.00121; 90°: p = 0.64752, *F-value* = 0.24356 (n=3 fibers each, One-way ANOVA and Tukey's comparison test); **(d)** Light output from the fiber device that was immersed in PBS bath at 37 °C over 7-weeks (blue trace, 470 nm µLED; green trace, 527 nm µLED, n=4 devices each). Data are presented as mean values +/- s.d. All shaded areas and error bars represent s.d.

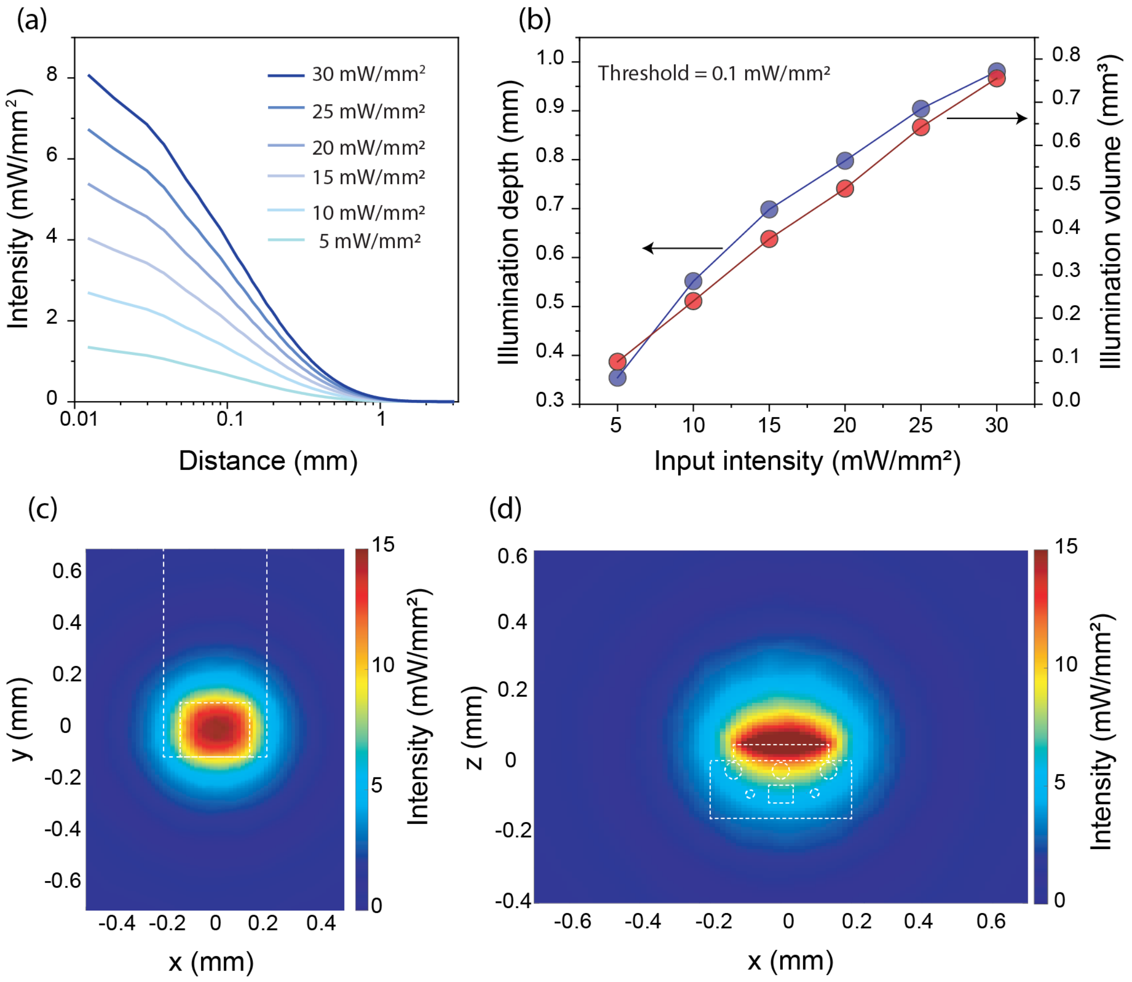

Figure S5. Optical FEM simulations of brain fibers in the brain tissue

(a) Variation of optical intensity with distance from surface of a fiber µLED ($\lambda = 470$ nm) at input intensities between 5-30 mW/mm^2; **(b)** Variation of illumination depth and volume with different input optical intensities ($\lambda = 470$ nm) where arrows indicate the corresponding y-axes for respective plots; **(c-d)** Spatial distribution of optical intensity at 30 mW/mm^2 around the fiber tip in the **(c)** x-y and; **(d)** x-z plane.

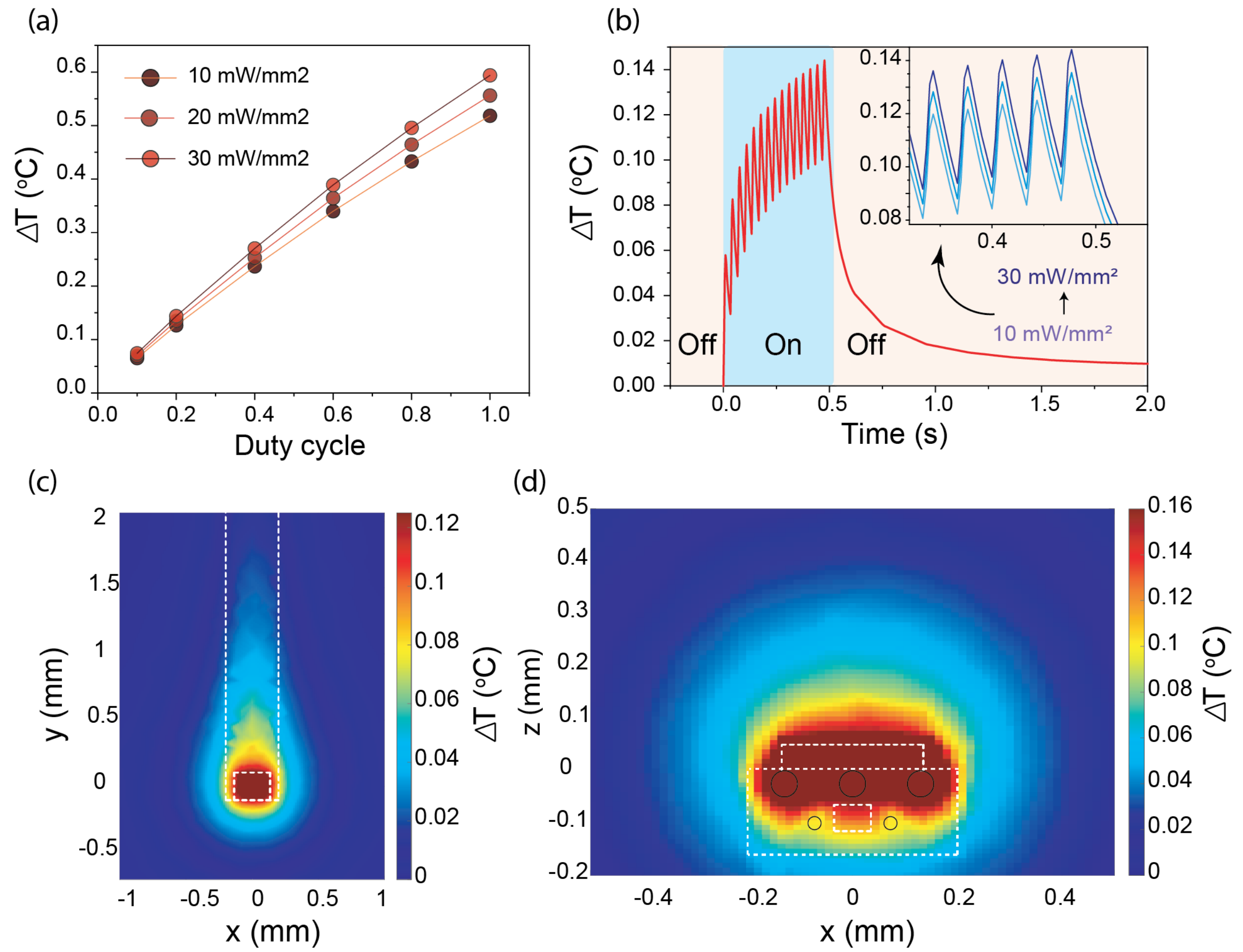

Figure S6. Thermal FEM simulation of brain fibers in the brain tissue

(a) Variation of local temperature at the surface of the fiber μLED (λ = 470 nm) as a function of duty cycle and optical intensity; **(b)** Temporal variation of temperature at the surface of fiber μLED at the end of a physiologically relevant optical stimulation pulse train (20 Hz, 20% duty cycle) and intensities of 10, 20, and 30 mW/mm^2; **(c, d)** Spatial distribution of temperature around the fiber tip at the end of an optical stimulation pulse train (30 mW/mm^2, 20 Hz, 20% duty cycle) in the **(c)** x-y and; **(d)** x-z plane.

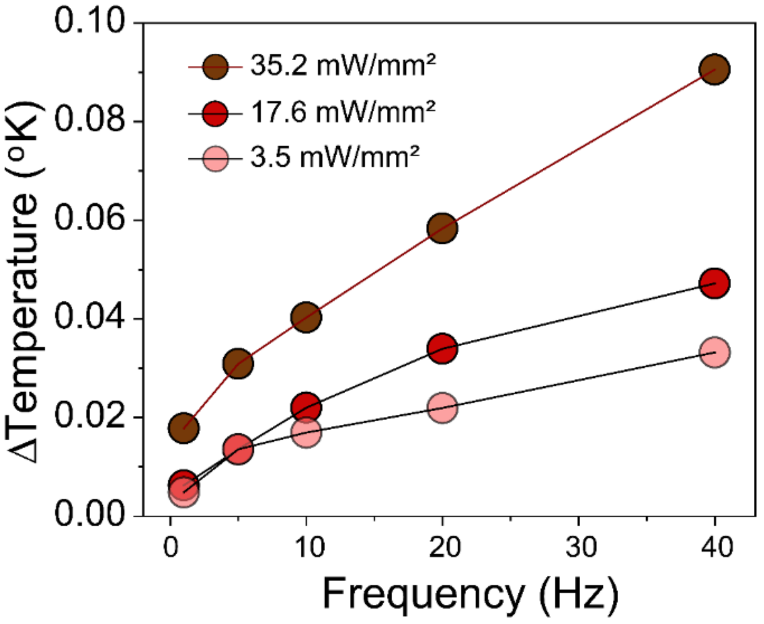

Figure S7. Characterization of thermal sensing modality of the brain fiber

Changes in local temperature around a fiber μLED (λ = 470 nm) detected by a co-located diode based thermal sensor while the μLED is operated at different optical stimulation frequencies and intensities.

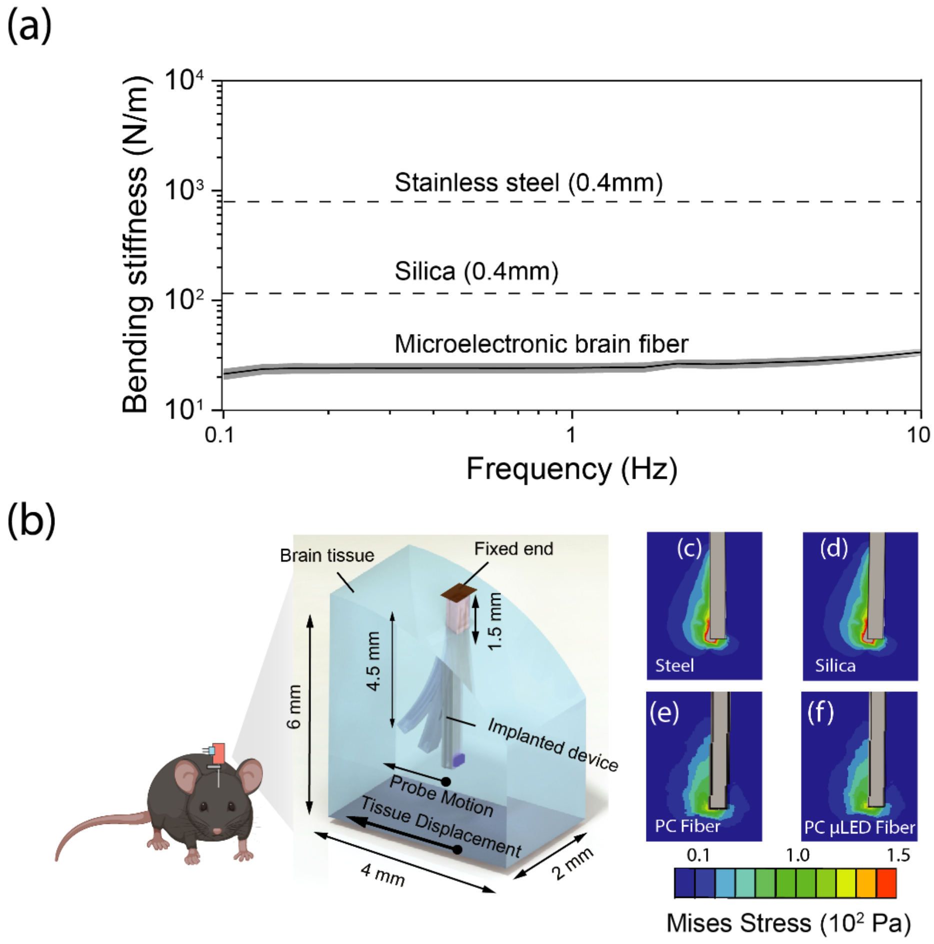

Figure S8. Mechanical FEM simulation of the brain fiber in the brain tissue

(a) Experimental bending stiffness measurement of PC brain fiber (n=3, length = ~1 cm) in a single cantilever mode with frequency sweep and the corresponding theoretical values for a silica waveguide (400 µm, length = 1 cm) and stainless steel wire (400 µm, length = 1cm); **(b)** 3D FEM model that captures the micromotion between fiber implant in brain tissue in the VTA, mimicking a single cantilever mode; **(c-f)** Spatial distribution of the misses stress around the implant tip at brain tissue displacement of 100 µm for **(c)** 400 µm steel wire; **(d)** 400 µm silica waveguide; **(e)** bare PC fiber and; **(f)** PC fiber with µLEDs. Data are presented as mean values +/- s.d. All shaded areas represent s.d.

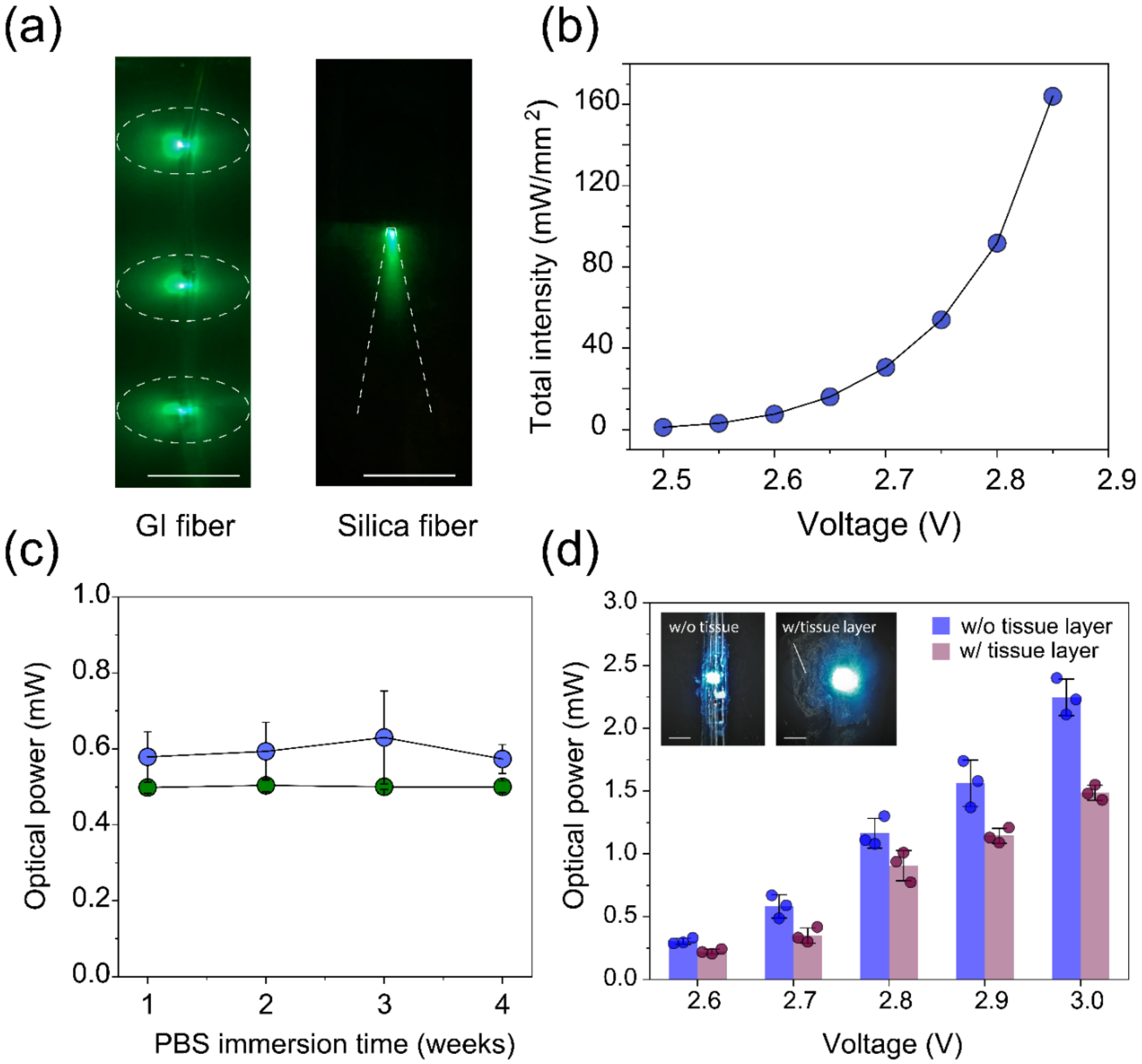

Figure S9. Optical characterization of soft gut fiber

(a) Lateral illumination profile from gut fiber (three 470 nm μLEDs, 10.6 mW/mm^2) as compared to dorsal-ventral oriented light cone from silica waveguide (right) in a 0.1 mM fluorescein solution (right), scale bar 1 cm; **(b)** Cumulative light output from three λ = 470 nm fiber μLEDs connected in parallel; **(c)** Light output from the gut fiber over the course of 4 weeks while being immersed in a PBS bath at 37 ^{o}C for blue (n=3 independent samples) and green (n=3 independent samples) μLEDs; **(d)** Light output from the gut fiber with (n=3 independent samples) and without (n=3 independent samples) an overlaid layer of intestinal tissue. (Inset scale bar 0.5 mm). Data are presented as mean values +/- s.d. All error bars represent s.d.

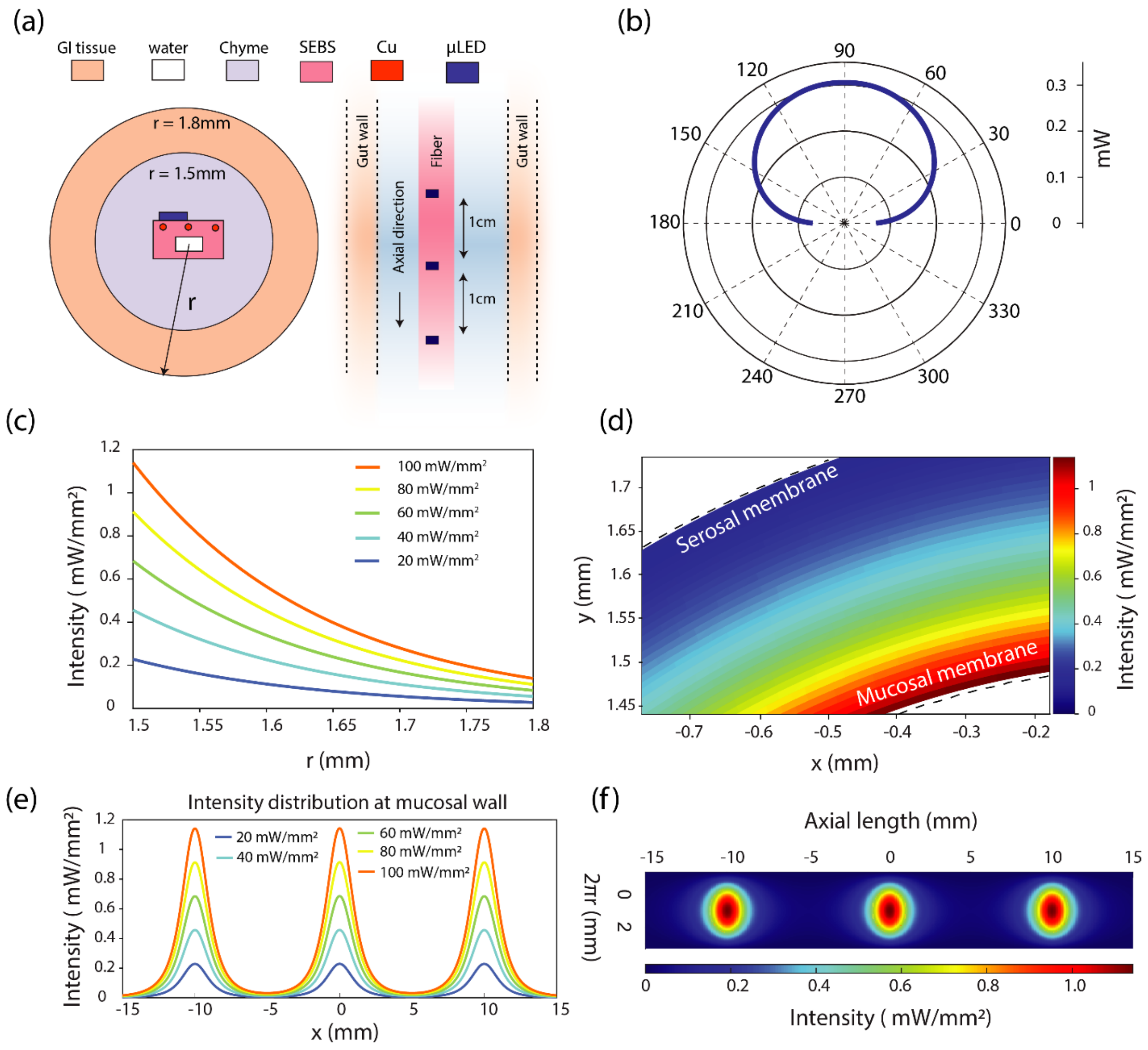

Figure S10. Optical FEM simulations of gut fiber in the intestinal lumen

(a) Schematic of the 3D optical FEM model showing the architecture of the gut fiber and dimensions of the small intestine (left: cross sectional view; right longitudinal view); **(b)** Fitted angular distribution of the µLED power emission profile (λ = 470 nm); **(c)** Radial light intensity distribution between the mucosal (r = 1.5 mm) and serosal membrane (r = 1.8 mm) as a function of µLED emission power; **(d)** Cross-sectional light intensity distribution between the mucosal and serosal membrane at µLED emission power of 100 mW/mm^2; **(e)** Axial light intensity profile at the mucosal wall as a function of µLED emission power; **(f)** Flattened light intensity

distribution profile projected onto the top half of the mucosal membrane plane at a µLED emission power of 100 mW/mm^2.

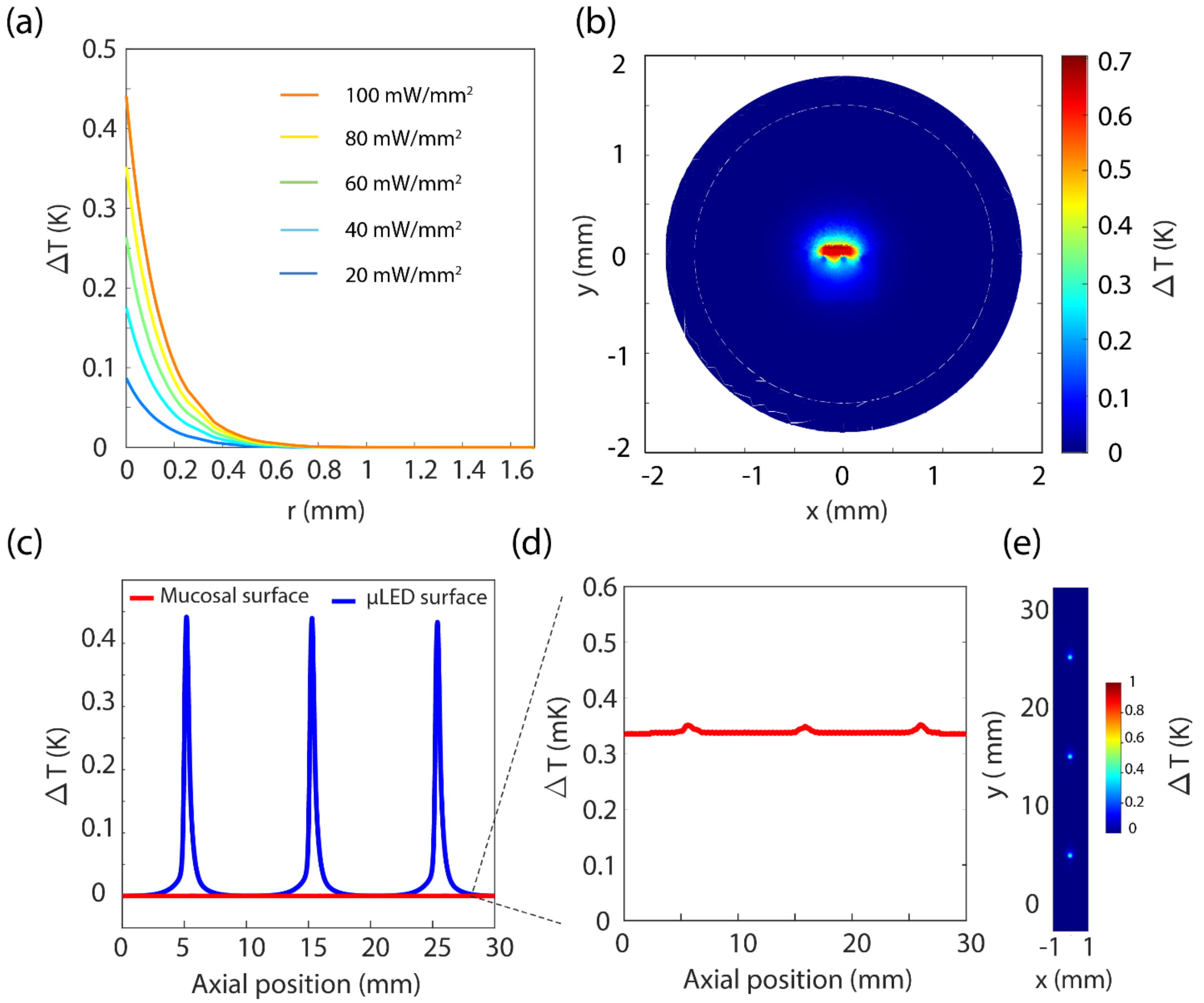

Figure S11. Thermal FEM simulation of the gut fiber in the small intestine lumen

(a) Radial profile of the change in temperature as a function of µLED emission power within the intestinal lumen; **(b)** Flattened profile of temperature change on the mucosal membrane at a µLED emission power of 100 mW/mm^2; **(c)** Axial profile of temperature change across the top µLED surface (blue) and on the mucosal surface (red) at an emission power of 100 mW/mm^2; **(d)** Enlarged axial profile from (c) of temperature change on the mucosal surface; **(e)** Planar cross-sectional profile of temperature change across the top µLED surface at an emission power of 100 mW/mm^2.

(a)

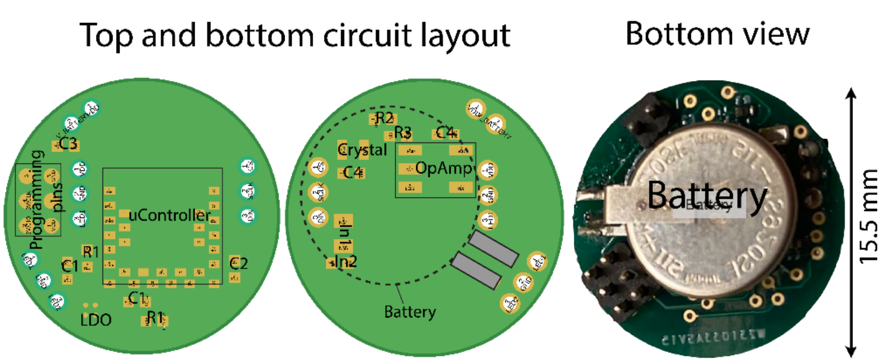

(b)

Code	Component
R1	470Ω
R2	1kΩ
R3	1MΩ
C1	0.47μF
C2	1μF
C3	10μF
C4	12pF
In1	10 μH
In2	15 nH
OpAmp	AD8603
Crystal	32 kHz, ABS05
LDO	LP5900TL
μController	MDBT42V-P512KV2

(c)

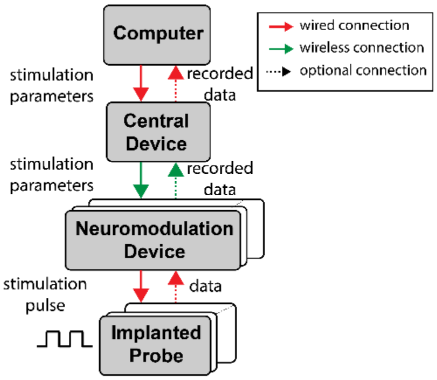

(d)

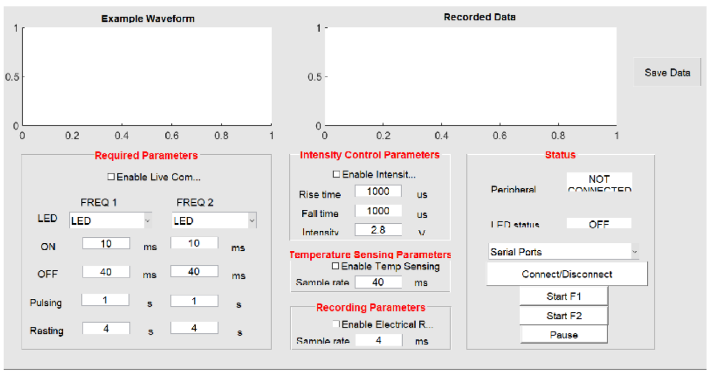

(e)

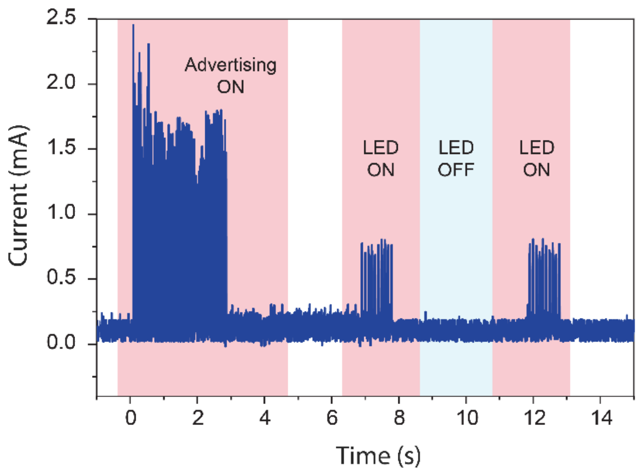

(f)

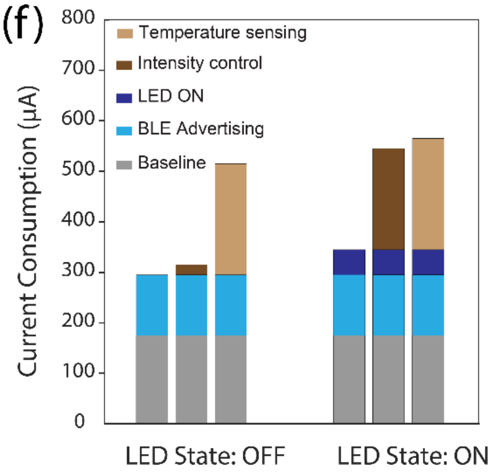

Figure S12. Circuit layout of NeuroStack primary module, graphical user interface (GUI), and power consumption profile across different modalities

(a) Top and bottom circuit layout along with a photograph showing the bottom view of the NeuroStack module which hosts a rechargeable battery; **(b)** Component details for NeuroStack module; **(c)** Flow of information between the neural probe and the base-station computer. The central board can control up to four neuromodulation devices mounted on different animals; **(d)** The GUI implemented in MATLAB to control and communicate with the neuromodulation device; **(e)** Dynamic and **(f)** steady-state power breakdown of the NeuroStack module where the total power consumption scales with other enabling functions such as temperature sensing and intensity control.

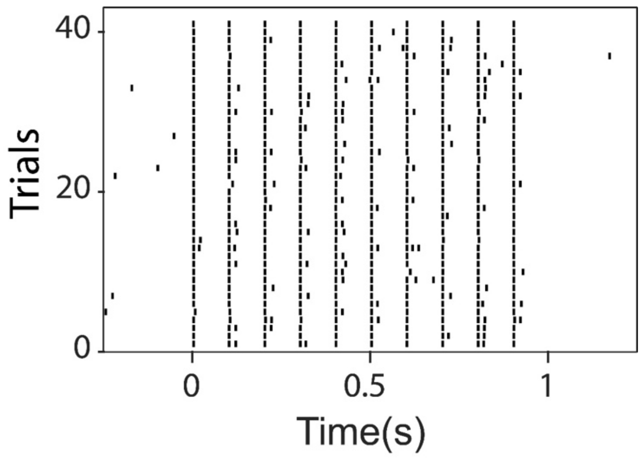

Figure S13. Extended data for figure 5d

Raster plot over 40 stimulation trials confirms reproducibility of the optically evoked electrical activity in mice expressing ChR2 in dopaminergic neurons on Day-20 following the combined viral injection and device implantation surgery.

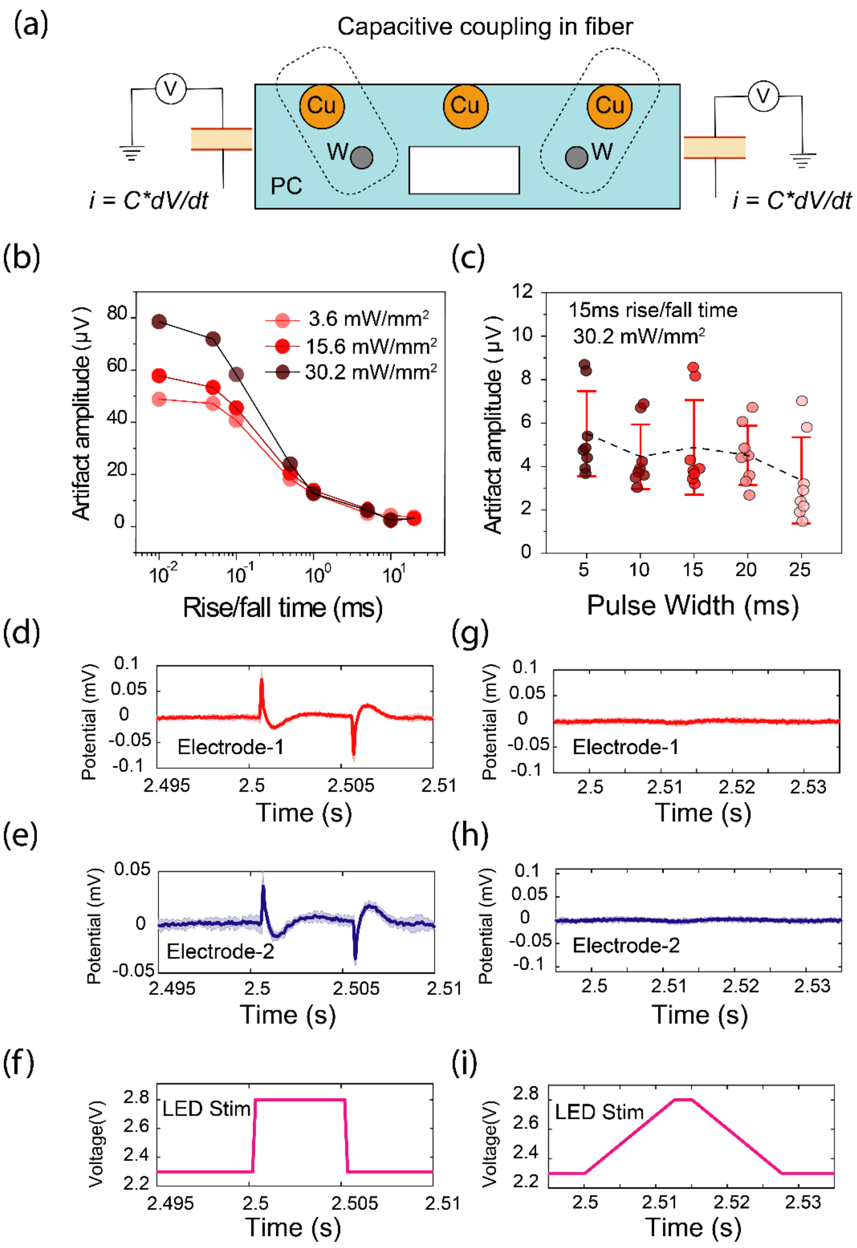

Figure S14. *In-vitro* quantification of capacitively coupled artifacts in brain fiber

(a) Schematic illustration of capacitive coupling between interconnect and electrode microwires separated by a polymer dielectric; **(b)** Artifactual signals recorded at the tungsten microelectrode in PBS vary with the rise /fall time of μLED optical stimulation pulses (5 ms pulse width, 10 Hz) at different intensities; **(c)** Artifact amplitude is invariant to changes in optical stimulation pulse width (n= 4 independent fiber samples, 2 electrodes per fiber); **(d-f)** signals recorded at both tungsten microelectrodes along with corresponding optical stimulation shape for square pulse

with 0.01 ms rise/fall time and; **(g-i)** trapezoidal pulse with 12.5 ms rise/fall time. Data are presented as mean values +/- s.d. All shaded areas and error bars represent s.d.

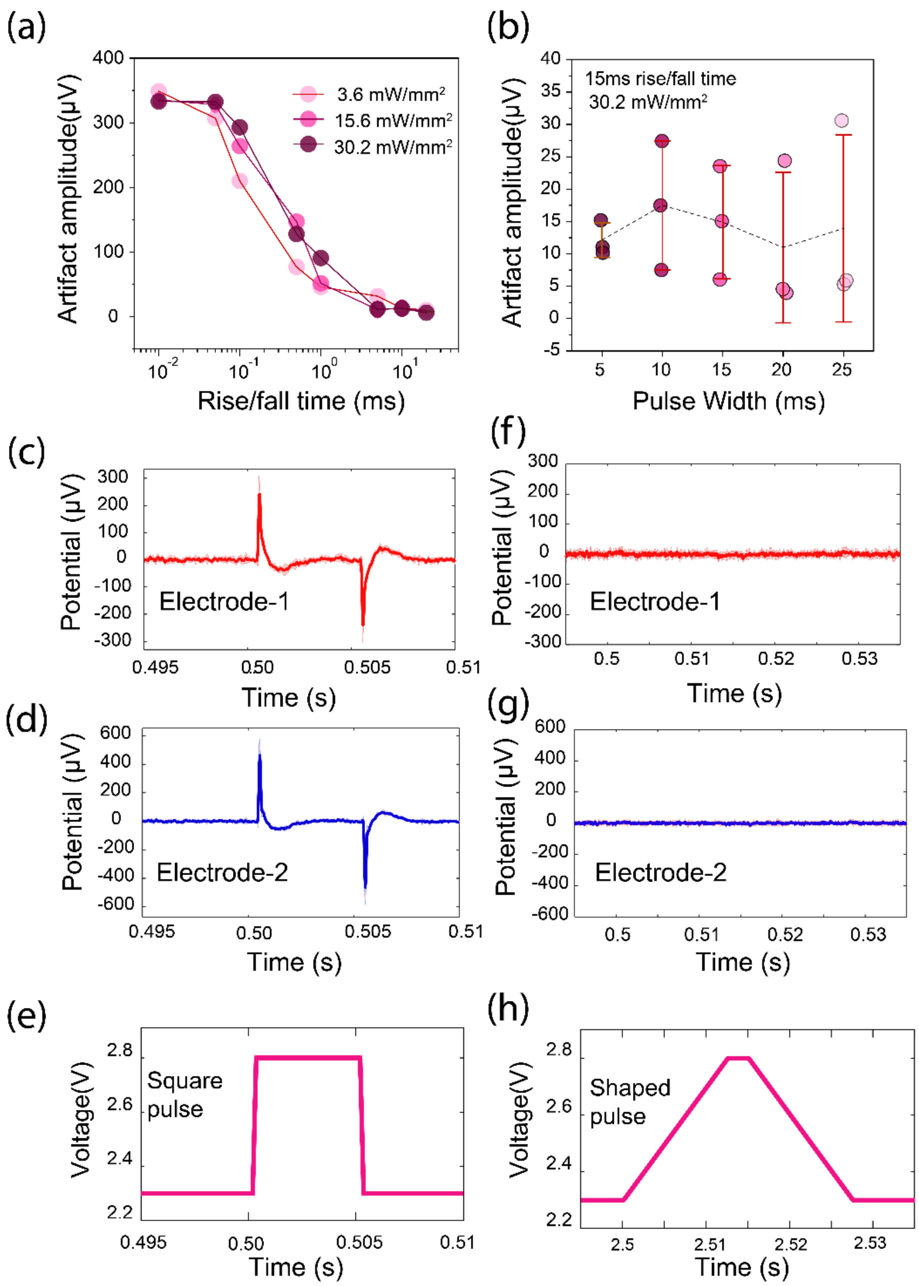

Figure S15. ***In-vivo*** **quantification of capacitively coupled artifacts in brain fiber**

(a) Artifact amplitude recorded at the tungsten microelectrode in ventral tegmental area (VTA) of C57BL/6J mice varies with the rise /fall time of µLED optical stimulation pulses at varying intensities; **(b)** Artifact amplitude is invariant to changes in optical stimulation pulse width (n=3 mice); **(c-e)** signals recorded at both tungsten microelectrodes along with corresponding optical stimulation shape for a square pulse with 0.01 ms rise/fall time and; **(f-h)** trapezoidal pulse with

12.5 ms rise/fall time. Data are presented as mean values +/- s.d. All shaded areas and error bars represent s.d.

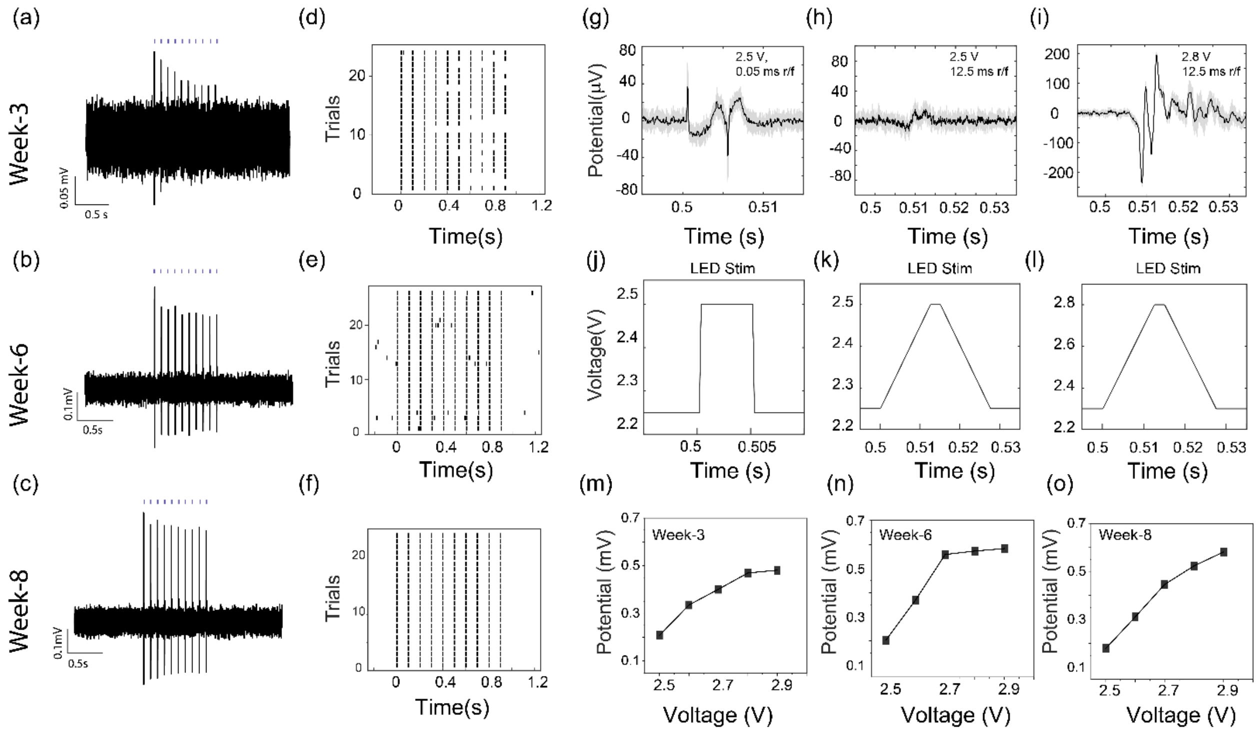

Figure S16. Chronic recordings of optically evoked electrical activity in mice transduced with ChR2 in dopaminergic (DA) neurons

Simultaneous optogenetic stimulation (470 nm, 10.3 mW/mm^2, 5 ms pulse width, 10 Hz, 1 s ON, 4 s OFF, blue markers indicate pulse onset) and recording of neural activity in the VTA of chronically implanted mice expressing ChR2 in DA neurons at **(a)** 3-weeks; **(b)** 6 weeks; **(c)** 8-weeks after viral transfection and fiber implantation surgery; **(d-f)** corresponding raster plots over >20 stimulation trials; **(g,j)** Optical stimulation with square pulse produces capacitively coupled artifacts; **(h,k)** Transient pulse shaping suppresses the capacitive artifact; **(i,l)** Multiunit activity recorded in response to shaped optical pulse (15.6 mW/mm^2, 12.5 ms r/f, 10 Hz); Amplitude of multiunit activity in response to optical stimulation (5 ms pulse width, 10 Hz, 1 s ON, 4 s OFF) increases with increasing stimulation intensity at **(m)** week-3; **(n)** week-6; and **(o)** week-8 time point post implantation for a representative animal. All shaded areas and error bars represent s.d.

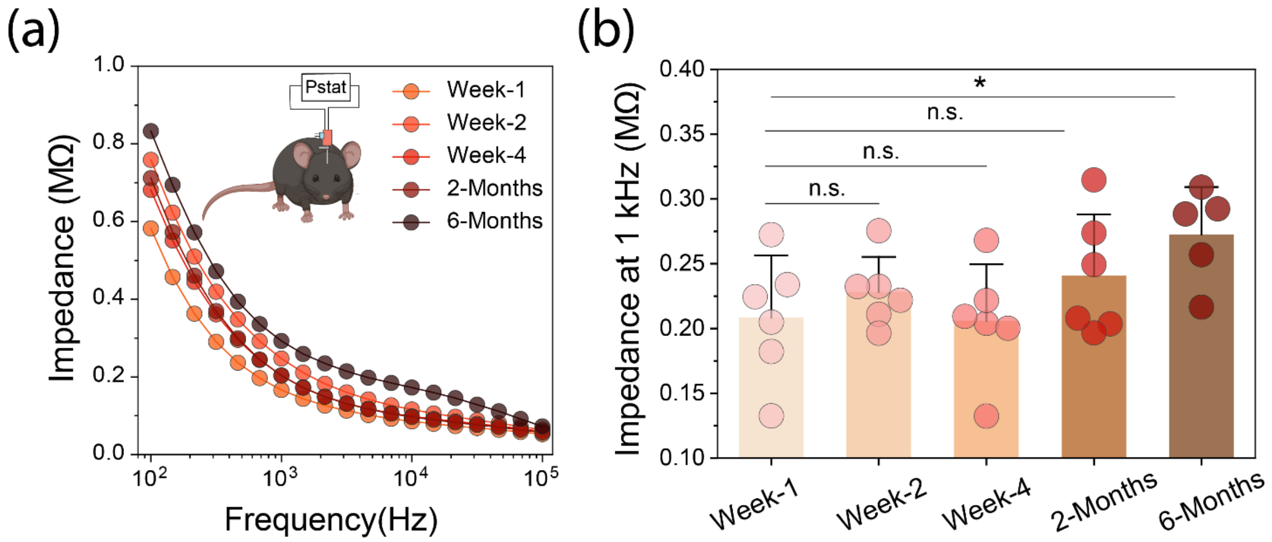

Figure S17. *In-vivo* stability of recording electrodes over 6 months

(a) Impedance spectra of a tungsten microelectrode in a chronically implanted brain fiber from week-1 to 6-months post implantation; **(b)** Corresponding values of the impedance modulus at 1 kHz. Week-2: p = 0.8292, 1-month: p = 0.98448, 2-month: p = 0.64561, 6-month: p = 0.02197 (n=3 mice, 2 electrodes per fiber, repeated measures one-way ANOVA followed by Tukey's post-hoc comparison test). Data are presented as mean values +/- s.d. All error bars represent s.d.

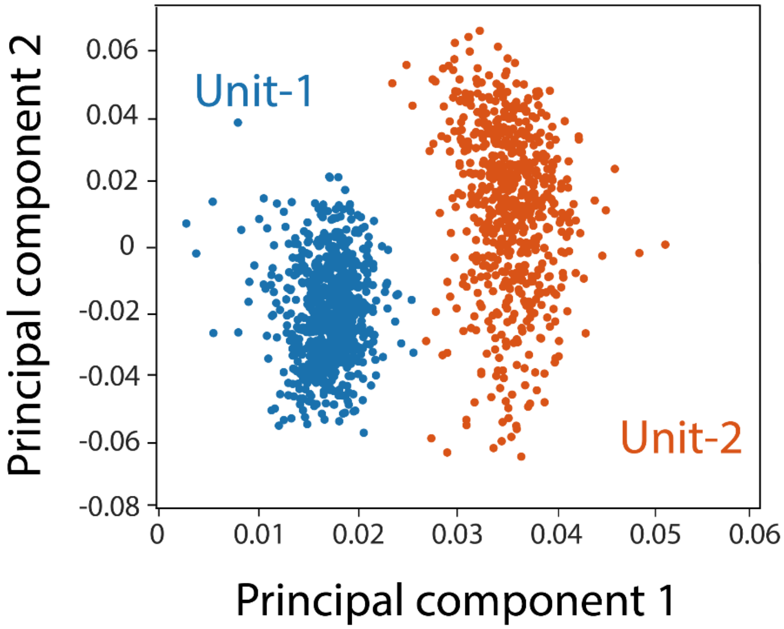

Figure S18. Extended data for figure 5e

Clusters revealed by principal component analysis of isolated action potentials at week-4 in the VTA.

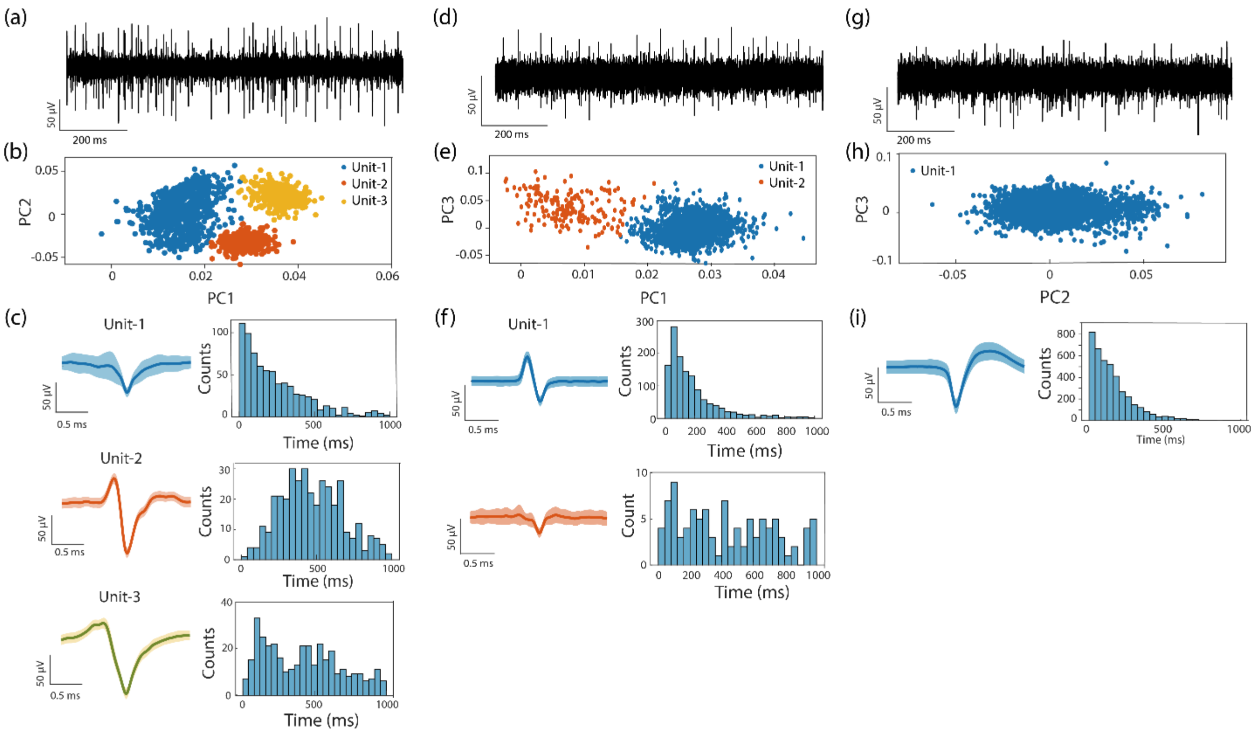

Figure S19. Chronic recording of single unit activity in the VTA at week-2 following implantation

(a,d,g) Raw traces of endogenous activity; **(b,e,h)** Clusters of single units revealed by principal component analysis; **(c,f,i)** Average spike waveforms (left column) corresponding to clustered units and their inter-spike-interval histograms (right column). Data from 3 separate animals. All shaded areas represent s.d., data are presented as mean values +/- s.d.

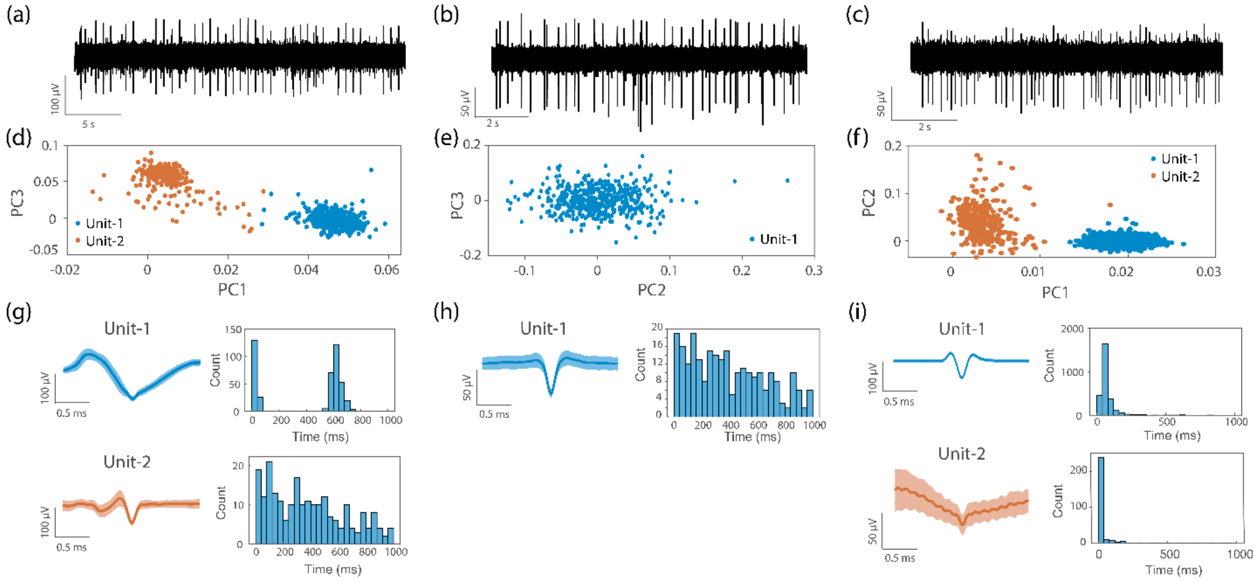

Figure S20. Chronic recording of single unit activity in the VTA at week-4 following implantation

(a-c) Raw trace of endogenous activity; **(d-f)** Clusters of single units revealed by principal component analysis; **(g-i)** Average spike waveforms corresponding to clustered units (left column) and their inter-spike-interval histograms (right column). Data from 3 separate animals. All shaded areas represent s.d., data are presented as mean values +/- s.d.

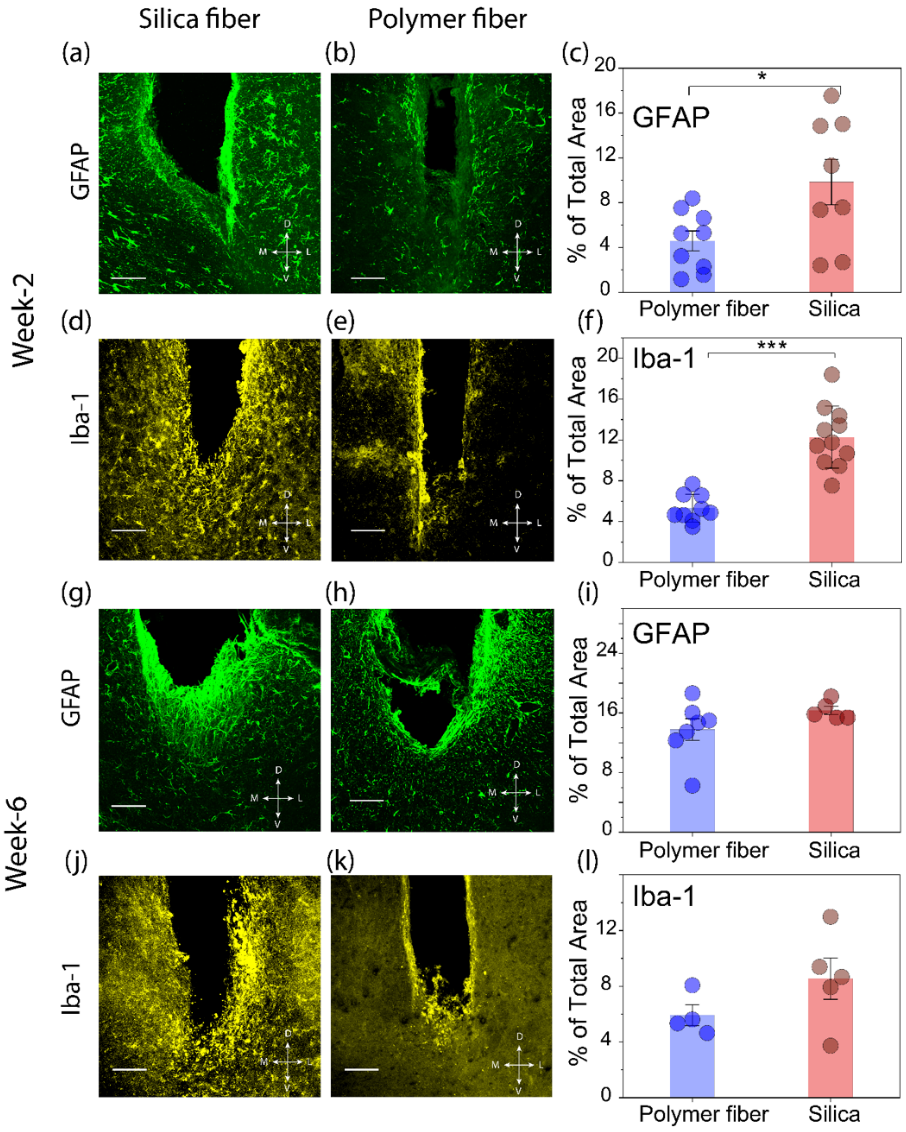

Figure S21. Immunohistochemical evaluation of biocompatibility of chronically implanted brain fiber

Astrocytic (GFAP) and microglia (Iba1) immunoreactivity surrounding a microelectronic polymer fiber and a 300 μm silica fiber, along with average fluorescence intensity quantifying the presence of Iba1 and GFAP at the implant tips at **(a-f)** week-2; and **(g-l)** week-6 time points post implantation (scale bar 100 μm). GFAP week-2; $p = 0.04044$, $t = -2.36714$ (n =9 polymer fiber traces and n=8 silica fiber traces, Welch's t-test); Iba1 week-2; $p = 5.68306\times10^{-6}$, t= -

6.33794 (n =9 polymer fiber traces and n=11 silica fiber traces, two-sample t-test). GFAP week-6; $p = 0.18663$, $t = -1.41785$ (n =7 polymer fiber traces and n=5 silica fiber traces, two sample *t*-test); Iba1 week-6; $p = 0.18948$, $t = -1.4532$ (n = 4 polymer fiber traces and n = 5 silica fiber traces, two sample *t*-test). Data are presented as mean values +/- s.d. All error bars represent s.d.

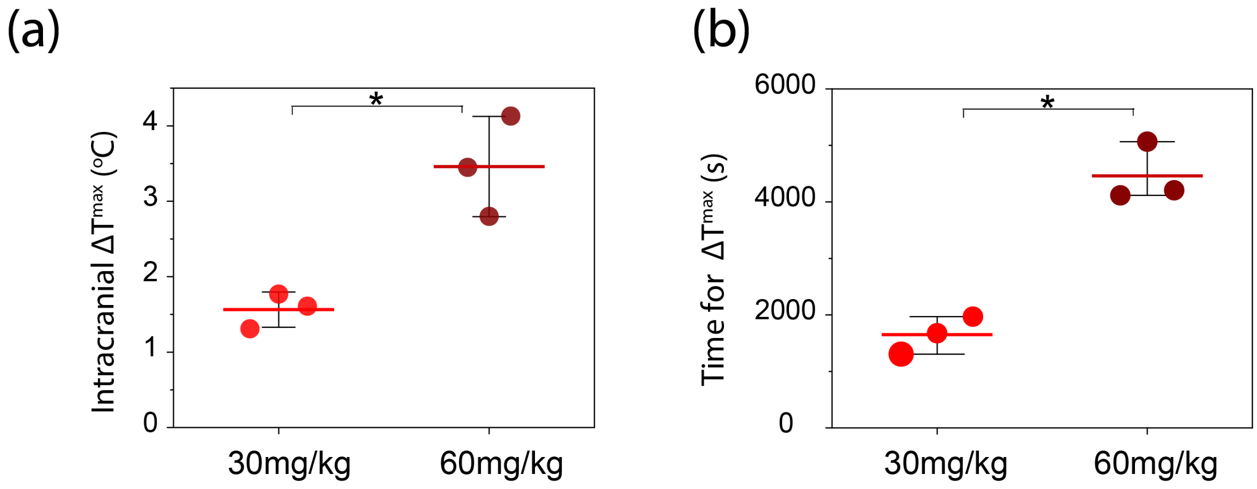

Figure S22. Temperature sensing modality in the fiber detects anesthesia-induced brain hypothermia in a dose dependent manner

(a) Anesthesia induced brain hypothermia recorded in the VTA by the thermal sensor within the fiber in response to an intraperitoneal injection of Ketamine/Xylazine mixture at 30 mg/kg (n=3 mice) and 60 mg/kg doses (n=3 mice) (p = 0.00958, t = -4.66065, two sample t-test); **(b)** corresponding time interval after which intracranial temperature begins to rise (n=3 mice for each condition, p = 0.00143, t = -7.83279, two sample t-test). Data are presented as mean values +/- s.d. All error bars represent s.d.

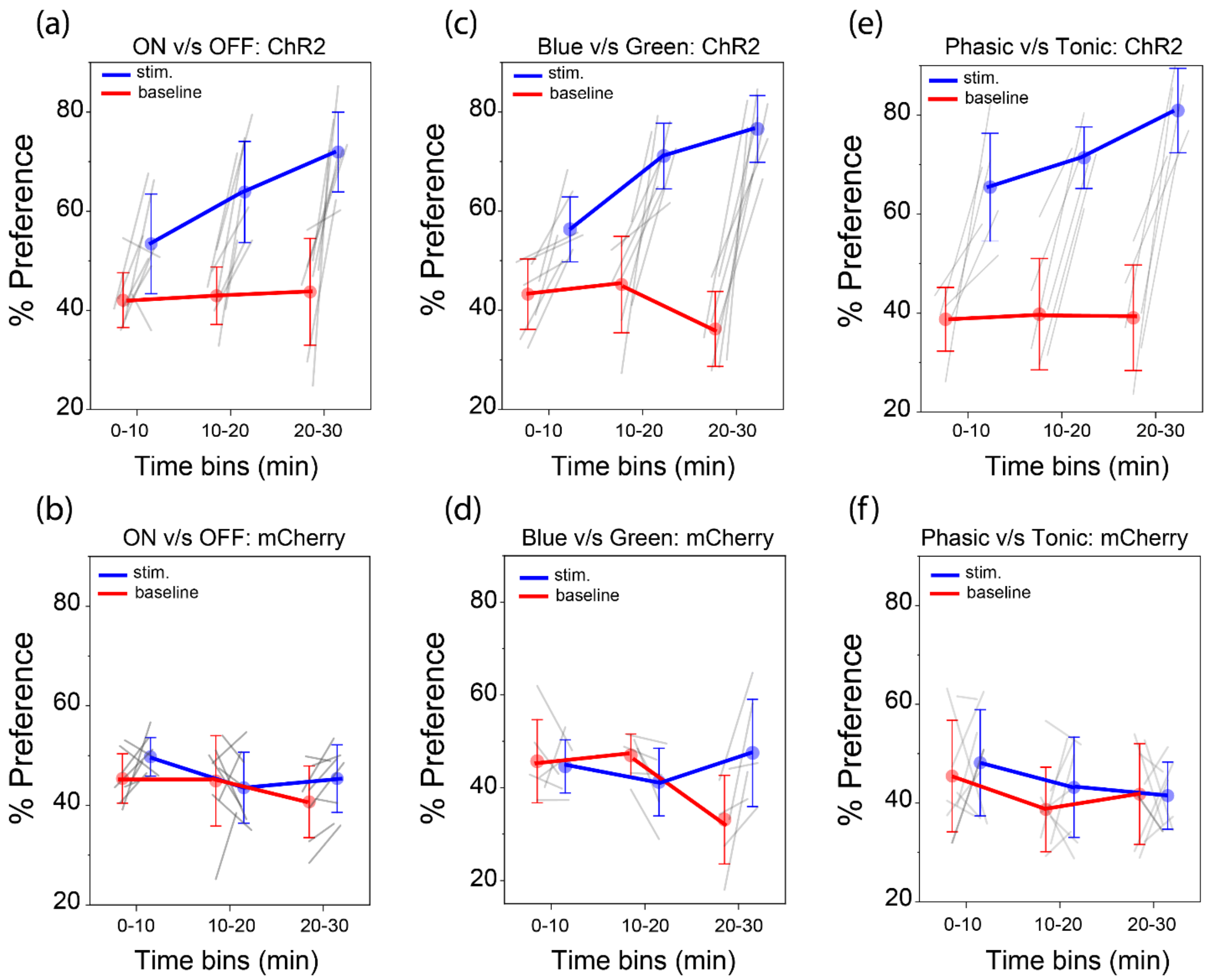

Figure S23. Wireless programmable optical stimulation of DA neurons in the VTA progressively imparts place preference

Preference (%) in a real-time place preference (RTPP) task binned at 10 min time intervals for **(a)** ChR2-mCherry mice in ON (λ = 470 nm, 25 Hz, 10 ms pulse, 1 s ON, 2 s OFF) v/s OFF condition (n=8 mice); **(b)** mCherry control mice in ON (λ = 470 nm, 25 Hz, 10 ms pulse, 1 s ON, 2 s OFF) v/s OFF condition (n=7 mice); **(c)** ChR2-mCherry mice in blue light (λ = 470 nm, 25 Hz, 10 ms pulse, 1 s ON, 2 s OFF) v/s green light stimulation (λ =527 nm, 25 Hz, 10 ms pulse, 1 s ON, 2 s OFF) condition (n=8 mice); **(d)** mCherry mice in blue light (λ = 470 nm, 25 Hz, 10 ms pulse, 1 s ON, 2 s OFF) v/s green light stimulation (λ =527 nm, 25 Hz, 10 ms pulse, 1 s ON, 2 s OFF) condition (n=6 mice); **(e)** ChR2-mCherry mice in Phasic bursting (λ = 470 nm, 40 Hz, 5ms pulse, 0.5 s ON, 4 s OFF) v/s tonic stimulation (λ = 470 nm, 5 Hz, 5 ms pulse) condition (n=6 mice); **(f)** mCherry mice in phasic bursting (λ = 470 nm, 40 Hz, 5ms pulse, 0.5 s ON, 4 s

OFF) v/s tonic stimulation (λ = 470 nm, 5 Hz, 5 ms pulse) condition (n=8 mice). Data are presented as mean values +/- s.d. All error bars represent s.d.

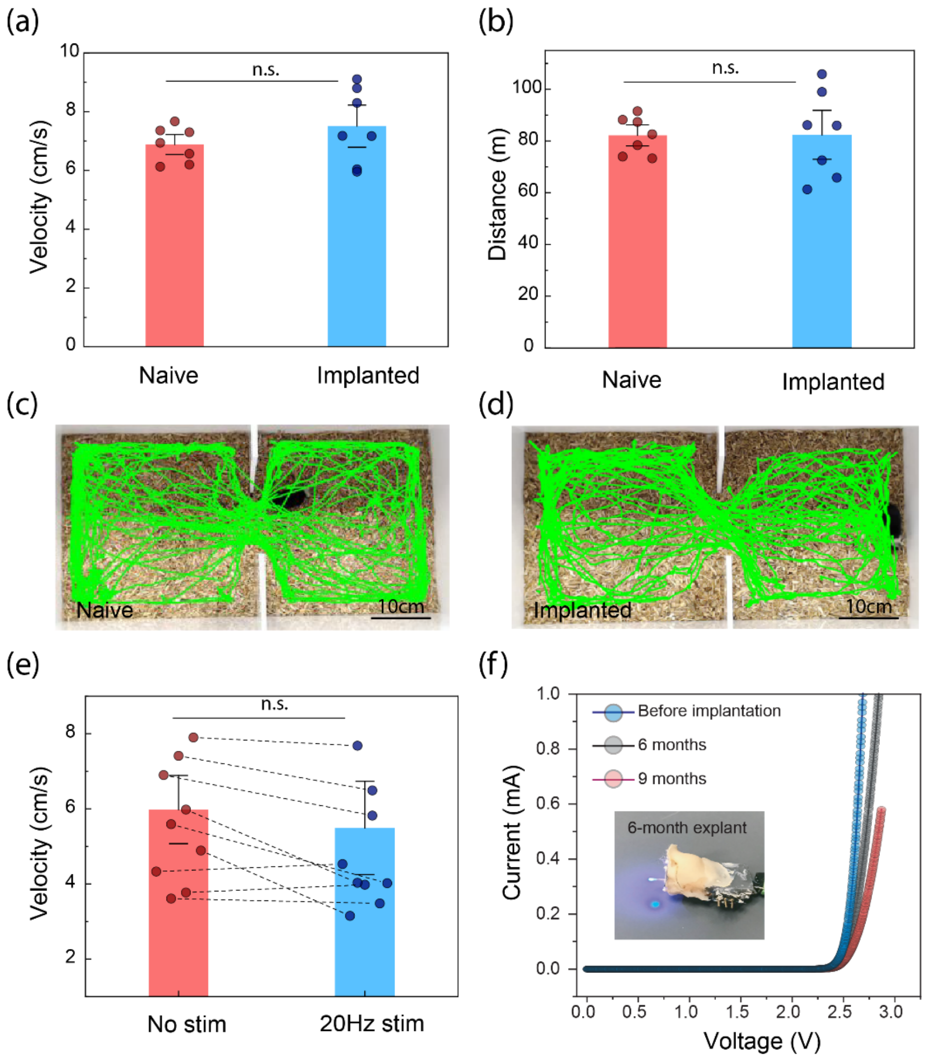

Figure S24. Control behavior experiments and characterization of explanted devices

Analysis of overall locomotor activity of brain implanted mice carrying NeuroStack module in the behavior chamber as compared to naïve controls: **(a)** Velocity; p = 0.26841, t = -1.1836, d.f. = 8.55636 (n=7 mice, Welch's t-test); and **(b)** distance travelled; p = 0.97869, t = - 0.02754, d.f = 8.16039 (n=7 mice, Welch's t-test); Position tracking in a representative run for **(c)** naïve subject; **(d)** module carrying implanted subject; **(e)** No significant changes in velocity of ChR2-mCherry mice was detected in response to 20 Hz stimulation (470 nm, 25 Hz, 10 ms pulse, 1 s ON, 2 s Off) as compared to without stimulation; p = 0.25085, t = 1.22729, d.f. = 8 (n=9 mice, two-tailed paired samples t-test); **(f)** Current-voltage characteristics of fiber µLED from devices that were explanted at 6 and 9 months as compared to pre-implantation. Data are presented as mean values +/- s.d. All error bars represent s.d.

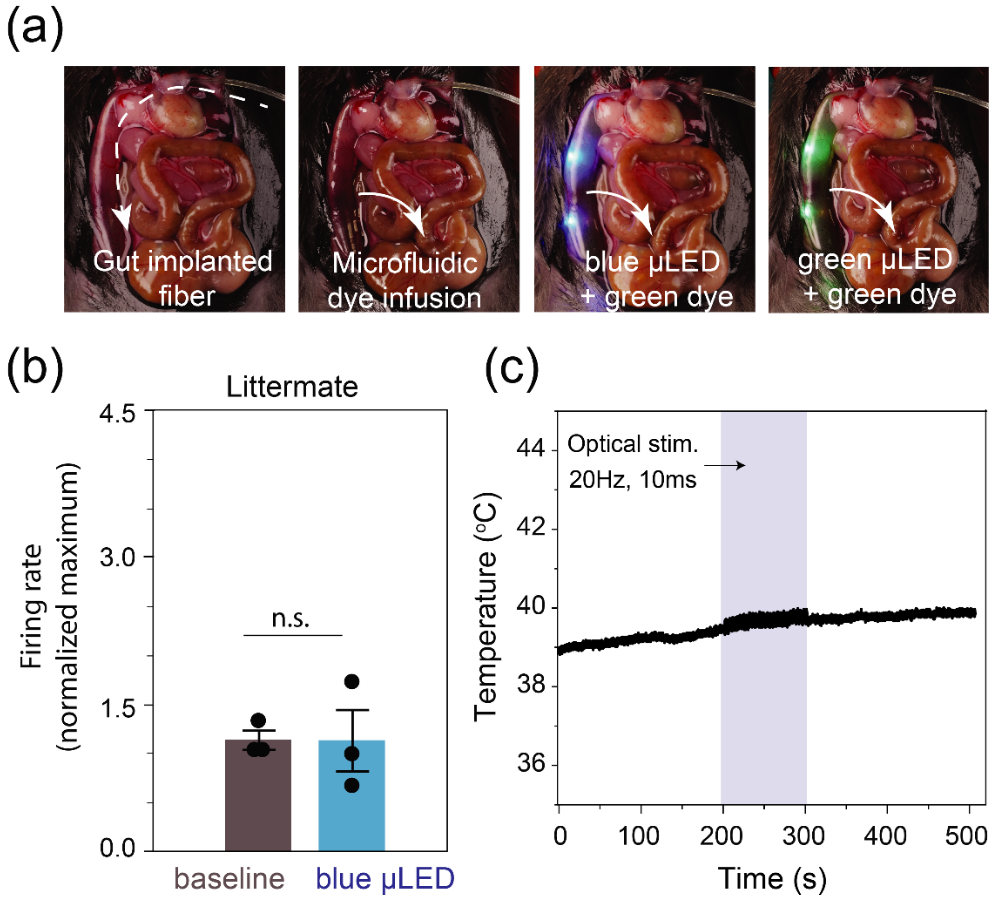

Figure S25. Optofluidic modality of gut fiber, control vagal recordings, and intraluminal gut temperature recording

(a) Dual optofluidic modality of a gut fiber implanted in the proximal small intestine, showing microfluidic infusion of a green dye solution, and simultaneous operation of blue and green µLEDs in the same fiber; **(b)** Quantification of vagal responses upon blue µLED stimulation in control littermates that lacked ChR2 in Cck cells (n=3 mice per group; *p = 0.6625 by Kruskal-Wallis test with non-parametric comparisons using Wilcoxon Method); **(c)** Intraluminal gut temperature recording in a chronically implanted animal before, during (shaded), and after optical stimulation epoch. Data are presented as mean values +/- s.em. All error bars represent s.e.m.

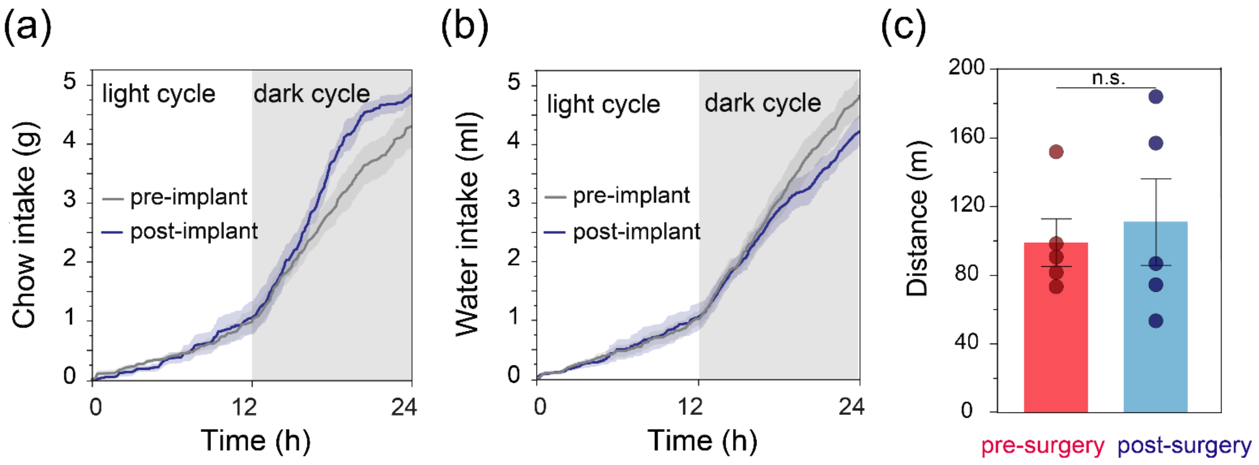

Figure S26. Feeding and locomotor behavior of mice chronically implanted with soft gut fiber in the proximal duodenum

(a) Feeding, and **(b)** drinking behavior is not affected by chronic implantation of the soft gut fibers in the small intestine; **(c)** Locomotor activity of mice in the homecage environment in the first 30 minutes of the dark cycle, pre- and post-implantation of gut fibers in the duodenum. Two-sided paired t-test, $p = 0.717$, $t = 0.389$, $d.f = 4$ (n=5 mice). Data are presented as mean values +/- s.e.m. All shaded areas and error bars represent s.e.m.

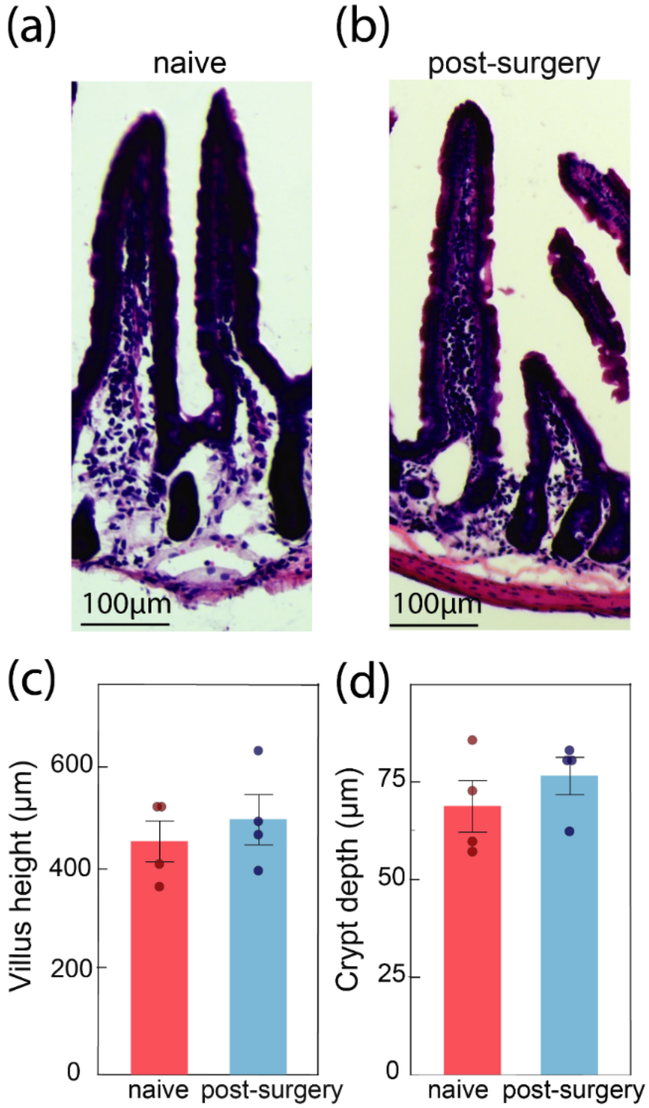

Figure S27. Histological assessment of biocompatibility of chronically implanted gut fibers

Optical micrographs of Hematoxylin and eosin (H&E) stained gut epithelial tissue from **(a)** naïve; and **(b)** gut implanted mice 1-week post-surgery; **(c)** Villi height (p = 0.5291, n=4 mice) and **(d)** crypt depth (p = 0.3789, n=4 mice) did not vary significantly for naïve and fiber-implanted mice as confirmed by two-sided t-test, indicating an intact and non-inflamed gut epithelium. Data are presented as mean values +/- s.e.m.

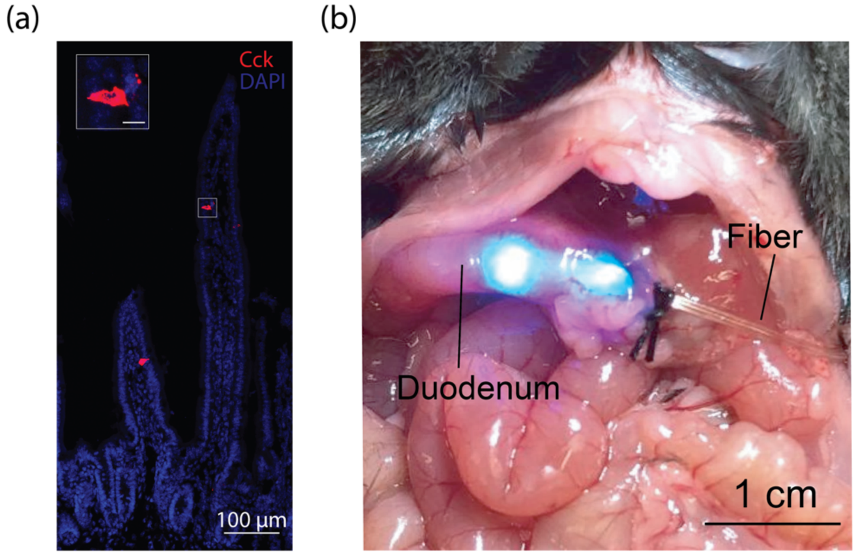

Figure S28. Visualization of the sparsely distributed cholecystokinin (Cck+) neuropod cells and the gut fiber during operation in the duodenum

(a) Confocal micrograph showing sparsely distributed Cck+ neuropod cells in the epithelial layer of the mouse duodenum. Inset shows Cck+ neuropod cell. Inset scale bar = 10 µm. Blue; DAPI; red; tdTomato; **(b)** Representative photograph of a chronically implanted gut fiber in the duodenum during blue µLED stimulation.

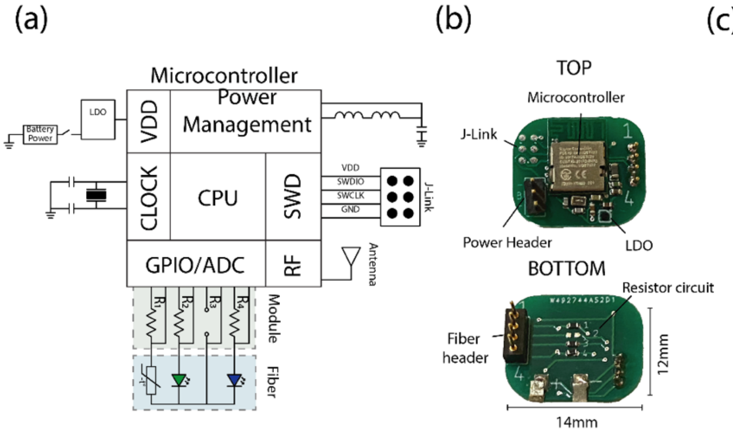

(d)

Code	Component
C_1	12pF
C_2	10µF
C_3	1µF
C_1	10µH
C_2	15nH
R_1, R_2, R_3, R_4	Depends on corresponding fiber components:<br>• LED: 0Ω<br>• NTC: 10kΩ<br>• Ground: No Resistor
LDO	LP5907UVE 3.3V Linear Regulator
X1	ABS05W 32.768kHZ Crystal
Battery	GM470910/GM041215
Microcontroller	MDBTV-P512KV2

Figure S29. Modified wireless module that enables prolonged operation during feeding behavior

(a) Block diagram of the modified wireless module used for feeding experiments; **(b)** Top and bottom views of a fully functional module; **(c, d)** parts list for the module along with their positions on the circuit.

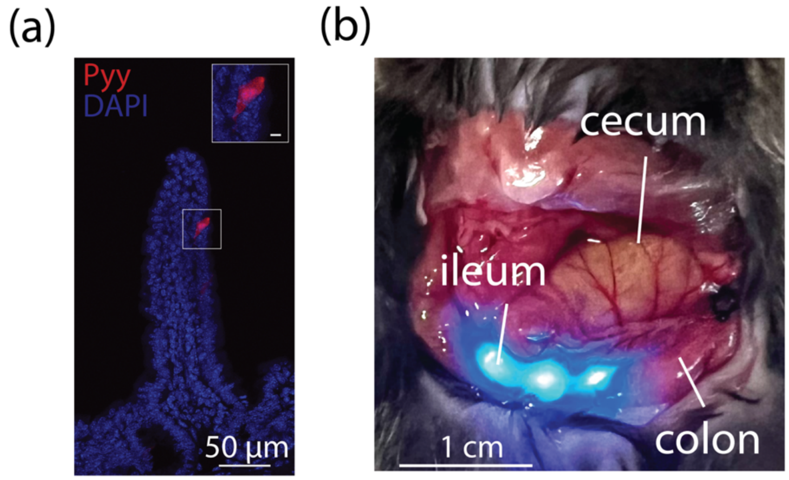

Figure S30. Visualization of the sparsely distributed Peptide YY (Pyy+) neuropod cells in gut epithelium and gut fiber in operation in the ileum

(a) Confocal micrographs of Pyy+ neuropod cells in the epithelial layer of the mouse ileum. Inset shows Pyy+ neuropod cell. Inset scale bar = 10 µm. Blue; DAPI; red; tdTomato; **(b)** Representative photograph of a chronically implanted gut fiber in the ileum during blue µLED stimulation.

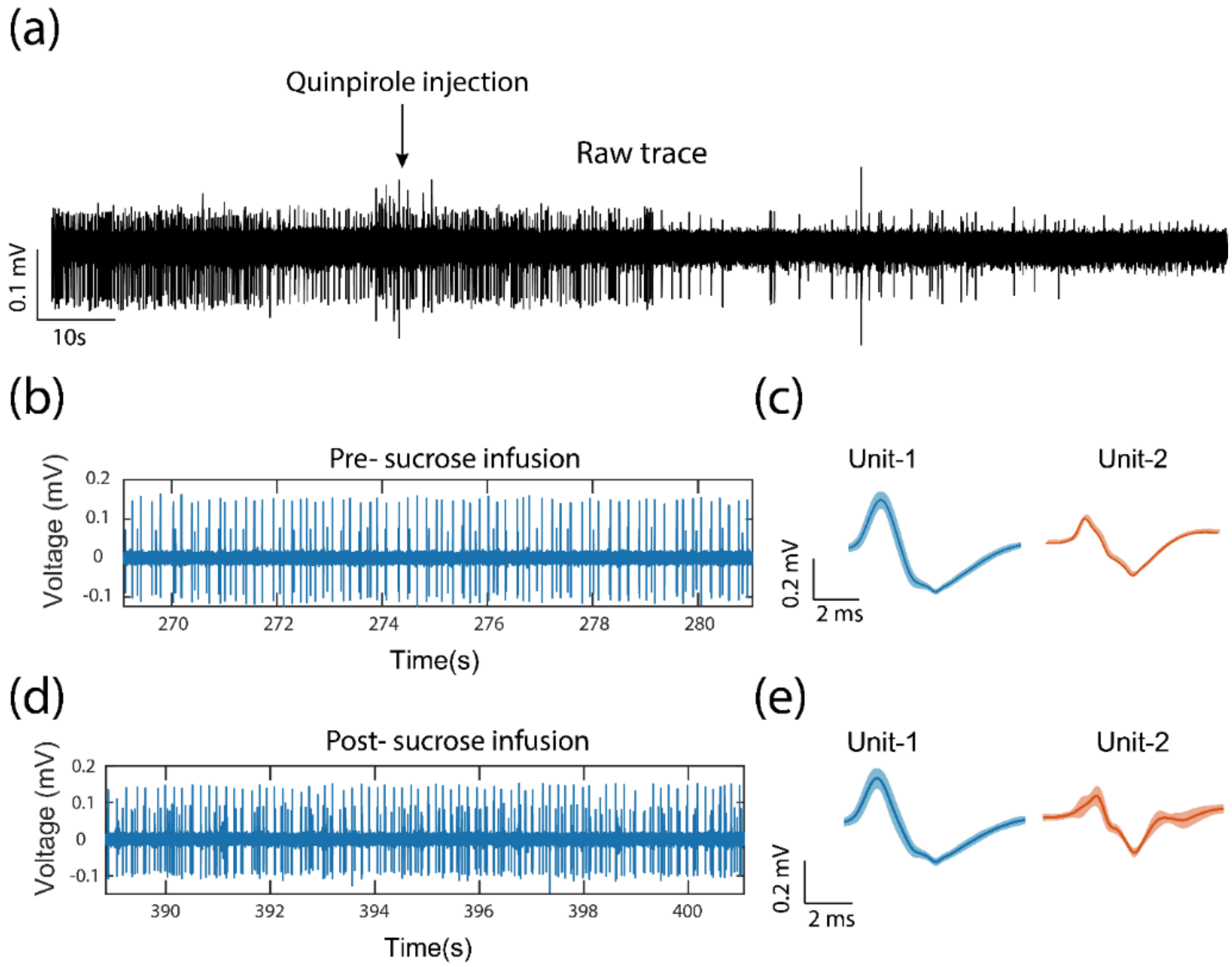

Figure S31. Electrophysiological recordings in the VTA via an implanted brain fiber pre- and post- sucrose infusion into the gut lumen via an implanted gut fiber

(a) Raw trace recorded from the VTA of a representative animal showing the sensitivity of recorded putative DA neuron to systemic injection of D2 autoreceptor agonist quinpirole; **(b)** Raw trace from a representative animal before intestinal sucrose infusion; **(c)** corresponding extracted units; **(d)** Raw trace from the same animal after intestinal sucrose infusion; **(e)** corresponding extracted units.

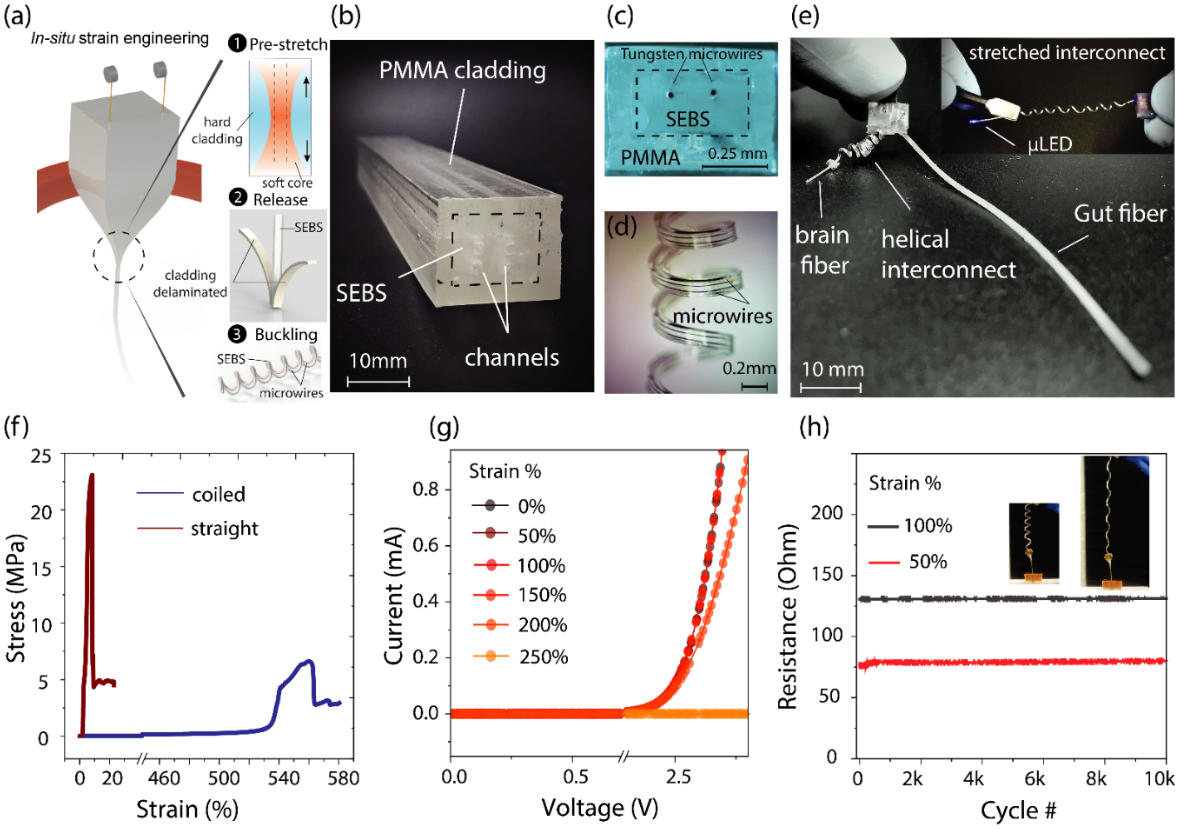

Figure S32. Thermally drawn stretchable interconnects enable multisite devices

(a) Schematic illustration of *in-situ* strain engineering during thermal drawing process that deterministically produces helically buckled fibers; **(b)** Preform geometry and composition; **(c)** cross-sectional micrograph of the drawn fiber with intact cladding; **(d)** optical micrograph of a helically coiled fiber upon cladding removal; **(e)** A photograph of a multisite, multifunctional gut-brain device. Inset shows the brain fiber with an operating μLED during uniaxial stretching of the interconnect; **(f)** Stress-strain plots for a helically coiled stretchable interconnect and a straight fiber instrumented with identical metal microwires; **(g)** Current-voltage characteristic of a blue μLED bonded to the stretchable interconnect at different values of uniaxial strain; **(h)** Cyclic stability of the stretchable interconnect over 10^4 cycles of continuous stretching to 50% and 100% strain while measuring the resistance from a micro resistor chip connected to the fiber.

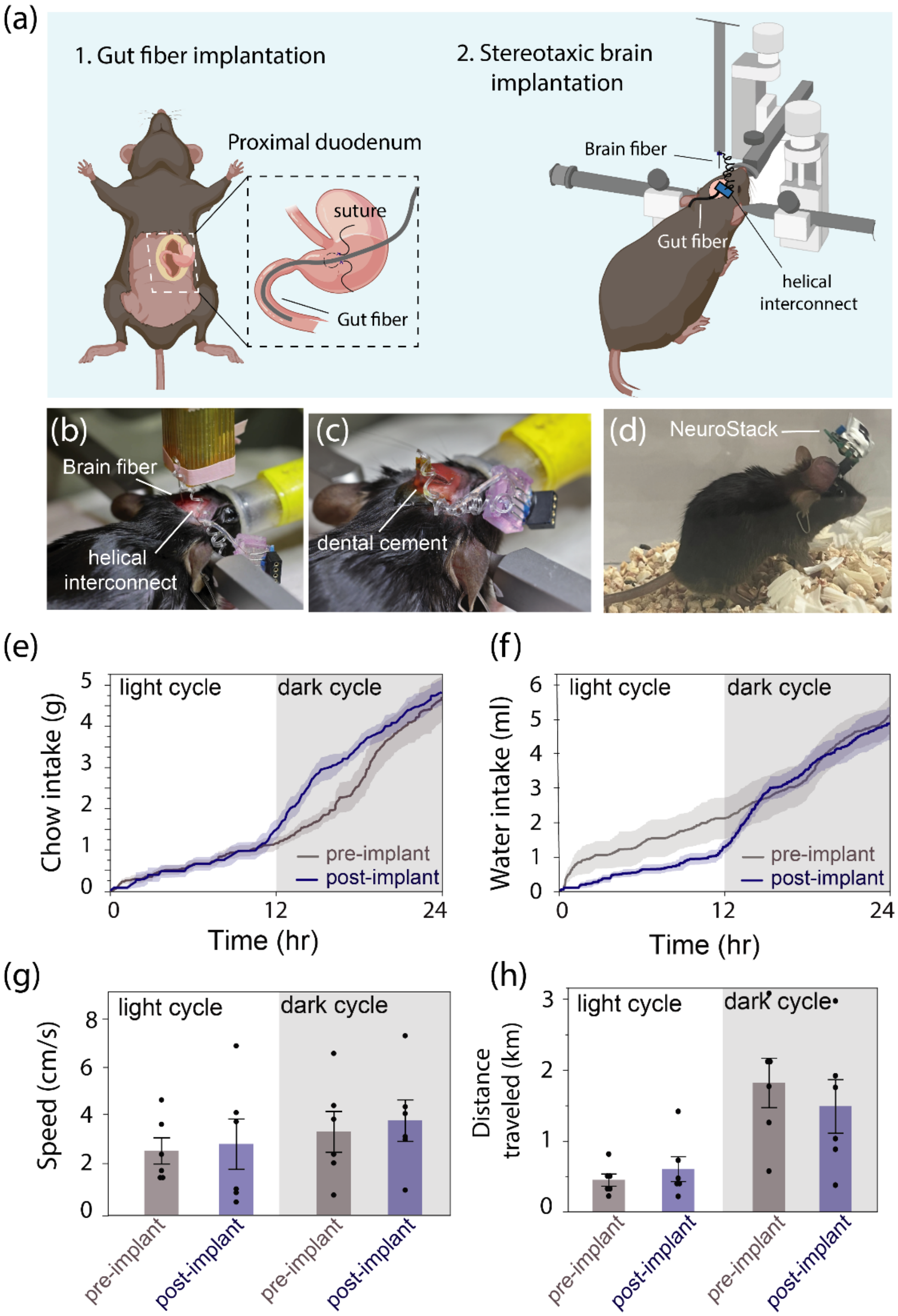

Figure S33. Compatibility of multisite devices with long-term implantation

(a) Schematic illustration of the surgical implantation procedure of a multisite, multifunctional microelectronic fiber device in the proximal small intestine and the VTA; **(b, c)** Corresponding photographs showing the stretchable interconnect allowing stereotactic brain implantation; **(d)** A

photograph of a fully recovered animal following multisite implantation surgery with the wireless module attached; **(e)** Food ($p = 0.8940$) and **(f)** water intake ($p = 0.7849$) of mice chronically implanted in the gut and brain (n=4) are unaffected compared to their values pre-surgery by two-sided paired t-test; the locomotor activity of the dually implanted mice (n=6) pre- and post-surgery remain unaffected as measured via **(g)** speed ($p = 0.9106$) and **(h)** distance travelled ($p = 0.7511$) over 24 h by two-sided t-test. Data are presented as mean values +/- s.e.m. All shaded areas and error bars represent s.e.m.

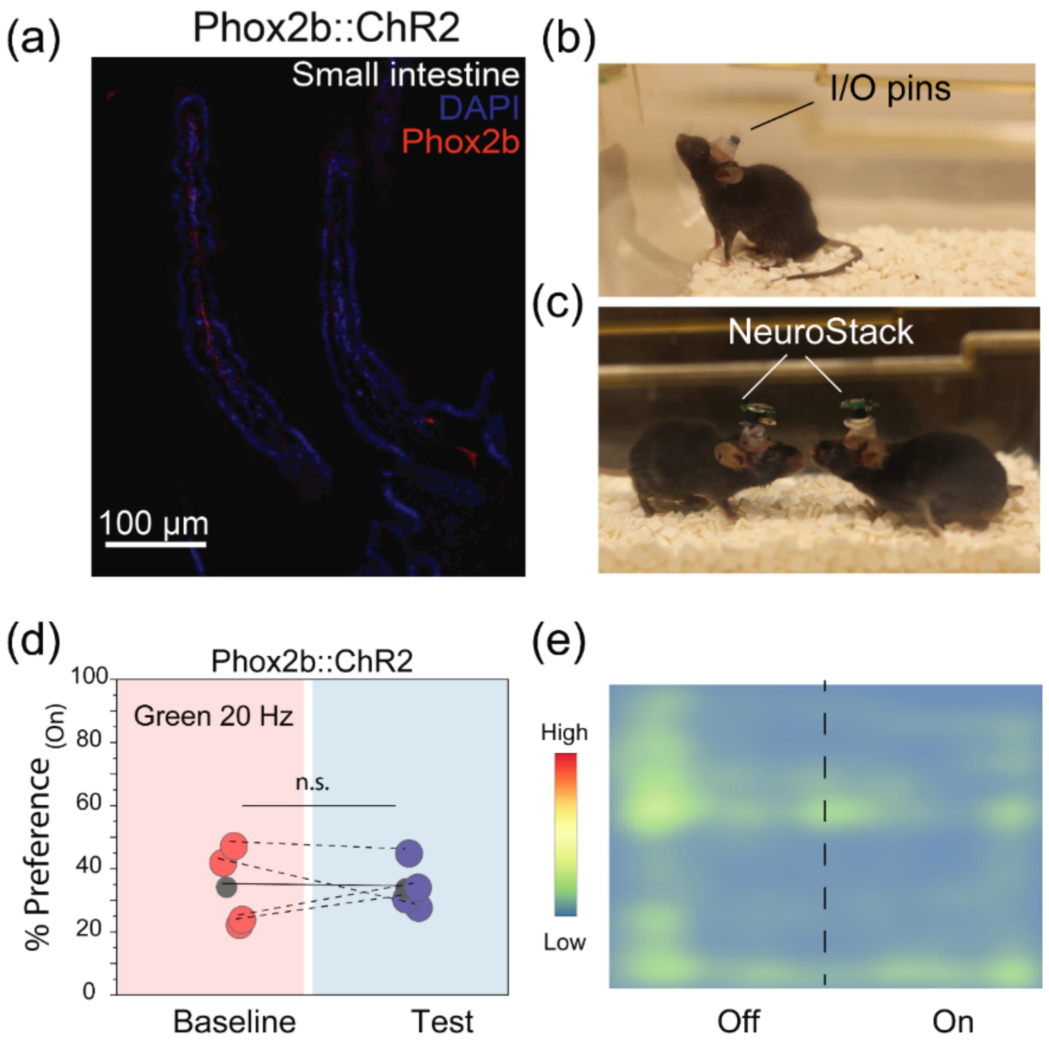

Figure S34. Visualization of Phox2b+ vagal afferents in the duodenum and place-preference behavior controls

(a) Confocal micrographs of Phox2b+ vagal afferents innervating the duodenal villi of a Phox2b::tdTomato mouse. Blue; DAPI; red; tdTomato; **(b)** A photograph of a mouse implanted with a gut fiber in the intestinal lumen during postoperative recovery in the home cage; **(c)** a pair of chronically implanted mice carrying NeuroStack modules; **(d)** Preference (%) in the chamber coupled to green µLED stimulation at baseline and on test-day for Phox2b::ChR2 mice ($\lambda = 470$ nm 20 Hz, 10 ms pulse, 0.5 s On duration, 1 s Off duration). Two-sided paired t-test, $p = 0.967$, $t = 0.045$, $d.f = 4$, **(e)** Corresponding representative activity heat map.

Table S1: Comparison of state-of-the-art wireless multifunctional bioelectronic interfaces.

Ref.	Fabrication method	Functions	CNS Organ	Visceral Organ	Powering scheme	Interfacing	Real-time Programming	Wireless data transfer
19	Photolithography	Optical stim<br>Photo sensing<br>Thermal sensing<br>Electrophysiology	✓ (Brain)	✘	RF	Detachable plug-n-play	✘	✘
22	Photolithography	Optical stim<br>Strain sensing	✘	✓ (Bladder)	Inductive coupling	Subdermal implantation	✘	✓
23	Photolithography	Optical stim<br>Pharmacology	✓ (Brain)	✘	Battery powered	Head mounted	✘	✘
24	Photolithography	Optical stim<br>Pharmacology	✘	✓ (Sciatic nerve)	RF	Subdermal implantation	✘	✘
25	Photolithography	Optical stim<br>Pharmacology	✓ (Brain)	✘	Battery powered	Head mounted	✓	✘
This work	Thermal drawing	Optical stim<br>Pharmacology<br>Electrophysiology<br>Thermal sensing	✓ (Brain)	✓ (GI tract)	Battery powered	Detachable plug-n-play	✓	✓

Videos

Video V1: A representative video of the scalable fiber fabrication process using thermal drawing showing convergence of metal interconnects into a preform.

Video V2: Independently addressable blue and green μLEDs and programmable control of stimulation frequency in a brain fiber.

Video V3: Independently addressable blue and green μLEDs and programmable control of stimulation frequency in a gut fiber.

Video V4: Independently addressable multicolor μLEDs in a brain and gut fiber highlighting the capability of simultaneously controlling multiple devices implanted in distinct anatomical regions.

Video V5: A representative video of a mouse implanted with a microelectronic brain fiber in the ventral tegmental area and carrying NeuroStack module in an open field chamber.

Video V6: A representative video of a pair of Phox2b::ChR2 mice implanted with a gut fiber carrying NeuroStack module ambulating in the home cage.

Video V7: A representative video of a Pyy::ChR2 mouse chronically implanted with a microelectronic gut fiber in the ileum and connected to NeuroStack drinking high fat solution in the home cage.

References

1. Klapoetke, N. C. *et al.* Independent optical excitation of distinct neural populations. *Nat. Methods* **11**, 338–346 (2014).

2. Mattis, J. *et al.* Principles for applying optogenetic tools derived from direct comparative analysis of microbial opsins. *Nat. Methods* **9**, 159–172 (2012).

3. Yang, Y. *et al.* Wireless multilateral devices for optogenetic studies of individual and social behaviors. *Nat. Neurosci. 2021 247* **24**, 1035–1045 (2021).

4. Canales, A., Park, S., Kilias, A. & Anikeeva, P. Multifunctional Fibers as Tools for Neuroscience and Neuroengineering. *Acc. Chem. Res.* **51**, 829–838 (2018).

5. Park, S. *et al.* One-step optogenetics with multifunctional flexible polymer fibers. *Nat. Neurosci.* **20**, 612–619 (2017).

6. Guo, Y. *et al.* Polymer Composite with Carbon Nanofibers Aligned during Thermal Drawing as a Microelectrode for Chronic Neural Interfaces. *ACS Nano* **11**, 6574–6585 (2017).

7. Rein, M. *et al.* Self-assembled fibre optoelectronics with discrete translational symmetry. *Nat. Commun.* **7**, 12807 (2016).

8. Shabahang, S., Kaufman, J. J., Deng, D. S. & Abouraddy, A. F. Observation of the Plateau-Rayleigh capillary instability in multi-material optical fibers. *Appl. Phys. Lett.* **99**, 161909 (2011).

9. Loke, G., Yan, W., Khudiyev, T., Noel, G. & Fink, Y. Recent Progress and Perspectives of Thermally Drawn Multimaterial Fiber Electronics. *Adv. Mater.* **32**, 1904911 (2020).

10. Rein, M. *et al.* Diode fibres for fabric-based optical communications. *Nature* **560**, 214–218 (2018).

11. Yoo, S. *et al.* Wireless Power Transfer and Telemetry for Implantable Bioelectronics. *Adv. Healthc. Mater.* **10**, 2100614 (2021).

12. Singer, A. & Robinson, J. T. Wireless Power Delivery Techniques for Miniature Implantable Bioelectronics. *Adv. Healthc. Mater.* **10**, 2100664 (2021).

13. Shin, G. *et al.* Flexible Near-Field Wireless Optoelectronics as Subdermal Implants for Broad Applications in Optogenetics. *Neuron* **93**, 509-521.e3 (2017).

14. Gutruf, P. *et al.* Fully implantable optoelectronic systems for battery-free, multimodal operation in neuroscience research. *Nat. Electron. 2018 112* **1**, 652–660 (2018).

15. Hossain, M. F. & Takahashi, T. Effect of annealing temperature on nanostructured WO3 films on P-Si substrate. in *Procedia Engineering* vol. 56 702–706 (Elsevier Ltd, 2013).

16. Kanik, M. *et al.* Strain-programmable fiber-based artificial muscle. *Science (80-.).* **365**, 145–150 (2019).

17. McCall, J. G. *et al.* Fabrication and application of flexible, multimodal light-emitting devices for wireless optogenetics. *Nat. Protoc.* **8**, 2413–2428 (2013).

18. McCall, J. G. *et al.* Preparation and implementation of optofluidic neural probes for in vivo wireless pharmacology and optogenetics. *Nat. Protoc.* **12**, 219–237 (2017).

19. Kim, T. -i. *et al.* Injectable, Cellular-Scale Optoelectronics with Applications for Wireless Optogenetics. *Science (80-.).* **340**, 211–216 (2013).

20. McCall, J. G. *et al.* Fabrication and application of flexible, multimodal light-emitting devices for wireless optogenetics. *Nat. Protoc.* **8**, 2413–2428 (2013).

21. Seymour, J. P., Wu, F., Wise, K. D. & Yoon, E. State-of-the-art mems and microsystem tools for brain research. *Microsystems Nanoeng.* **3**, 1–16 (2017).

22. Mickle, A. D. *et al.* A wireless closed-loop system for optogenetic peripheral neuromodulation. *Nat. 2019 5657739* **565**, 361–365 (2019).

23. Jeong, J.-W., Mccall, J. G., Huang, Y., Bruchas, M. R. & Rogers, J. A. Wireless Optofluidic Systems for Programmable In Vivo Pharmacology and Optogenetics. *Cell* **162**, 662–674 (2015).

24. Zhang, Y. *et al.* Battery-free, fully implantable optofluidic cuff system for wireless optogenetic and pharmacological neuromodulation of peripheral nerves. *Sci. Adv.* **5**, eaaw5296 (2019).

25. Qazi, R. *et al.* Wireless optofluidic brain probes for chronic neuropharmacology and

photostimulation. *Nat. Biomed. Eng. 2019 38* **3**, 655–669 (2019).
