# Supplementary material for: Multifunctional microelectronic fibers enable wireless modulation of gut and brain neural circuits
Source: Nat Biotechnol. 2023 Jun 22;42(6):892–904. doi: 10.1038/s41587-023-01833-5 (PMC11180606; doi:10.1038/s41587-023-01833-5)
Supplement: Supplementary file 2 — Reporting Summary [file 41587_2023_1833_MOESM2_ESM.pdf]

nature research

Corresponding author(s): Polina Anikeeva

Last updated by author(s): May 3, 2023

Reporting Summary

Nature Research wishes to improve the reproducibility of the work that we publish. This form provides structure for consistency and transparency in reporting. For further information on Nature Research policies, see our Editorial Policies and the Editorial Policy Checklist.

Statistics

For all statistical analyses, confirm that the following items are present in the figure legend, table legend, main text, or Methods section.

n/a	Confirmed	
☐	☒	The exact sample size (*n*) for each experimental group/condition, given as a discrete number and unit of measurement
☐	☒	A statement on whether measurements were taken from distinct samples or whether the same sample was measured repeatedly
☐	☒	The statistical test(s) used AND whether they are one- or two-sided<br>*Only common tests should be described solely by name; describe more complex techniques in the Methods section.*
☐	☒	A description of all covariates tested
☐	☒	A description of any assumptions or corrections, such as tests of normality and adjustment for multiple comparisons
☐	☒	A full description of the statistical parameters including central tendency (e.g. means) or other basic estimates (e.g. regression coefficient) AND variation (e.g. standard deviation) or associated estimates of uncertainty (e.g. confidence intervals)
☐	☒	For null hypothesis testing, the test statistic (e.g. *F*, *t*, *r*) with confidence intervals, effect sizes, degrees of freedom and *P* value noted<br>*Give P values as exact values whenever suitable.*
☒	☐	For Bayesian analysis, information on the choice of priors and Markov chain Monte Carlo settings
☒	☐	For hierarchical and complex designs, identification of the appropriate level for tests and full reporting of outcomes
☒	☐	Estimates of effect sizes (e.g. Cohen's *d*, Pearson's *r*), indicating how they were calculated

Our web collection on statistics for biologists contains articles on many of the points above.

Software and code

Policy information about availability of computer code

Data collection	LabView (2018 version 18.0) was used to control thermal draw tower and perform impedance tests. CorrWare 3.5i was used for I-V characterization of fibers. Advantage 5.5 Software (TA Instruuments) was used to collect bending stiffness data. COMSOL Multiphysics (version 5.6) was used for thermal and optical modeling. ABAQUS 2019 was used for mechanical modeling. The firmware code for controlling and communicating with the peripheral board was developed using Nordic Software Development Kit and Segger Embedded Studio Software. The NeuroStack firmware code was developed in Arduino IDE and loaded onto the module using Adalink Tool Kit. TSE PhenoMaster (Software version 7.3.3) was used to collect data on food intake, water intake, and locomotion in gut implant experiments. Raw vagal extracellular recordings were collected using Signal Express, National Instruments Corp.
Data analysis	FIJI (ImageJ 1.53g) was used to analyze immune response from implanted fibers by quantifying the fluorescence area. TA Universal Analysis was used to analyze bending stiffness data. Mouse behavior analysis was performed using EthoVision XT (Noldus) and TSE PhenoMaster (Software version 7.3.3). Brain and vagal electrophysiology data were analyzed using MATLAB (R2019b). Plots were generated using OriginPro 2021b. OriginPro 2021b or JMP Pro 15 and 16 software was used to assess the statistical significance of all comparison studies.

For manuscripts utilizing custom algorithms or software that are central to the research but not yet described in published literature, software must be made available to editors and reviewers. We strongly encourage code deposition in a community repository (e.g. GitHub). See the Nature Research guidelines for submitting code & software for further information.

Data

Policy information about availability of data

All manuscripts must include a data availability statement. This statement should provide the following information, where applicable:

- Accession codes, unique identifiers, or web links for publicly available datasets
- A list of figures that have associated raw data
- A description of any restrictions on data availability

Source data for Figures 2-6 as well as for the corresponding Supplementary Figures are provided with this paper. The custom software script, microcontroller firmware code, and NRF52840 Development Kit firmware code used in this work are available at: https://github.com/HarrisonAllen/Wireless-Controllers-for-Microelectronic-Fibers

Field-specific reporting

Please select the one below that is the best fit for your research. If you are not sure, read the appropriate sections before making your selection.

☒ Life sciences ☐ Behavioural & social sciences ☐ Ecological, evolutionary & environmental sciences

For a reference copy of the document with all sections, see nature.com/documents/nr-reporting-summary-flat.pdf

Life sciences study design

All studies must disclose on these points even when the disclosure is negative.

Sample size	Group sizes were chosen based on previous research conducted in the same brain circuit or intestinal region (ref. 34 and ref. 35)
Data exclusions	-Data from failed devices were excluded from the analysis for both brain and gut behavior studies.<br>-For brain real-time place preference behavioral studies, mice that showed more than 70% preference to any chamber on the pre-test day were excluded from subsequent analysis.<br>-For brain experiments, virus expression was confirmed postmortem and animals that lacked expression were excluded from the analysis
Replication	- Electrochemical impedance test was replicated twice per probe to establish electrochemical equilibrium upon immersion of the probe in 1XPBS before recording the final spectrum. Statistics is presented on results from independent devices<br>- I-V characteristics of the fiber integrated LEDs were acquired under a linear sweep voltametry mode in a 2-electrode configuration for 3 sweeps before recording the final I-V curve. Statistics is presented on results from independent devices<br>- In-vivo optoelectrophysiology recordings were replicated over at least 20 stimulation trials per animal<br>- For vagal electrophysiology experiments, the response to positive control sucrose was tested and replicated in between ligands to ensure within subject reproducibility. If the response changed substantially, the inclusion criterion was not met and therefore, the experiment was terminated.<br>- Fiber bending stiffness, calibration of thermal sensor, and microfluidic return rate measurements were not replicated on each device. Instead, reproducibility was confirmed by repeating the experiment on independent samples from different batches to ensure conserved longitudinal features in the several meters long fibers<br>- For behavior experiments, response to optogenetic stimulation was not replicated within subject because multiple experiments were required from each mouse. To ensure reproducibility, mice across at least 3 litters were used for each experiment.<br>- Replication attempts were deemed unsuccessful and excluded from analyses if devices failed during the test due to insulation failure or backend failure
Randomization	All devices were randomly assigned to experimental groups. Animals for gut and brain behavior studies were randomly assigned to treatment groups.
Blinding	- For analysis of animal behavior videos the experimenter was blinded to stimulation conditions and group allocation.<br>- For bench-top characterization of fibers and NeuroStack modules blinding could not be performed as the experimenter had to administer the test and had knowledge of control samples<br>- For in-vivo recording and optogenetic behavior studies experimenter was not blinded to treatment conditions, genotype, or outcome due to the need for the experimenter to administer the desired optical stimulation or nutrient infusion.

Reporting for specific materials, systems and methods

We require information from authors about some types of materials, experimental systems and methods used in many studies. Here, indicate whether each material, system or method listed is relevant to your study. If you are not sure if a list item applies to your research, read the appropriate section before selecting a response.

Materials & experimental systems

n/a	Involved in the study
☐	☒ Antibodies
☒	☐ Eukaryotic cell lines
☒	☐ Palaeontology and archaeology
☐	☒ Animals and other organisms
☒	☐ Human research participants
☒	☐ Clinical data
☒	☐ Dual use research of concern

Methods

n/a	Involved in the study
☒	☐ ChIP-seq
☒	☐ Flow cytometry
☒	☐ MRI-based neuroimaging

Antibodies

Antibodies used

Primary Antibody: Iba1:Goat anti-Iba1, ab107159 Abcam, 1:500 dilution; GFAP: Goat anti-GFAP, ab53554 Abcam, 1:1000 dilution.
Secondary antibody: Donkey anti-Goat Alexa Fluor 488, A11055, 1:1000, Thermofischer

Validation

The antibodies were validated in literature and in our laboratory for immunohistological staining on mouse brain slices (adult, C57BL/6). Specifically :
-Goat anti-Iba1 ab107159 was validated in mouse brain slice in S.Rao et. al. Nat. Nanotechnol. 14, 967–973 (2019)
-Goat anti-GFAP, ab53554 was validated in mouse brain slice in M.-J. Antonini et. al. Adv. Funct. Mater. 31, 2104857 (2021)

Animals and other organisms

Policy information about studies involving animals; ARRIVE guidelines recommended for reporting animal research

Laboratory animals

Wild type mice (C57BL/6) aged 6–8 weeks (Jackson Laboratory, Strain #:000664), DAT-ires-Cre (Jackson Laboratory Strain #006660) mice aged 6-10 weeks, Phox2b::ChR2 (breeding pairs obtained from Jackson Laboratory, Strain #: 016233; 012567), Pyy::ChR2 (Pyy::Cre mouse is courtesy of Andrew Leiter; Jackson Laboratory, Strain #: 012567), Cck::ChR2mice (breeding pairs obtained from Jackson Laboratory, Strain #012706; 012567) were used for this study. Approximately equal number of male and female mice were used. Mice were group housed before surgery and single housed after surgery in cages maintained at 22 C, 12 h light/dark cycle, and 50% humidity with ad libitum access to food and water

Wild animals

The study did not involve use of wild animals

Field-collected samples

The study did not involve samples collected from the field.

Ethics oversight

-All animal procedures performed at MIT were approved by the MIT Committee on Animal Care and carried out in accordance with the National Institutes of Health Guide for the Care and Use of Laboratory Animals.
-All animal procedures performed at Duke university were approved by the Duke University Institutional Animal Care and Use Committee and carried out in accordance with the National Institutes of Health Guide for the Care and Use of Laboratory Animals

Note that full information on the approval of the study protocol must also be provided in the manuscript.
